# The Boston Marathon versus the World Marathon Majors

**DOI:** 10.1371/journal.pone.0184024

**Published:** 2017-09-01

**Authors:** Philip B. Maffetone, Rita Malcata, Ivan Rivera, Paul B. Laursen

**Affiliations:** 1 Athlete Consultant, Oracle, AZ, United States of America; 2 Sports Performance Research Institute New Zealand (SPRINZ), AUT University, Auckland, New Zealand; 3 Research Assistant, San Diego, CA, United States of America; University of Chicago Medical Center, UNITED STATES

## Abstract

The Boston Marathon course is deemed ineligible for world record status due to its unidirectional nature and excessive negative elevation change, yet performance times across the World Marathon Majors (WMM) races have not been compared.

**Purpose:**

To compare finish times across WMM races for Boston, London, Berlin, Chicago and New York Marathons.

**Methods:**

Race times of the top 10 male and 10 female finishers were analyzed from 2005 to 2014 using the high-performance mixed linear model procedure in the Statistical Analysis System. Venue-to-venue comparisons, as well as comparisons between Boston and other WMM races, with and without factors of temperature, humidity and altitude change were examined.

**Results:**

Performance from 2005 to 2014 in the WMM races was found to improve at a rate of ~1% each 7 years. Despite its higher variability, comparison between Boston’s estimated mean finishing time and all other venues revealed moderate positive differences, indicating the Boston event to be typically slower than other venues.

**Conclusions:**

Across the 10-year study period, performance times improved ~1% each 7 years for both genders for the WMM, with the Boston Marathon being slower on average than other WMM venues. Weather rather than course metrics appeared to impact performance times most.

## Introduction

In the 1908 London Olympic Games, the first modern marathon was held over its official distance of 42.195 kilometers (26 miles and 365 yards) [1]. The current world record for men is held by Dennis Kimetto with a time 2:02:57 (h:min:s) at the 2014 Berlin Marathon [2], and for women by Paula Radclife with a time 2:15:25 at the 2003 London Marathon [3].

The Boston Marathon is the oldest annual marathon event in the world, with its inaugural race occurring in 1897. While marathon events were previously largely limited to the Olympics, the Boston Marathon and a few others, it was the inaugural 1976 New York City Marathon that appeared to mark a point in time where the world witnessed a dramatic increase in the number of marathon events, primarily in major cities as part of the worldwide running boom.

Unlike standardized 400-meter tracks, virtually all marathon courses, with the exception of total distance, inevitably display differences. Some are flatter, others undulating or more hilly, some may have a number of turns, and some may be unidirectional. Thus, certain courses are faster, and some may be slower.

**Fig 1:**
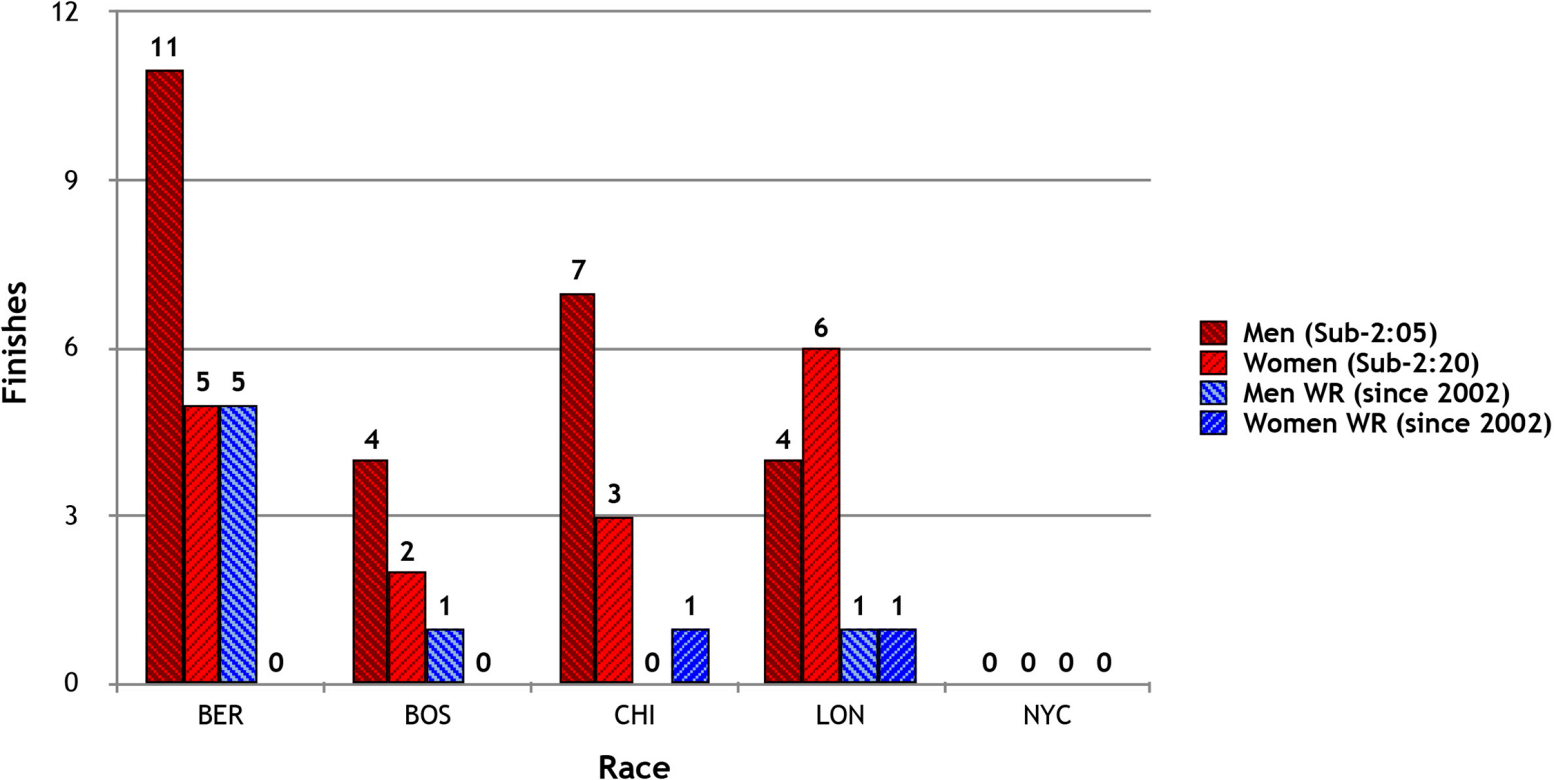
Fastest World Marathon WMM locations: World records and fastest times.

In 2005, the World Marathon Majors (WMM), a championship-style competition for marathon runners, began as an annual competition. The WMM comprises six existing marathon races that include Boston, London, Berlin, Chicago, New York and, starting in 2007, Tokyo. Best accumulated performances during any of the WMM events in each gender are awarded a cash prize (currently $500,000 US dollars), which, in addition to substantial prize money for top finishers of each race, helps assure that each race will attract elite marathoners. The all-time 50 fastest WMM times for men and women have been performed in Berlin, London, Chicago and New York, with the exception of one year in Boston (2011) and one year in Tokyo (2014) for men and one year in Boston (2014) and two years in Tokyo (2014 and 2016) for women (http://worldmarathonmajors.com) (Fig 1).

In 1912, the International Amateur Athletic Federation (IAAF) was founded in Stockholm, Sweden as the world governing body for the sport of track and field athletics events, including the marathon. Because the Boston Marathon course is a point-to-point (start-to-finish) race and has a net elevation loss that exceeds the IAAF’s limits, the IAAF have deemed the race to be not world-record eligible, according to IAAF Rule 260, established in 1990. The rule states that for a World Record in a road running event to be valid, it must contain the following relevant aspects:

1. The start and finish points of a course, measured along a theoretical straight line between them, shall not be further apart than 50% of the race distance.
2. The overall decrease in elevation between the start and finish shall not exceed 1:1000, i.e. 1m per km (0.1%) [4].

The Boston Marathon’s overall decrease in elevation that exceeds the IAAF limit would infer there is an excessive amount of downhill running and therefore may be an unfair advantage for faster finish times. In addition, Boston’s unidirectional course could have a tailwind for runners, if winds are out of the southwest, improving finish times. However, race times for elite men and women marathoners across the WMM have yet to be compared.

The purpose of the present study therefore was to compare finish times across the WMM races to identify the faster and slower races across both genders. For this, we analyzed the race times of the top 10 male and 10 female finishers of all races in the WMM for the years 2005–2014, including Boston (BOS), London (LON), Berlin (BER), Chicago (CHI) and New York (NYC). We also examined the potential influence of ambient temperature and elevation.

## Methods

To analyze, performance times, course information and weather conditions were retrieved through the official internet website for each city marathon, on marathon archive websites and from various media outlets (all public domain). The human research ethics committee of AUT University approved the study.

Marathon performance times were analyzed using the high-performance mixed linear model procedure (Proc Hpmixed) in the Statistical Analysis System (Versio 9.4, SAS Institute, Cary, NC). The fixed-model included a linear trend for calendar year, accounting for a general improvement of performance as result of better training and technology, and intercepts for the different race venues provided mean marathon performance times for each of the venues: BER, BOS, CHI, LON, NYC and TKO. The random effects in the model were Athlete, Raceid and BostonRace. The athlete effect estimates athletes’ running ability while addressing the repeated measurements structure of the data. Performances in each race (e.g., BOS 2014) were clustered by a Raceid random effect to account for the mean effect of environmental factors on performance times. The BostonRace random effect was included in the model to allow for extra variance for the Boston performances. Performance times were log-transformed to yield the effects and errors in percent change of the mean.

Venue-to-venue comparison as well as comparison between BOS and other WMM events were calculated and expressed as a percent factor. Magnitudes for the difference in mean performance times were assessed using a modified scale for standardized difference in means: thresholds for small, moderate, large, very large and extremely large were 0.2, 0.6, 1.2, 2.0 and 4.0 of the typical race-to-race variation. This typical race-to-race variation was determined as the standard deviation of the random effect for RaceId. Uncertainty on the estimates of performance and predicted mean times were shown as 90% confidence limits [5].

The Tokyo (TKO) Marathon was not part of the WMM until 2007, so we excluded that event from our analysis (as women’s performances were unusually slower in the early years, potentially due to the quality of the athletes). In addition, the 2012 NYC marathon data does not exist as the event was cancelled due to a hurricane.

The effect of temperature, humidity and altitude gain, loss and change were examined. Temperature and humidity information were collected from the Wunderground website and the altitude gain and loss were obtained from the official race profiles. While the magnitude effect of these factors was estimated, the main focus of the analysis was to understand whether or not these factors could further explain the performances. In particular, we were interested whether the inclusion of such factors would explain part of the variability between races. The model used to examine this was similar to that described above, however additional effects were included as fixed effects, and included: Venue*Temperature, Venue*Humidity (providing standardized weather conditions for each venue), as well as venue*altitudegain and venue*altitudeloss (investigating the impact of altitude gain and altitude loss). Random effects were specified as in the model described above. Venue was removed from the model as a fixed or random effect after it was revealed that it was an over-specified variable; i.e., venue effects were accounted for by temperature, humity and altitude effects.

## Results

**Fig 2:**
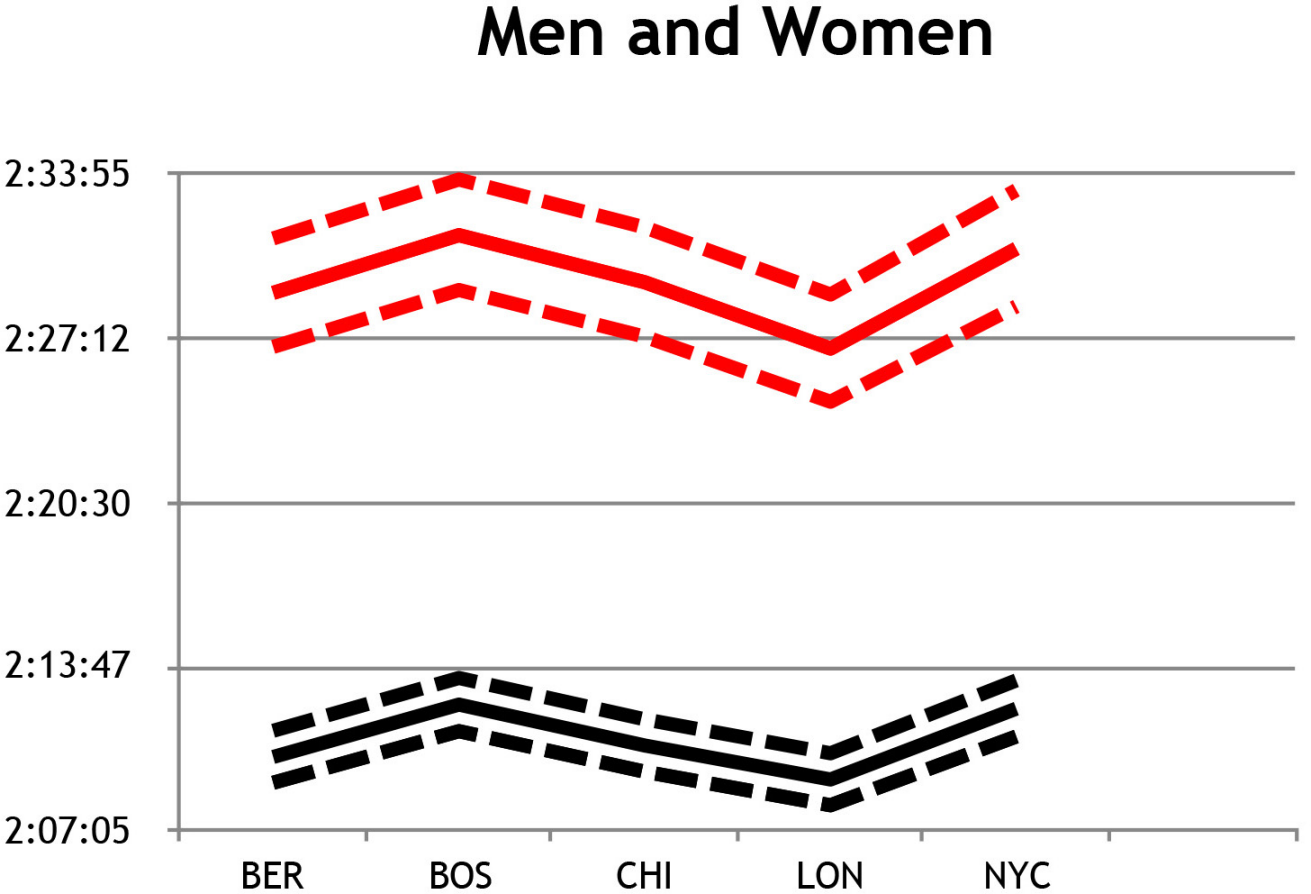
Estimated mean race times for the five major marathon venues, including Berlin (BER), Boston (BOS), Chicago (CHI), London (LON), and New York City (NYC), for male and female participants. Upper and lower Confidence Limits (CL) depicted as dashed lines.

Performance times of the five world major marathons were shown to improve from 2005 to 2014, with times decreasing at a typical rate of ~1% per year, for both males and females (Table 1). After properly accounting for calendar year improvements, the estimated mean times across the five different marathon venues are shown in Fig 2. Comparison of the estimated mean times between venues revealed moderate positive differences between BOS and all the other venues, with the exception of NYC. However, this positive difference between performance times was *unclear* for both male and female results, indicating that the BOS venue is typically slower than the other venues (i.e., mean times are typically greater than those of other venues).

**Table 1:** Improvement in performance times for the Boston (BOS), London (LON), Berlin (BER), Chicago (CHI) and New York (NYC) Marathons.

Effect		Effect	Lower	Upper	DF	Inference
***Women***						
7-year improvement in performance time^a^		-1.0%	-2.2%	0.1%	484	Small Positive
BOS vs…						
	ALL OTHERS	1.6%	0.4%	2.8%	484	Moderate Positive
	BER	1.5%	0.0%	3.0%	484	Moderate Positive
	CHI	1.3%	-0.2%	2.8%	484	Moderate Positive
	LON	3.1%	1.6%	4.7%	484	Large Positive
	NYC	0.3%	-1.2%	1.8%	484	Unclear
***Men***						
7-year improvement in performance time^a^		-1.1%	-1.9%	-0.2%	484	Moderate Positive
BOS vs…						
	ALL OTHERS	1.3%	0.4%	2.2%	484	Moderate Positive
	BER	1.6%	0.4%	2.7%	484	Moderate Positive
	CHI	1.3%	0.1%	2.4%	484	Moderate Positive
	LON	2.3%	1.1%	3.5%	484	Large Positive
	NYC	0.1%	-1.1%	1.3%	484	Unclear

^a^A negative slope represents an increase in performance times.

Typical race-to-race variability, that is the expected difference between mean performance times from one race to the next (e.g. Chicago 2005 vs. Berlin 2005) are presented in Table 2. Analysis revealed that the BOS venue showed higher variability from race to race in both genders. Interestingly, a *possible small* reduction in typical race-to-race variability was shown by the model when altitude change, temperature and humidity conditions were included.

**Table 2:** Race-to-race variability for World Marathon Majors compared to the Boston Marathon event, with and without including of temperature, humidity and altitude gain and loss factors.

	Simple Model		Including Temperature, Humidity, and Altitude Gain and Loss factors	
	mean	90% CL	mean	90% CL
***Women***				
Typical race-to-race variability	1.6%	1.3–2.1%	1.6%	1.2–2.2%
Boston race-to-race variability	2.8%	2.0–6.4%	2.2%	1.5–13.5%
***Men***				
Typical race-to-race variability	1.3%	1.1–1.7%	1.1%	0.7–2.2%
Boston race-to-race variability	1.9%	1.3–6.5%	1.8%	1.2–5.8%

## Discussion

We analyzed the race times of the top 10 male and 10 female finishers of all races in the WMM for the years 2005–2014, including BOS, LON, BER, CHI and NYC, but excluding TKO, to compare finish times and identify the faster and slower races across both genders. To the best of our knowledge, the present study is the first to analyze performance times across the WMM. While LON and BER were shown to be the first and second fastest WMM events, respectively, for both men and women, the top finishing times of men and women in BOS were shown to be typically slower than the other WMM venues. The high variation in BOS times suggests that perfect conditions (i.e., tailwind, optimal temperature) could cause an outlier time, which may have occurred in 2011. However, it is equally possible that the same ideal weather conditions could potentially arise at other WMM venues.

Analysis revealed that the BOS venue showed higher variability from race to race in men and women, with a *possible small* reduction in typical race-to-race variability when altitude change, temperature and humidity conditions were included. This suggests that there are other factors that affect performance times in these marathons, in particular for BOS. Such factors might include the presence of pacers, extent of prize money, as well as weather conditions that may contribute to performance differences between races. Furthermore, because BOS has a higher race-to-race variability, it may be that performance times are influenced to a larger degree by external factors such as weather, including wind conditions. However, the point-to-point nature of BOS, which the IAAF infers could produce unfair tailwinds, may have only been seen in the outlier year of 2011. That year produced a men’s world record time (unofficially), which was broken three years later in BER.

Previous studies have evaluated various marathons, both those within the WMM as well as other venues, demonstrating that performance of all finishers is inversely affected by air temperatures [6–9] but these studies have not evaluated other course factors or compared finish times between races. In the current study we analyzed the top 10 male and 10 female finishers to compare race performance between the WMM events. Our results support the notion that the Boston Marathon venue may not provide an unfair advantage due to its point-to-point course and elevation changes.

Like most marathons, the WMM events are run in different cities on local streets with varying terrains. Each course is uniquely different, unlike track events where the race tracks are all nearly identical. A variety of factors can impact race performance, including individual preparedness from training, nutrition, metabolic and mental-emotional states, and race-day nutrition and hydration practices. In addition, all individual marathon courses may offer other advantages and disadvantages for a faster or slower finish time, including road surface materials such as blacktop, concrete, steel/bridges, dirt, the presence of spectators and other factors, none of which are addressed here. However, because our data show that BER and LON demonstrate the fastest finish times for men and women, and the fact that BER, LON and CHI have a history of the most number of sub-2:05 finish times for men and sub-2:20 for women, and the most number of world-record times (see Fig 1), with BER having a much greater history of these finishes, any one or all three of these marathon venues could be considered advantageous for faster times. We do not know the reasons for such advantages but speculate that course dynamics and or weather may play a role.

Below we highlight some factors that can influence marathon performance, including environment, pacing and prize money. Net elevation change for all courses in the WMM is one of the two factors associated with the IAAF’s ruling eliminating BOS as a world record-eligible course and will be addressed first.

### Course elevation changes

**Fig 3:**
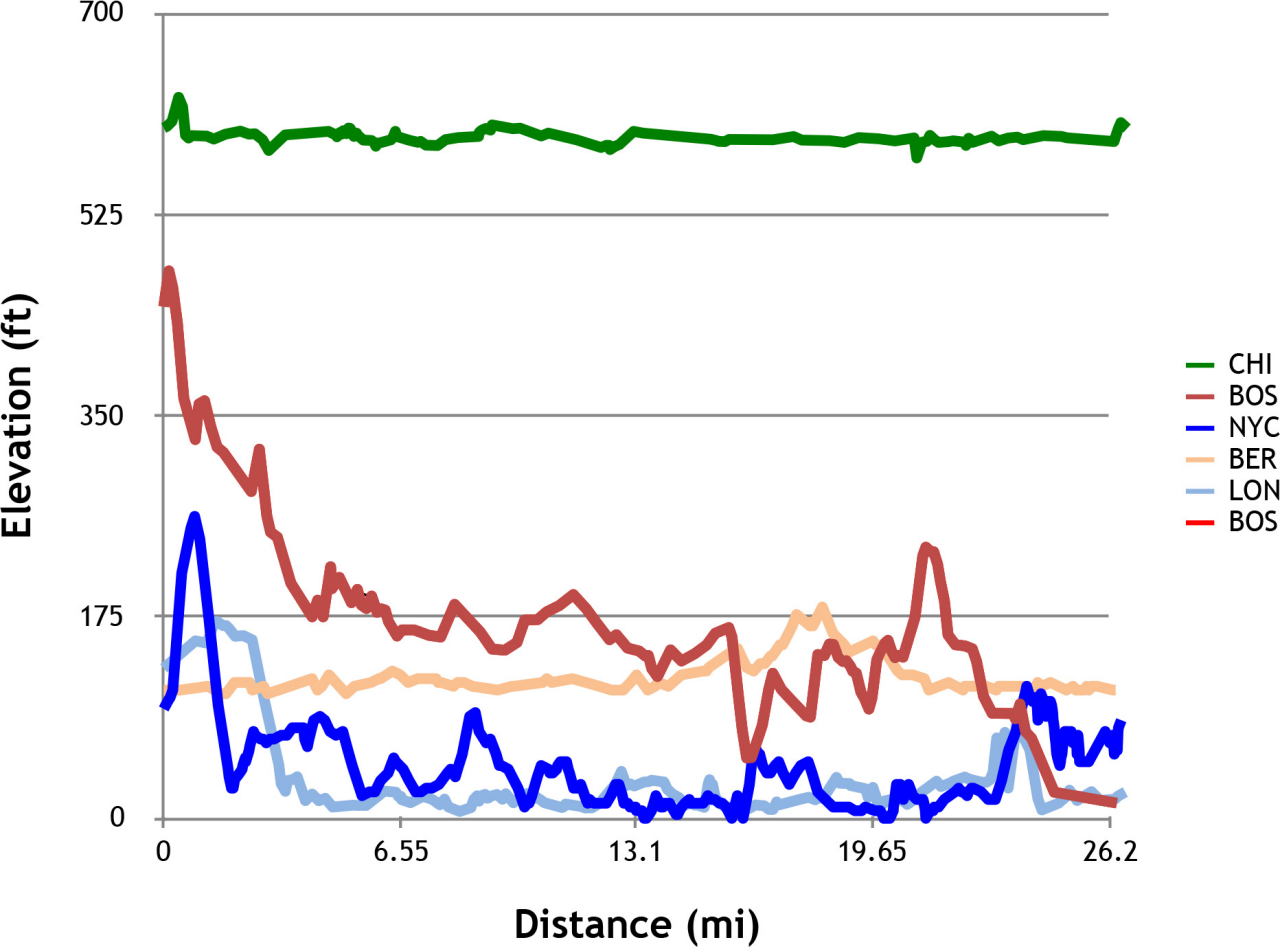
Course elevation profiles for Boston (BOS), London (LON), Berlin (BER), Chicago (CHI) and New York (NYC) Marathons.

The presumed reason for the IAAF’s Rule 260 regarding excess elevation changes is that an excess degree of downhill running may result in faster marathons (Fig 3). Contrary to this rationale, our results show that the BOS venue has relatively slow race times over the 10-year period of analysis of the 200 elite marathon finish times. In reality however, external factors such as weather can influence race times on all the WMM venues, with the 2011 BOS event revealed as being an outlier year.

### Environmental factors

Air temperature, humidity and air pollution can influence marathon race performance, affecting both genders equally, with cooler temperatures (5–10 degrees C) being most influential for better performance [6–9] by the fastest runners [7, 8]. While warmer temperatures can impair thermoregulation [10], Marino, Lamber [11] demonstrated that African runners, in part due to their smaller body size, had improved performance in warm humid versus cooler weather conditions compared to Caucasian runners.

### Pacing

Pacing in running has been described as the subjective competitive strategy associated with more even paces throughout a race that can improve running economy and enhance performance [12]. It is commonly known in competitive running that other runners are used as pacers in events from 800-meters and beyond, including the marathon [13] and, as coaches, athletes and others also know, many pacers are unofficial. It is difficult therefore to know which races, if any, are not paced by other runners. To prevent women from being paced by men during marathons, it is common practice today for separate-gender race starts (women first, then men). While most but not all marathons have used pacers, they are not allowed in World Championship or Olympic competitions, which may account for the few world record performances at those events. The use of pacers increased after two runners helped pace Roger Bannister to break the four-minute mile for the first time in 1954 [14, 15].

### Prize money

Race performance for elite runners may be influenced by prize money [16]. In addition to finishing in the top places and receiving cash awards, national-, course- and world-record times can contribute greatly to an athlete’s winnings, sponsorship bonuses, sponsorship contracts and other business opportunities important for a runner’s livelihood.

On any given WMM race day, ideal weather conditions could lead to faster finishing times and even world record times. Because BOS has a higher variability, it appears that race performances are influenced to a larger degree by external factors such as weather, including wind conditions. Excluding these weather factors, the relationship between course elevations and finish times does not indicate that BOS poses an unfair advantage, with the data suggesting that the 2011 BOS men’s finish times was an outlier. With both the fastest times for men and women, and the most world record times, the courses in BER and LON may pose an advantage for faster times in WMM venues.

## Conclusion

The Boston Marathon course is on average slower than other WMM venues, with a higher race-to-race variability than the other races. The higher variability means that performances at the Boston venue are less reliable. Weather, and not a downhill course topography, seems to be the significant race factor impacting performance of WMM events. The implication of our findings is that rules that pertain to weather are better at eliminating “fast” courses from world-record eligibility. Conversely, rules that pertain to course topography have less of an impact in eliminating fast courses. The latter rule instead may exclude a course based on features that have no significant impact on the course’s “speed.”

IAAF rules state:

1. The start and finish points of a course, measured along a theoretical straight line between them, shall not be further apart than 50% of the race distance.
2. The overall decrease in elevation between the start and finish shall not exceed 1:1000, i.e. 1m per km (0.1%) [4].

While no explanation is given in the IAAF rulebook as to the rationale behind each of these rules [4], it is understood that rule (a) is intended to eliminate courses with one prevailing heading, as a tailwind at any point of the course could be sustained the entire way. Similarly, it is understood that rule (b) is intended to eliminate courses where an overall downhill slope may confer runners a speed advantage over flatter courses. Our findings therefore suggest that rule (a) is more impactful in eliminating unfairly “fast” courses from world-record eligibility than rule (b).

**Fig 4:**
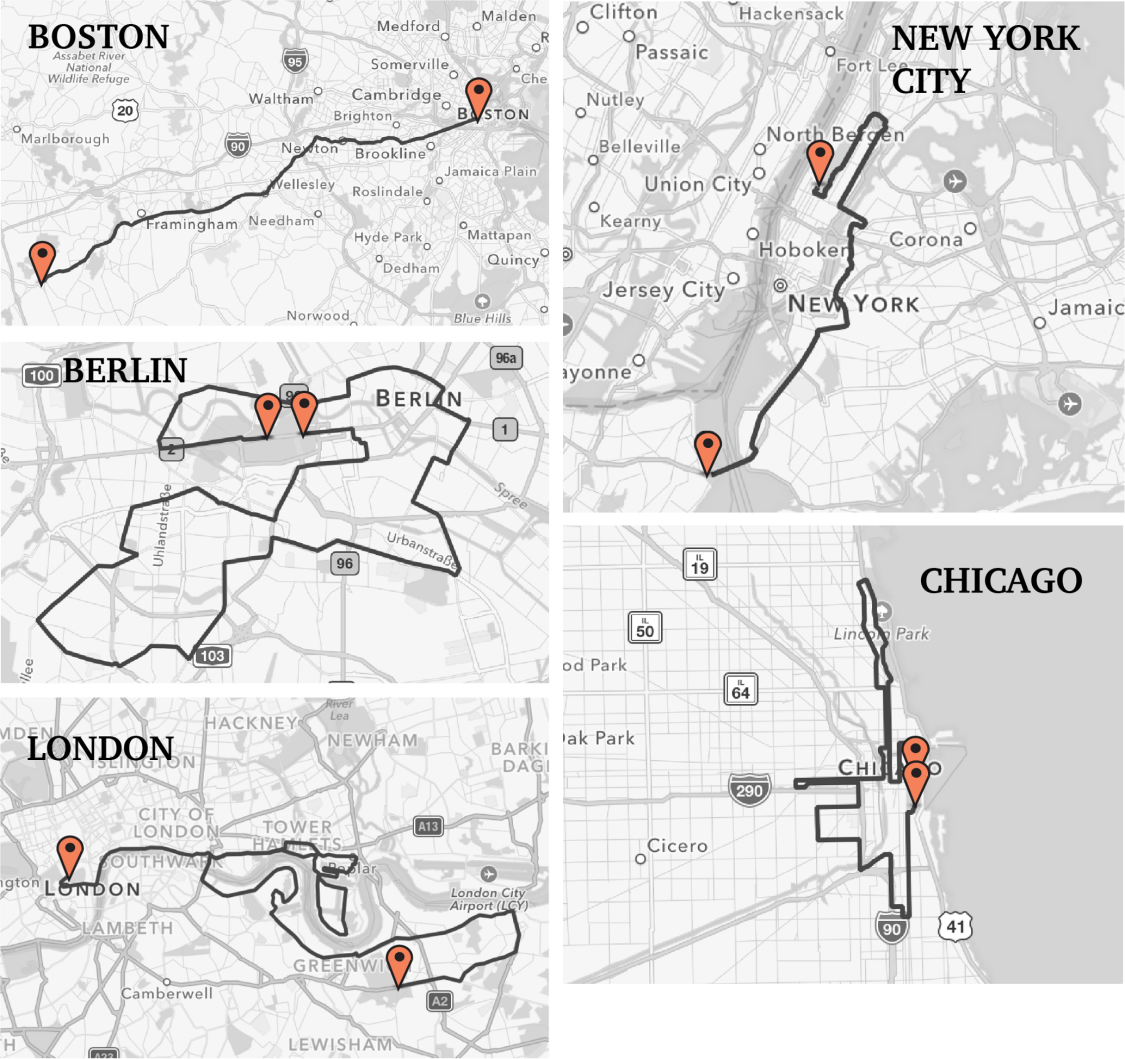
Course maps of Boston, New York City, Berlin, Chicago and London Marathons.

In regards specifically to the Boston Marathon course, the data show that its average downhill slope plays little role in making it “fast”, corroborating the anecdotal observations of many runners. Instead, the fact that it runs on one prevailing heading is what makes it susceptible to generating outliers of better performance when a prevailing wind aligns with race heading (Fig 4).

We also showed that performance times of the five world major marathons improved from 2005 to 2014, with times decreasing at a typical rate of ~1% per year for both genders. The Berlin and London marathons, by virtue of their traditionally faster race times and more frequent world record times, as opposed to Boston, appear to be advantageous for improved performances.

## Supporting information

S1 File Editorial-The Boston Marathon: Time to be record-eligible. (DOCX)

S2 File Data underlying results, analysis, and figures. (ZIP)
