# Supplementary material for: The Boston Marathon versus the World Marathon Majors
Source: PLoS One. 2017 Sep 1;12(9):e0184024. doi: 10.1371/journal.pone.0184024 (PMC5581174; doi:10.1371/journal.pone.0184024)
Supplement: S1 File — (DOCX) [file pone.0184024.s001.docx]

**Editorial**

The Boston Marathon: time to be record-eligible

Philip B. Maffetone^1^ and Paul B. Laursen^2^

^1^Athletic consultant, Arizona, USA.

^2^Sports Performance Research Institute New Zealand (SPRINZ), AUT University, Auckland, New Zealand

Corresponding Author:

Paul B. Laursen

Sports Performance Research Institute New Zealand (SPRINZ)

AUT University

Auckland, New Zealand

E-mail: paul.laursen@aut.ac.nz

A distinct feature of all sports is that each of their many venues are unique. Whether its baseball, soccer, football and even the indoor arenas, like hockey, basketball or even track and field, these theaters of sport all possess distinct intricacies, which are often glorified in the media. Marathon venues are no different. A marathon is run on roads, and each race is particularly distinctive—from World Championships to the Olympics, especially the time-tested annual big-city-street events of New York, Chicago, Berlin, London, and the most storied, Boston.

All sport venues throughout the world share a common feature—their unique physical characteristics and weather influence. While measured boundaries and distances are requirements of each sport, surface materials, spectators, atmospheric conditions and other factors vary, and influence performance, resulting in advantages and disadvantages of given venues. However, these different venues are not without restriction to their particular sports’ performance records. For example, no professional baseball park, football stadium or basketball arena excludes performance factors such as home runs, touchdowns or wins due to any perceived or real advantage. Among the many examples we could use is the difference between the professional baseball stadiums in Boston and Chicago. Data compiled by ESPN Sports (1) shows that Fenway Park (Boston) produces about 30% more runs per game compared to Wrigley Field (Chicago), 1.199 versus 0.874, yet all runs are counted and are record-eligible, despite the former venue favoring hitters and the latter arena harboring an advantage for pitchers. The same professional standard however is not followed in marathons, in particular the case with the most historical race in history, which is run directly past Fenway Park, the Boston Marathon.

The oldest annual event of its kind in the world, the Boston Marathon’s inaugural race, occurred in 1897. Virtually all marathon courses inevitably display differences in physical features except their certified distances of 42.195 kilometres (26.2 miles). Some ­are flatter or have more hills, others have more or less numbers of turns, and some are loops or are unidirectional. All these factors can influence finish times. Marathon performances are particularly influenced by temperature, humidity and wind. On any given race day, ideal weather conditions could lead to world record times on any big-city course. Indeed, our data, reported within the present issue of our journal shows this inevitable outcome, along with the progressive improvements in marathon times arising at about 1% per 7 years on average (2).

The Boston Marathon’s overall decrease in elevation from start to finish exceeds the International Amateur Athletic Federation’s (IAAF) limit for courses deemed world-record eligible. The elevation change infers there is an excessive amount of downhill running and therefore an unfair advantage over other courses for faster finish times. In addition, Boston’s unidirectional route could result in a southwest tailwind for runners, potentially improving performance, and another reason the event is ruled to be not record-eligible. When the occasional world-best Boston time is run by an athlete, it is not granted to the runner, which not only voids the personal recognition, but potentially affects his or her livelihood, as bonus, contract and other sponsorship income may be lost.

The IAAF’s ruling would appear to be subjective, and not scientifically based, as race times across the World Marathon Majors (WMM) courses have until now, not been adequately evaluated. Our recent study presented herein (2) compared finish times across the most popular marathon venues, so as to identify the faster and slower races in both genders. For this, we analyzed race times of the top 10 male and 10 female finishers over recent 10 years in Boston, London, Berlin, Chicago and New York Marathons.

The WMM began in 2005 as a championship series that includes the largest and most renowned marathons in the cities of Berlin, Boston, Chicago, London, New York and Tokyo. The WMM organization claims to be united in their efforts to advance the sport and its elite athletes, offering a $500,000 performance prize to the top male and females each year.

The IAAF, the world governing body for track and field athletics, including the marathon, has developed rules regarding road racing, deeming certain marathon courses not eligible for national and world record based on certain course features. These include elevation changes, specifically too much downhill running, and start-finish locations with the possibility that a point-to-point course may allow a tailwind. These two features tarnish Boston.

With years of finish-time data, the IAAF appears to have arbitrarily set rules regarding elevation and point-to-point being associated with a performance advantage, rather than use existing scientific data. This has led athletes, coaches, scientists and others in the sports community, who have known for years that the Boston Marathon is a relatively slow course due to the uphill segments and the steep downhill grades, to refer to these rules as flawed.

The results of our study side with the pundits, and do not support the IAAF’s rules regarding the Boston Marathon having an unfair advantage. We demonstrate that external factors such as weather and not course elevation can influence race times on the Boston course, and that the only WMM world-record finish, 2011 for men, was an outlier year, meaning that the record performances on that day were a rare occurrence outside the realm of probability. In fact, the all-time 50 fastest WMM times for men and women have been performed in Berlin, London and Chicago, with the exception of one year in Boston (2011) for men and one year in Boston (2014) for women. The Berlin and London marathons, in particular, produce the fastest finish times and most world records, giving these two venues an obvious advantage for faster performances. Our results show that the Boston course had a higher variability, and that race times are influenced to a larger degree by external factors such as weather, including wind conditions, rather than elevation. Excluding the weather factors, the relationship between course elevations and finish times does not indicate that the Boston course poses an unfair advantage.

In light of the evidence from our study, the best marathon performances and world record times, and criteria used across other sporting venues, it appears that the IAAF’s ruling in the case of the Boston Marathon’s record eligibility is inappropriate, particularly that the point-to-point nature and elevation drop in the Boston course is mitigated by weather events, not unlike situations in all other WMM events. As a result of this analysis, we feel that the IAAF should consider raising the quality of standards for the sport of marathon running to match those used in other professional athletics. It is time for the Boston venue to be ratified as record eligible.

1. <http://www.espn.com/mlb/stats/parkfactor>

2. Maffetone PB, Malcata R, Rivera I, Laursen PB. The Boston Marathon: Should it be record-eligible? (submitted)
