# Supplementary material for: The Boston Marathon versus the World Marathon Majors
Source: PLoS One. 2017 Sep 1;12(9):e0184024. doi: 10.1371/journal.pone.0184024 (PMC5581174; doi:10.1371/journal.pone.0184024)
Supplement: S2 File — (ZIP) [file pone.0184024.s002.zip › Boston Marathon/boston marathon methods and results.docx]

**Question**:

Is the boston Marathon a slower race?

**Methods**

Marathon performance times were analysed using the high-performance mixed linear model procedure (Proc Hpmixed) in the Statistical Analysis System (Versio 9.4, SAS Institute, Cary, NC). The fixed-model included a linear trend for calendar year, accounting for a general improvement of performance as result of better training and technology, and intercepts for the different race venues, providing mean marathon performance times for each of the venues: Berlin, Boston, Chicago, London, New York and Tokyo. The random effects in the model were Athlete, Raceid and BostonRace. The athlete effect estimates athletes’ running ability while addressing the repeated measurements structure of the data. Performances in each race (e.g., Boston 2014) were clustered by a RaceId random effect to account for mean effect of environmental factors on performance times of the day of each race. The BostonRace random effect was included in the model to allow for extra variance for the Boston performances. Performance times were log-transformed to yield the effects and errors in percent change of the mean.

Venue to venue comparison as well as comparison between Boston and all the others were calculated and expressed as a percent factor. Magnitudes for the difference in mean performance times were assed using a modified scale for standardized difference in means: thresholds for small, moderate, large, very large and extremely large were 0.2, 0.6, 1.2, 2.0 and 4.0 of the typical race-to-race variation. This typical race-to-race variation was determined as the standard deviation given bt the random effect for RaceId. Uncertainty on the estimates of performance and predicted mean times were derived as 90% confidence limits (1).

Analysis were performed including performances for all the six key marathons and a separately analysis was performed excluding performances at the Tokyo venue, due to the fact that it did not become part of the World Marathon Majors until 2007.

The effects of temperature, humidity and altitude gain, lost and change were investigated. The magnitude of effects of these factors was estimated, but the main focus of the analysis was to understand if these factors would further explain the performances. In particular, we were interested to see if the inclusion of such factors would explain some of the variability between races. If so, we would expect a reduction of the variability between races.

The model used for this investigation was similar to the one described above. The additional effects were included as fixed effect: Venue*Temperature and Venue*Humidity (providing standardize weather conditions for each venue), investigating the impact of the change in these weather conditions and altitude gain and altitude loss. Random effects were specified as in the model described above. Venue was removed from the model after an investigation of it as fixed or random effect proved over specification. Temperature and humidity information were collected from the Wunderground website and the altitude gain and loss were obtained from the official profiles.

**Results**

And I would include the following table, as it shows the comparison between boston and other races, with the magnitude of the effects**.**

Without Tokyo in the data set

|  | **Effect** | | **Effect** | **Lower** | **Upper** | **DF** | **Inference** |
| --- | --- | --- | --- | --- | --- | --- | --- |
| ***Women*** | |  |  |  |  |  |  |
|  | 7-year mean improvement in performance time* | | -1.0% | -2.2% | -0.1% | 484 | Small Positive |
|  | Boston vs… | |  |  |  |  |  |
|  |  | ALL OTHERS | 1.6% | 0.4% | 2.8% | 484 | Moderate Positive |
|  |  | BER | 1.5% | 0.0% | 3.0% | 484 | Moderate Positive |
|  |  | CHI | 1.3% | -0.2% | 2.8% | 484 | Moderate Positive |
|  |  | LON | 3.1% | 1.6% | 4.7% | 484 | Large Positive |
|  |  | NYC | 0.3% | -1.2% | 1.8% | 484 | Unclear |
| ***Men*** | |  |  |  |  |  |  |
|  | 7-year mean improvement in performance time* | | -1.1% | -1.9% | -0.2% | 484 | Moderate Positive |
|  | Boston vs… | |  |  |  |  |  |
|  |  | ALL OTHERS | 1.3% | 0.4% | 2.2% | 484 | Moderate Positive |
|  |  | BER | 1.6% | 0.4% | 2.7% | 484 | Moderate Positive |
|  |  | CHI | 1.3% | 0.1% | 2.4% | 484 | Moderate Positive |
|  |  | LON | 2.3% | 1.1% | 3.5% | 484 | Large Positive |
|  |  | NYC | 0.1% | -1.1% | 1.3% | 484 | Unclear |
| *The improvement reflects a decrease on performance times represented by a negative slope, for simplicity estimates presented here are the positive value of such slopes. | | | | | | | |

Study of variability Without Tokyo

|  |  | **Simple Model** | | **Include Temperature, Humidity, Altitude Gain and Loss factors** | |
| --- | --- | --- | --- | --- | --- |
|  |  | mean | 90% CL | mean | 90% CL |
| ***Women*** |  |  |  |  |  |
| Typical race-to-race variability | | 1.6% | 1.3-2.1% | 1.6% | 1.2-2.2% |
| Boston race-to-race variability | | 2.8% | 2.0-6.4% | 2.2% | 1.5-13.5% |
| ***Men*** |  |  |  |  |  |
| Typical race to race variability | | 1.3% | 1.1-1.7% | 1.1% | 0.7-2.2% |
| Boston race-to-race variability | | 1.9% | 1.3-6.5% | 1.8% | 1.2 -5.8% |

**Important points:**

- Boston venue is on average slower than other venues.
- Boston has a higher race-to-race variability than the other races. The higher variability, means that performances at Boston venue are less reliable.
- Inclusion of extra information about the venue and the weather conditions of the day of the race, explained slightly the variability shown on performances from race to race. The reduction of the variability was trivial, therefore it suggests that there must be other factors that have a greater impact in the variability of performances, e.g. wind conditions, use of pacers, motivation of athletes.
- I would choose not to include Tokyo races. Women performances were unusually slower in the first years, potentially due to the quality of the athletes and there is a perfect reason to exclude these races. They were not part of the circuit, therefore they were as competitive as the other races.

**Results: with Tokyo**

I would suggest a two graphs with the estimated means (with upper/lower limits) for each venue, something along these lines (data for this graph in excel).

|  | **Effect** | | **Effect** | **Lower** | **Upper** | **DF** | **Inference** |
| --- | --- | --- | --- | --- | --- | --- | --- |
| ***Women*** | |  |  |  |  |  |  |
|  | Boston vs… | |  |  |  |  |  |
|  |  | ALL OTHERS | 0.6% | -1.0% | 2.2% | 563 | Unclear |
|  |  | BER, CHI, LON, NYC | 1.6% | -0.1% | 3.3% | 563 | Small Positive |
|  |  | BER | 1.6% | -0.5% | 3.6% | 563 | Small Positive |
|  |  | CHI | 1.3% | -0.8% | 3.4% | 563 | Unclear |
|  |  | LON | 3.1% | 1.0% | 5.3% | 563 | Small Positive |
|  |  | NYC | 0.4% | -1.7% | 2.5% | 563 | Unclear |
|  |  | TOK | -3.2% | -5.4% | -1.1% | 563 | Moderate Negative |
| ***Men*** | |  |  |  |  |  |  |
|  | Boston vs… | |  |  |  |  |  |
|  |  | ALL OTHERS | 1.2% | 0.3% | 2.1% | 563 | Small Positive |
|  |  | BER, CHI, LON, NYC | 1.3% | 0.4% | 2.3% | 563 | Small Positive |
|  |  | BER | 1.6% | 0.5% | 2.7% | 563 | Small Positive |
|  |  | CHI | 1.3% | 0.1% | 2.4% | 563 | Small Positive |
|  |  | LON | 2.4% | 1.2% | 3.5% | 563 | Moderate Negative |
|  |  | NYC | 0.1% | -1.0% | 1.3% | 563 | Unclear |
|  |  | TOK | 0.7% | -0.5% | 2.0% | 563 | Unclear |
| *The improvement reflects a decrease on performance times represented by a negative slope, for simplicity estimates presented here are the positive value of such slopes. | | | | | | | |

| 7-year mean improvement in performance time* | | | |  |  |  |
| --- | --- | --- | --- | --- | --- | --- |
|  | Women | -2.5% | -4.4% | -0.9% | 563 | Moderate Positive |
|  | Men | -1.5% | -2.3% | -0.6% | 563 | Small Positive |

Study of variability Tokyo

|  |  | **Simple Model** | | **Include Temperature, Humidity, Altitude Gain and Loss factors** | |
| --- | --- | --- | --- | --- | --- |
|  |  | mean | 90% CL | mean | 90% CL |
| ***Women*** |  |  |  |  |  |
| Typical race-to-race variability | | 2.70% | 1.3-2.1% | 2.8% | 1.2-2.2% |
| Boston race-to-race variability | |  |  |  |  |
| ***Men*** |  |  |  |  |  |
| Typical race to race variability | | 1.30% | 1.1-1.7% | 1.0% | 0.8-1.4% |
| Boston race-to-race variability | | 1.90% | 1.3-6.7% | 1.8% | 1.2 -5.0% |
