# Supplementary material for: The Boston Marathon versus the World Marathon Majors
Source: PLoS One. 2017 Sep 1;12(9):e0184024. doi: 10.1371/journal.pone.0184024 (PMC5581174; doi:10.1371/journal.pone.0184024)
Supplement: S2 File — (ZIP) [file pone.0184024.s002.zip › Boston Marathon/listing.docx]

**Model 1**

class Gender Race Year0 runner;

Model Log_time_s= Year0/ noint s cl alpha=0.1;

random int /subject=Runner s cl alpha=0.1;

random Race/ s cl alpha=0.1;

Output out=predmean pred=Pred stderr=StdErr resid=Resid student=StudentResid LCL=Lower UCL=Upper

Mean times for the year:

Obs GENDER Year0 Estimate StdErr DF Lower Upper

1 F -11 2:30:43 0.8720 560 2:28:34 2:32:54

2 F -10 2:29:31 0.8679 560 2:27:24 2:31:41

3 F -9 2:34:59 0.8488 560 2:32:49 2:37:10

4 F -8 2:33:13 0.8510 560 2:31:05 2:35:23

5 F -7 2:32:05 0.8457 560 2:29:58 2:34:13

6 F -6 2:30:51 0.8485 560 2:28:46 2:32:59

7 F -5 2:27:59 0.8497 560 2:25:56 2:30:04

8 F -4 2:30:03 0.8742 560 2:27:54 2:32:14

9 F -3 2:30:00 0.8526 560 2:27:55 2:32:08

10 F -2 2:28:14 0.8567 560 2:26:09 2:30:20

11 M -11 2:11:02 0.4783 560 2:10:00 2:12:04

12 M -10 2:11:15 0.4746 560 2:10:14 2:12:17

13 M -9 2:13:30 0.4560 560 2:12:30 2:14:30

14 M -8 2:11:47 0.4532 560 2:10:48 2:12:46

15 M -7 2:11:02 0.4529 560 2:10:03 2:12:01

16 M -6 2:11:00 0.4528 560 2:10:02 2:11:59

17 M -5 2:09:41 0.4564 560 2:08:42 2:10:39

18 M -4 2:10:10 0.4717 560 2:09:09 2:11:11

19 M -3 2:10:03 0.4583 560 2:09:04 2:11:02

20 M -2 2:09:46 0.4574 560 2:08:48 2:10:45

Percent of time faster (negative percent) or slower (positive percent) than the average of each of the races: StdErr

Obs GENDER RACE Estimate Pred DF Lower Upper

314 F BER ( 0.8%) 0.7963 560 ( 2.1%) 0.5%

315 F BOS 0.6% 0.7903 560 ( 0.7%) 1.9%

316 F CHI ( 0.6%) 0.7934 560 ( 1.9%) 0.7%

317 F LON ( 2.3%) 0.7901 560 ( 3.6%) ( 1.0%)

318 F NYC 0.3% 0.7962 560 ( 1.0%) 1.6%

319 F TOK 2.9% 0.8235 560 1.6% 4.4%

636 M BER ( 0.5%) 0.4071 560 ( 1.2%) 0.1%

637 M BOS 1.0% 0.4054 560 0.3% 1.6%

638 M CHI ( 0.2%) 0.4052 560 ( 0.9%) 0.5%

639 M LON ( 1.2%) 0.4067 560 ( 1.8%) ( 0.5%)

640 M NYC 0.9% 0.4068 560 0.2% 1.5%

641 M TOK 0.1% 0.4236 560 ( 0.6%) 0.8%

Cov parms

Obs GENDER CovParm Subject Estimate

1 F Intercept RUNNER 9.1080

2 F RACE 3.2084

3 F Residual 5.9570

4 M Intercept RUNNER 2.0639

5 M RACE 0.7337

6 M Residual 3.4694

**Model2** allowing for a better or worse years for each athlete

This model decreased slightly the residuals compared to model1. But increased the covparm for race in men (compare 0.7337 model 1 with 0.7391 model2).

proc hpmixed data=marathon;

class Gender Race Year0 runner;

Model Log_time_s= Year0/ noint s cl alpha=0.1;

random int Year0/subject=Runner s cl alpha=0.1;

random Race/ s cl alpha=0.1;

Mean times for each year

Obs GENDER Year0 Estimate StdErr DF Lower Upper

1 F -11 2:30:44 0.8717 560 2:28:35 2:32:54

2 F -10 2:29:32 0.8684 560 2:27:25 2:31:42

3 F -9 2:35:00 0.8485 560 2:32:51 2:37:11

4 F -8 2:33:14 0.8506 560 2:31:06 2:35:24

5 F -7 2:32:04 0.8455 560 2:29:58 2:34:12

6 F -6 2:30:52 0.8478 560 2:28:46 2:32:59

7 F -5 2:27:58 0.8492 560 2:25:55 2:30:04

8 F -4 2:30:02 0.8740 560 2:27:54 2:32:13

9 F -3 2:30:00 0.8526 560 2:27:55 2:32:08

10 F -2 2:28:14 0.8566 560 2:26:09 2:30:20

11 M -11 2:11:02 0.4806 560 2:10:00 2:12:05

12 M -10 2:11:16 0.4769 560 2:10:14 2:12:18

13 M -9 2:13:31 0.4585 560 2:12:31 2:14:32

14 M -8 2:11:47 0.4552 560 2:10:48 2:12:47

15 M -7 2:11:02 0.4548 560 2:10:03 2:12:01

16 M -6 2:11:00 0.4552 560 2:10:02 2:12:00

17 M -5 2:09:42 0.4590 560 2:08:43 2:10:41

18 M -4 2:10:10 0.4738 560 2:09:09 2:11:11

19 M -3 2:10:02 0.4604 560 2:09:03 2:11:01

20 M -2 2:09:44 0.4597 560 2:08:46 2:10:44

Solution random for race

StdErr

Obs GENDER RACE Estimate Pred DF Lower Upper

3444 F BER ( 0.9%) 0.7946 560 ( 2.1%) 0.5%

3445 F BOS 0.6% 0.7884 560 ( 0.7%) 1.9%

3446 F CHI ( 0.6%) 0.7915 560 ( 1.9%) 0.7%

3447 F LON ( 2.3%) 0.7880 560 ( 3.6%) ( 1.0%)

3448 F NYC 0.3% 0.7943 560 ( 1.0%) 1.6%

3449 F TOK 2.9% 0.8220 560 1.6% 4.3%

6926 M BER ( 0.5%) 0.4082 560 ( 1.2%) 0.1%

6927 M BOS 1.0% 0.4060 560 0.3% 1.6%

6928 M CHI ( 0.2%) 0.4060 560 ( 0.9%) 0.5%

6929 M LON ( 1.2%) 0.4071 560 ( 1.8%) ( 0.5%)

6930 M NYC 0.9% 0.4074 560 0.2% 1.5%

6931 M TOK 0.1% 0.4246 560 ( 0.6%) 0.8%

Cov parm

Obs GENDER CovParm Subject Estimate

1 F Intercept RUNNER 9.0686

2 F Year0 RUNNER 0.3103

3 F RACE 3.1939

4 F Residual 5.6833

5 M Intercept RUNNER 2.0355

6 M Year0 RUNNER 0.2247

7 M RACE 0.7391

8 M Residual 3.2721

**Model3** including linear trend with year for mean times, reflecting the overall improvement in the standards of racing

Solution f

Obs GENDER Effect Estimate StdErr DF tValue Probt

1 F Intercept 909.16 0.8899 568 1021.68 <.0001

2 F yearX -0.2912 0.05947 568 -4.90 <.0001

3 M Intercept 895.52 0.4475 568 2001.34 <.0001

4 M yearX -0.2140 0.03873 568 -5.53 <.0001

Linear fitting

Obs GENDER Year0 Estimate StdErr DF Lower Upper

1 F -11 2:32:50 0.8473 568 2:30:43 2:34:59

2 F -10 2:32:23 0.8302 568 2:30:19 2:34:29

3 F -9 2:31:57 0.8171 568 2:29:55 2:34:00

4 F -8 2:31:30 0.8082 568 2:29:30 2:33:32

5 F -7 2:31:04 0.8036 568 2:29:04 2:33:04

6 F -6 2:30:37 0.8034 568 2:28:38 2:32:38

7 F -5 2:30:11 0.8076 568 2:28:12 2:32:12

8 F -4 2:29:45 0.8161 568 2:27:45 2:31:46

9 F -3 2:29:19 0.8287 568 2:27:17 2:31:22

10 F -2 2:28:53 0.8454 568 2:26:49 2:30:58

11 F -1 2:28:27 0.8659 568 2:26:20 2:30:35

12 F 0 2:28:01 0.8899 568 2:25:51 2:30:12

13 M -11 2:12:13 0.4136 568 2:11:19 2:13:07

14 M -10 2:11:56 0.3984 568 2:11:04 2:12:48

15 M -9 2:11:39 0.3865 568 2:10:49 2:12:29

16 M -8 2:11:22 0.3782 568 2:10:33 2:12:11

17 M -7 2:11:05 0.3737 568 2:10:17 2:11:54

18 M -6 2:10:48 0.3733 568 2:10:00 2:11:37

19 M -5 2:10:31 0.3768 568 2:09:43 2:11:20

20 M -4 2:10:15 0.3842 568 2:09:25 2:11:04

21 M -3 2:09:58 0.3953 568 2:09:07 2:10:49

22 M -2 2:09:41 0.4098 568 2:08:49 2:10:34

23 M -1 2:09:25 0.4273 568 2:08:30 2:10:20

24 M 0 2:09:08 0.4475 568 2:08:11 2:10:05

Percent difference from the average race

Obs GENDER RACE Estimate Pred DF Lower Upper

3444 F BER ( 0.9%) 0.8405 568 ( 2.3%) 0.4%

3445 F BOS 0.5% 0.8335 568 ( 0.9%) 1.9%

3446 F CHI ( 0.7%) 0.8367 568 ( 2.0%) 0.7%

3447 F LON ( 2.3%) 0.8324 568 ( 3.6%) ( 0.9%)

3448 F NYC 0.3% 0.8392 568 ( 1.1%) 1.7%

3449 F TOK 3.2% 0.8687 568 1.8% 4.7%

6926 M BER ( 0.5%) 0.4119 568 ( 1.2%) 0.1%

6927 M BOS 0.9% 0.4089 568 0.3% 1.6%

6928 M CHI ( 0.2%) 0.4091 568 ( 0.9%) 0.4%

6929 M LON ( 1.2%) 0.4097 568 ( 1.9%) ( 0.5%)

6930 M NYC 0.8% 0.4097 568 0.2% 1.5%

6931 M TOK 0.2% 0.4280 568 ( 0.5%) 0.9%

Covparms

Obs GENDER CovParm Subject Estimate

1 F Intercept RUNNER 9.5972

2 F Year0 RUNNER 1.5828

3 F RACE 3.5791

4 F Residual 5.5321

5 M Intercept RUNNER 2.1020

6 M Year0 RUNNER 0.5104

7 M RACE 0.7429

8 M Residual 3.2524

The simpler linear trend does not improve the fitting (not surprising).

**Model 4** linear year trend plus an intercept for each year

This model looks similar to Model 2

Solution f

Obs GENDER Effect Year0 Estimate StdErr DF

1 F Intercept _ 0 . .

2 F yearX _ 0 . .

3 F Year0 -9 910.98 0.8717 560

4 F Year0 -8 910.19 0.8684 560

5 F Year0 -7 913.77 0.8485 560

6 F Year0 -6 912.63 0.8506 560

7 F Year0 -5 911.87 0.8455 560

8 F Year0 -4 911.07 0.8478 560

9 F Year0 -3 909.14 0.8492 560

10 F Year0 -2 910.52 0.8740 560

11 F Year0 -1 910.50 0.8526 560

12 F Year0 0 909.31 0.8566 560

13 M Intercept _ 897.55 0.4552 560

14 M yearX _ 0 . .

15 M Year0 -9 -0.5706 0.4206 560

16 M Year0 -8 -0.3973 0.4179 560

17 M Year0 -7 1.3052 0.3884 560

18 M Year0 -6 0 . .

19 M Year0 -5 -0.5784 0.3834 560

20 M Year0 -4 -0.5918 0.3899 560

21 M Year0 -3 -1.5967 0.4005 560

22 M Year0 -2 -1.2382 0.4185 560

23 M Year0 -1 -1.3384 0.4104 560

24 M Year0 0 -1.5639 0.4085 560

Estimated means

Obs GENDER Year0 Estimate StdErr DF Lower Upper

1 F -9 2:30:44 0.8717 560 2:28:35 2:32:54

2 F -8 2:29:32 0.8684 560 2:27:25 2:31:42

3 F -7 2:35:00 0.8485 560 2:32:51 2:37:11

4 F -6 2:33:14 0.8506 560 2:31:06 2:35:24

5 F -5 2:32:04 0.8455 560 2:29:58 2:34:12

6 F -4 2:30:52 0.8478 560 2:28:46 2:32:59

7 F -3 2:27:58 0.8492 560 2:25:55 2:30:04

8 F -2 2:30:02 0.8740 560 2:27:54 2:32:13

9 F -1 2:30:00 0.8526 560 2:27:55 2:32:08

10 F 0 2:28:14 0.8566 560 2:26:09 2:30:20

11 M -9 2:11:02 0.4806 560 2:10:00 2:12:05

12 M -8 2:11:16 0.4769 560 2:10:14 2:12:18

13 M -7 2:13:31 0.4585 560 2:12:31 2:14:32

14 M -6 2:11:47 0.4552 560 2:10:48 2:12:47

15 M -5 2:11:02 0.4548 560 2:10:03 2:12:01

16 M -4 2:11:00 0.4552 560 2:10:02 2:12:00

17 M -3 2:09:42 0.4590 560 2:08:43 2:10:41

18 M -2 2:10:10 0.4738 560 2:09:09 2:11:11

19 M -1 2:10:02 0.4604 560 2:09:03 2:11:01

20 M 0 2:09:44 0.4597 560 2:08:46 2:10:44

Percent difference for each race

Obs GENDER RACE Estimate Pred DF Lower Upper

3444 F BER ( 0.9%) 0.7946 560 ( 2.1%) 0.5%

3445 F BOS 0.6% 0.7884 560 ( 0.7%) 1.9%

3446 F CHI ( 0.6%) 0.7915 560 ( 1.9%) 0.7%

3447 F LON ( 2.3%) 0.7880 560 ( 3.6%) ( 1.0%)

3448 F NYC 0.3% 0.7943 560 ( 1.0%) 1.6%

3449 F TOK 2.9% 0.8220 560 1.6% 4.3%

6926 M BER ( 0.5%) 0.4082 560 ( 1.2%) 0.1%

6927 M BOS 1.0% 0.4060 560 0.3% 1.6%

6928 M CHI ( 0.2%) 0.4060 560 ( 0.9%) 0.5%

6929 M LON ( 1.2%) 0.4071 560 ( 1.8%) ( 0.5%)

6930 M NYC 0.9% 0.4074 560 0.2% 1.5%

6931 M TOK 0.1% 0.4246 560 ( 0.6%) 0.8%

Covparms (raw as outputted)

Obs GENDER CovParm Subject Estimate

1 F Intercept RUNNER 9.0686

2 F Year0 RUNNER 0.3103

3 F RACE 3.1939

4 F Residual 5.6833

5 M Intercept RUNNER 2.0355

6 M Year0 RUNNER 0.2247

7 M RACE 0.7391

8 M Residual 3.2721

**Model 5 with a n intercept for each year and the race venue as a fixed effect**

proc hpmixed data=marathon;

class Gender Race Year0 runner;

Model Log_time_s= Year0 race/ noint s cl alpha=0.1;

random int Year0/subject=Runner s cl alpha=0.1;

Lsmeans race year0/ diff cl alpha=0.1;

Obs GENDER Effect RACE Year0 Estimate StdErr DF

1 F Year0 -9 -1.6314 0.5971 555

2 F Year0 -8 -2.4277 0.5835 555

3 F Year0 -7 1.1409 0.5641 555

4 F Year0 -6 0 . .

5 F Year0 -5 -0.7905 0.5634 555

6 F Year0 -4 -1.5821 0.5748 555

7 F Year0 -3 -3.5234 0.5850 555

8 F Year0 -2 -2.1363 0.6232 555

9 F Year0 -1 -2.1673 0.5899 555

10 F Year0 0 -3.3624 0.6027 555

11 F RACE BER _ 911.75 0.5310 555

12 F RACE BOS _ 913.24 0.5464 555

13 F RACE CHI _ 911.98 0.5346 555

14 F RACE LON _ 910.21 0.5394 555

15 F RACE NYC _ 912.92 0.5426 555

16 F RACE TOK _ 915.72 0.5810 555

17 M Year0 -9 896.77 0.3823 555

18 M Year0 -8 896.94 0.3789 555

19 M Year0 -7 898.65 0.3606 555

20 M Year0 -6 897.34 0.3569 555

21 M Year0 -5 896.76 0.3580 555

22 M Year0 -4 896.75 0.3559 555

23 M Year0 -3 895.74 0.3625 555

24 M Year0 -2 896.11 0.3781 555

25 M Year0 -1 896.00 0.3713 555

26 M Year0 0 895.77 0.3641 555

27 M RACE BER _ -0.3832 0.3312 555

28 M RACE BOS _ 1.2485 0.3159 555

29 M RACE CHI _ 0 . .

30 M RACE LON _ -1.0538 0.3280 555

31 M RACE NYC _ 1.1338 0.3326 555

32 M RACE TOK _ 0.3288 0.3619 555

Percentage difference form mean race (only for the men)

Obs GENDER RACE Estimate StdErr DF Lower Upper

1 M BER ( 0.4%) 0.3312 555 ( 0.9%) 0.2%

2 M BOS 1.3% 0.3159 555 0.7% 1.8%

3 M CHI 0.0% . . . .

4 M LON ( 1.0%) 0.3280 555 ( 1.6%) ( 0.5%)

5 M NYC 1.1% 0.3326 555 0.6% 1.7%

6 M TOK 0.3% 0.3619 555 ( 0.3%) 0.9%

Average Mean time for each race (lsmeans)

Obs GENDER RACE Estimate StdErr DF Lower Upper

1 F BER 2:29:25 0.3711 555 2:28:30 2:30:20

2 F BOS 2:31:39 0.3719 555 2:30:43 2:32:35

3 F CHI 2:29:45 0.3766 555 2:28:49 2:30:41

4 F LON 2:27:07 0.3749 555 2:26:13 2:28:02

5 F NYC 2:31:10 0.3996 555 2:30:10 2:32:10

6 F TOK 2:35:27 0.4286 555 2:34:22 2:36:34

7 M BER 2:10:09 0.2387 555 2:09:38 2:10:39

8 M BOS 2:12:17 0.2398 555 2:11:46 2:12:49

9 M CHI 2:10:39 0.2396 555 2:10:08 2:11:10

10 M LON 2:09:16 0.2538 555 2:08:44 2:09:49

11 M NYC 2:12:08 0.2554 555 2:11:35 2:12:41

12 M TOK 2:11:04 0.2767 555 2:10:29 2:11:40

Lsmeans diff, DF=555

Obs GENDER RACE _RACE Estimate StdErr Lower Upper

1 F BER BOS -1.4835 0.4821 -2.2778 -0.6892

2 F BER CHI -0.2264 0.4905 -1.0344 0.5817

3 F BER LON 1.5486 0.4804 0.7571 2.3401

4 F BER NYC -1.1665 0.5073 -2.0024 -0.3306

5 F BER TOK -3.9660 0.5469 -4.8672 -3.0649

6 F BOS CHI 1.2571 0.4753 0.4740 2.0402

7 F BOS LON 3.0321 0.4788 2.2432 3.8210

8 F BOS NYC 0.3170 0.4549 -0.4325 1.0664

9 F BOS TOK -2.4825 0.5512 -3.3907 -1.5744

10 F CHI LON 1.7750 0.4581 1.0202 2.5297

11 F CHI NYC -0.9402 0.4979 -1.7606 -0.1197

12 F CHI TOK -3.7397 0.5530 -4.6507 -2.8286

13 F LON NYC -2.7151 0.4666 -3.4839 -1.9464

14 F LON TOK -5.5146 0.5547 -6.4286 -4.6007

15 F NYC TOK -2.7995 0.5751 -3.7470 -1.8520

16 M BER BOS -1.6317 0.3309 -2.1769 -1.0866

17 M BER CHI -0.3832 0.3312 -0.9289 0.1625

18 M BER LON 0.6706 0.3324 0.1229 1.2182

19 M BER NYC -1.5171 0.3387 -2.0751 -0.9590

20 M BER TOK -0.7120 0.3562 -1.2988 -0.1251

21 M BOS CHI 1.2485 0.3159 0.7281 1.7689

22 M BOS LON 2.3023 0.3346 1.7510 2.8536

23 M BOS NYC 0.1147 0.3239 -0.4190 0.6484

24 M BOS TOK 0.9197 0.3644 0.3193 1.5202

25 M CHI LON 1.0538 0.3280 0.5133 1.5943

26 M CHI NYC -1.1338 0.3326 -1.6819 -0.5858

27 M CHI TOK -0.3288 0.3619 -0.9250 0.2675

28 M LON NYC -2.1876 0.3113 -2.7006 -1.6747

29 M LON TOK -1.3826 0.3717 -1.9950 -0.7701

30 M NYC TOK 0.8051 0.3741 0.1887 1.4215

**By combining averages using the spreadhset from Will Hopkins**

| **gender** | **mean** | **magnitude** | **qualifier** | **lower** | **upper** | **±** |
| --- | --- | --- | --- | --- | --- | --- |
| Using SD as 0:2:48 for F and 0:1:10 for M | | | | | | |
| F | 0:01:04 | 0.38 | trivial | 0:00:02 | 0:02:06 | 0:01:02 |
| M | 0:01:38 | 1.41 | moderate | 0:01:03 | 0:02:12 | 0:00:35 |
| Using SD as 0:3:43 for F and 0:2:18 for M | | | | | | |
| F | 0:01:04 | 0.29 | Trivial | 0:00:02 | 0:02:06 | 0:01:02 |
| M | 0:01:38 | 0.71 | small | 0:01:03 | 0:02:12 | 0:00:35 |

**Year means**

Obs GENDER Year0 Estimate StdErr DF Lower Upper

1 F -9 2:30:46 0.4773 555 2:29:35 2:31:57

2 F -8 2:29:34 0.4715 555 2:28:25 2:30:44

3 F -7 2:35:00 0.4335 555 2:33:54 2:36:07

4 F -6 2:33:14 0.4374 555 2:32:08 2:34:21

5 F -5 2:32:02 0.4276 555 2:30:58 2:33:07

6 F -4 2:30:50 0.4322 555 2:29:46 2:31:55

7 F -3 2:27:56 0.4349 555 2:26:53 2:29:00

8 F -2 2:30:00 0.4817 555 2:28:49 2:31:12

9 F -1 2:29:57 0.4414 555 2:28:52 2:31:03

10 F 0 2:28:10 0.4490 555 2:27:05 2:29:16

11 M -9 2:11:02 0.3286 555 2:10:20 2:11:45

12 M -8 2:11:16 0.3233 555 2:10:34 2:11:58

13 M -7 2:13:31 0.2953 555 2:12:52 2:14:10

14 M -6 2:11:47 0.2901 555 2:11:10 2:12:25

15 M -5 2:11:01 0.2894 555 2:10:24 2:11:39

16 M -4 2:11:00 0.2901 555 2:10:23 2:11:38

17 M -3 2:09:42 0.2961 555 2:09:04 2:10:20

18 M -2 2:10:10 0.3187 555 2:09:29 2:10:51

19 M -1 2:10:02 0.2981 555 2:09:23 2:10:40

20 M 0 2:09:44 0.2971 555 2:09:06 2:10:22

**covparms**

Obs GENDER CovParm Subject Estimate

1 F Intercept RUNNER 8.8706

2 F Year0 RUNNER 0.2806

3 F Residual 5.7874

4 M Intercept RUNNER 2.0274

5 M Year0 RUNNER 0.2409

6 M Residual 3.2619

Without 2011 boston race

Mean for each race

Obs GENDER RACE Estimate StdErr DF Lower Upper

1 F BER 2:29:24 0.3732 545 2:28:29 2:30:20

2 F BOS 2:32:00 0.3889 545 2:31:02 2:32:59

3 F CHI 2:29:46 0.3792 545 2:28:50 2:30:42

4 F LON 2:27:10 0.3767 545 2:26:16 2:28:05

5 F NYC 2:31:16 0.4024 545 2:30:16 2:32:17

6 F TOK 2:35:26 0.4313 545 2:34:20 2:36:32

7 M BER 2:10:09 0.2337 545 2:09:39 2:10:39

8 M BOS 2:12:54 0.2499 545 2:12:21 2:13:27

9 M CHI 2:10:36 0.2354 545 2:10:06 2:11:07

10 M LON 2:09:15 0.2499 545 2:08:43 2:09:47

11 M NYC 2:12:04 0.2516 545 2:11:31 2:12:37

12 M TOK 2:11:03 0.2706 545 2:10:28 2:11:38

**By combining averages using the spreadhset from Will Hopkins**

| **gender** | **mean** | **magnitude** | **qualifier** | **lower** | **upper** | ± |
| --- | --- | --- | --- | --- | --- | --- |
| Using SD as 0:2:48 for F and 0:1:19 for M | | | | | | |
| F | 0:01:24 | 0.50 | trivial | 0:00:20 | 0:02:28 | 0:01:04 |
| M | 0:02:17 | 1.73 | moderate | 0:01:41 | 0:02:53 | 0:00:36 |
| Using SD as 0:3:45 for F and 0:2:19 for M | | | | | | |
| F | 0:01:24 | 0.37 | trivial | 0:00:20 | 0:02:28 | 0:01:04 |
| M | 0:02:17 | 0.99 | small | 0:01:41 | 0:02:53 | 0:00:36 |

Mean time for each year

Obs GENDER Year0 Estimate StdErr DF Lower Upper

13 F -9 2:30:45 0.4786 545 2:29:34 2:31:57

14 F -8 2:29:34 0.4725 545 2:28:25 2:30:45

15 F -7 2:35:00 0.4347 545 2:33:53 2:36:07

16 F -6 2:33:13 0.4405 545 2:32:07 2:34:20

17 F -5 2:32:02 0.4302 545 2:30:58 2:33:07

18 F -4 2:30:52 0.4340 545 2:29:48 2:31:57

19 F -3 2:28:33 0.4767 545 2:27:24 2:29:44

20 F -2 2:30:05 0.4840 545 2:28:53 2:31:17

21 F -1 2:29:58 0.4439 545 2:28:53 2:31:05

22 F 0 2:28:15 0.4512 545 2:27:09 2:29:21

23 M -9 2:11:03 0.3193 545 2:10:21 2:11:44

24 M -8 2:11:17 0.3142 545 2:10:36 2:11:57

25 M -7 2:13:31 0.2864 545 2:12:53 2:14:09

26 M -6 2:11:49 0.2823 545 2:11:12 2:12:26

27 M -5 2:11:03 0.2818 545 2:10:26 2:11:39

28 M -4 2:11:00 0.2823 545 2:10:23 2:11:36

29 M -3 2:10:31 0.3114 545 2:09:51 2:11:12

30 M -2 2:10:07 0.3103 545 2:09:28 2:10:47

31 M -1 2:09:57 0.2902 545 2:09:20 2:10:35

32 M 0 2:09:43 0.2888 545 2:09:06 2:10:20

**Covparms**

Obs GENDER CovParm Subject Estimate

1 F Intercept RUNNER 9.1219

2 F Year0 RUNNER 0.2657

3 F Residual 5.7728

4 M Intercept RUNNER 1.9476

5 M Year0 RUNNER 0

6 M Residual 3.3358

Without 2011 boston race and Toyko race

Mean time for each race venue

Obs GENDER RACE Estimate StdErr DF Lower Upper

11 F BER 2:29:15 0.3299 466 2:28:27 2:30:04

12 F BOS 2:31:36 0.3460 466 2:30:44 2:32:28

13 F CHI 2:29:24 0.3355 466 2:28:34 2:30:13

14 F LON 2:26:44 0.3337 466 2:25:55 2:27:32

15 F NYC 2:31:01 0.3582 466 2:30:08 2:31:55

26 M BER 2:10:12 0.2452 466 2:09:41 2:10:44

27 M BOS 2:12:55 0.2601 466 2:12:21 2:13:30

28 M CHI 2:10:36 0.2455 466 2:10:05 2:11:08

29 M LON 2:09:18 0.2602 466 2:08:45 2:09:52

30 M NYC 2:12:05 0.2612 466 2:11:31 2:12:39

**By combining averages using the spreadhset from Will Hopkins**

| **gender** | **mean** | **magnitude** | **qualifier** | **lower** | **upper** | ± |
| --- | --- | --- | --- | --- | --- | --- |
| Using SD as 0:2:48 for F and 0:1:19 for M | | | | | | |
| F | 0:02:30 | 1.32 | moderate | 0:01:32 | 0:03:28 | 0:00:58 |
| M | 0:02:22 | 1.62 | moderate | 0:01:44 | 0:03:00 | 0:00:38 |
| Using SD as 0:3:45 for F and 0:2:19 for M | | | | | | |
| F | 0:02:30 | 0.67 | small | 0:01:32 | 0:03:28 | 0:00:58 |
| M | 0:02:22 | 0.96 | small | 0:01:44 | 0:03:00 | 0:00:38 |

Obs GENDER Year0 Estimate StdErr DF Lower Upper

1 F -9 2:29:29 0.4282 466 2:28:26 2:30:32

2 F -8 2:28:11 0.4262 466 2:27:09 2:29:14

3 F -7 2:32:36 0.4204 466 2:31:33 2:33:40

4 F -6 2:30:22 0.4271 466 2:29:19 2:31:26

5 F -5 2:31:28 0.4176 466 2:30:25 2:32:30

6 F -4 2:29:02 0.4229 466 2:28:00 2:30:05

7 F -3 2:27:36 0.4686 466 2:26:28 2:28:45

8 F -2 2:30:01 0.4819 466 2:28:50 2:31:13

9 F -1 2:29:33 0.4318 466 2:28:30 2:30:37

10 F 0 2:27:40 0.4356 466 2:26:37 2:28:44

16 M -9 2:11:01 0.3288 466 2:10:18 2:11:43

17 M -8 2:11:15 0.3234 466 2:10:33 2:11:57

18 M -7 2:13:14 0.3232 466 2:12:32 2:13:57

19 M -6 2:12:07 0.3175 466 2:11:25 2:12:48

20 M -5 2:10:47 0.3164 466 2:10:06 2:11:28

21 M -4 2:10:38 0.3178 466 2:09:57 2:11:19

22 M -3 2:10:35 0.3560 466 2:09:49 2:11:21

23 M -2 2:10:17 0.3581 466 2:09:31 2:11:03

24 M -1 2:10:14 0.3290 466 2:09:32 2:10:57

25 M 0 2:10:07 0.3262 466 2:09:25 2:10:49

**covparms**

Obs GENDER CovParm Subject Estimate

1 F Intercept RUNNER 5.6723

2 F Year0 RUNNER 0.1993

3 F Residual 5.1494

4 M Intercept RUNNER 2.3784

5 M Year0 RUNNER 0

6 M Residual 3.3803

**Model 6**

**This model has a linear year trend and race venue as fixed effects. The year trend accounts for the expected improvement of race performance due improvement in training, technology, the Race venue fixed effect will determine the mean time for each venue. Enabling comparison between venues. As random effects it was included athlete and athlete*year allow for repeated measures among athletes and recognizes that athletes will have good and bad years. Because the year to year variation was zero, this was further removed from the model. Furthermore, performances were clusters within races. This raceid clusters accounts for race to race variations, arising from variations in weather and other factors that affect all the athletes in the same way.**

proc hpmixed data=marathon;

class Gender RaceID Race Year0 runner;

Model Log_time_s= YearX race/ s cl alpha=0.1;

random int Year0/subject=Runner s cl alpha=0.1;

random RaceID/ s cl alpha=0.1;

Lsmeans race / diff cl alpha=0.1;

Solution fixed

GENDER Effect RACE Estimate StdErr DF Lower Upper

F Intercept 0 . . . .

F yearX -0.3522 0.1349 563 -0.5744 -0.1299

F RACE BER 908.30 1.0887 563 906.50 910.09

F RACE BOS 909.86 1.0870 563 908.07 911.65

F RACE CHI 908.59 1.0872 563 906.79 910.38

F RACE LON 906.77 1.0872 563 904.98 908.56

F RACE NYC 909.50 1.1493 563 907.60 911.39

F RACE TOK 913.16 1.1130 563 911.32 914.99

M Intercept 896.87 0.6300 563 895.83 897.90

M yearX -0.2102 0.07450 563 -0.3329 -0.08747

M RACE BER -1.4956 0.7140 563 -2.6720 -0.3192

M RACE BOS 0.1227 0.7085 563 -1.0447 1.2901

M RACE CHI -1.1467 0.7112 563 -2.3184 0.02497

M RACE LON -2.2072 0.7045 563 -3.3679 -1.0465

M RACE NYC 0 . . . .

M RACE TOK -0.6016 0.7654 563 -1.8627 0.6595

Lsmeans

GENDER RACE Estimate StdErr DF Lower Upper

F BER 2:29:02 0.9013 563 2:26:50 2:31:15

F BOS 2:31:23 0.9010 563 2:29:09 2:33:39

F CHI 2:29:28 0.9021 563 2:27:15 2:31:42

F LON 2:26:46 0.9017 563 2:24:36 2:28:58

F NYC 2:30:50 0.9521 563 2:28:29 2:33:13

F TOK 2:36:27 1.0152 563 2:33:51 2:39:05

M BER 2:10:08 0.4942 563 2:09:05 2:11:12

M BOS 2:12:16 0.4947 563 2:11:11 2:13:21

M CHI 2:10:36 0.4944 563 2:09:32 2:11:40

M LON 2:09:13 0.4994 563 2:08:10 2:10:17

M NYC 2:12:06 0.5217 563 2:10:58 2:13:15

M TOK 2:11:19 0.5589 563 2:10:06 2:12:31

Covparms

1 F Intercept RUNNER 4.7379

2 F Year0 RUNNER 0

3 F Raceid 7.3508

4 F Residual 3.2930

5 M Intercept RUNNER 1.7177

6 M Year0 RUNNER 0

7 M Raceid 2.0553

8 M Residual 2.0783

Covparms as percent

Obs GENDER CovParm Subject Estimate

1 F Intercept RUNNER 2.2%

2 F Year0 RUNNER 0.0%

3 F Raceid 2.7%

4 F Residual 1.8%

5 M Intercept RUNNER 1.3%

6 M Year0 RUNNER 0.0%

7 M Raceid 1.4%

8 M Residual 1.5%

Comparison between individual races

Obs GENDER RACE _RACE Estimate StdErr Lower Upper

1 F BER BOS ( 1.6%) 1.2651 ( 3.6%) 0.5%

2 F BER CHI ( 0.3%) 1.2668 ( 2.3%) 1.8%

3 F BER LON 1.5% 1.2651 ( 0.6%) 3.7%

4 F BER NYC ( 1.2%) 1.3023 ( 3.3%) 1.0%

5 F BER TOK ( 4.7%) 1.3546 ( 6.8%) ( 2.6%)

6 F BOS CHI 1.3% 1.2629 ( 0.8%) 3.4%

7 F BOS LON 3.1% 1.2639 1.0% 5.3%

8 F BOS NYC 0.4% 1.2914 ( 1.7%) 2.5%

9 F BOS TOK ( 3.2%) 1.3549 ( 5.4%) ( 1.1%)

10 F CHI LON 1.8% 1.2598 ( 0.3%) 4.0%

11 F CHI NYC ( 0.9%) 1.2993 ( 3.0%) 1.2%

12 F CHI TOK ( 4.5%) 1.3555 ( 6.6%) ( 2.3%)

13 F LON NYC ( 2.7%) 1.2934 ( 4.7%) ( 0.6%)

14 F LON TOK ( 6.2%) 1.3559 ( 8.3%) ( 4.1%)

15 F NYC TOK ( 3.6%) 1.3937 ( 5.8%) ( 1.4%)

16 M BER BOS ( 1.6%) 0.6961 ( 2.7%) ( 0.5%)

17 M BER CHI ( 0.3%) 0.6959 ( 1.5%) 0.8%

18 M BER LON 0.7% 0.6962 ( 0.4%) 1.9%

19 M BER NYC ( 1.5%) 0.7140 ( 2.6%) ( 0.3%)

20 M BER TOK ( 0.9%) 0.7432 ( 2.1%) 0.3%

21 M BOS CHI 1.3% 0.6905 0.1% 2.4%

22 M BOS LON 2.4% 0.6965 1.2% 3.5%

23 M BOS NYC 0.1% 0.7085 ( 1.0%) 1.3%

24 M BOS TOK 0.7% 0.7461 ( 0.5%) 2.0%

25 M CHI LON 1.1% 0.6943 ( 0.1%) 2.2%

26 M CHI NYC ( 1.1%) 0.7112 ( 2.3%) 0.0%

27 M CHI TOK ( 0.5%) 0.7452 ( 1.8%) 0.7%

28 M LON NYC ( 2.2%) 0.7045 ( 3.3%) ( 1.0%)

29 M LON TOK ( 1.6%) 0.7482 ( 2.8%) ( 0.4%)

30 M NYC TOK 0.6% 0.7654 ( 0.7%) 1.9%

Is Boston a slower race than the other?

GENDER Label Estimate StdErr DF Lower Upper

F Boston - BER 1.6% 1.2651 563 ( 0.5%) 3.7%

F Boston - Ber CHI 1.4% 1.0939 563 ( 0.4%) 3.3%

F Boston - BER CHI LON . . . . .

F Boston - BER CHI LON NYC 1.6% 0.9998 563 ( 0.1%) 3.3%

F Boston vs other 0.6% 0.9835 563 ( 1.0%) 2.2%

M Boston - BER 1.6% 0.6961 563 0.5% 2.8%

M Boston - Ber CHI 1.5% 0.5996 563 0.5% 2.5%

M Boston - BER CHI LON . . . . .

M Boston - BER CHI LON NYC 1.3% 0.5495 563 0.4% 2.3%

M Boston vs other 1.2% 0.5407 563 0.3% 2.1%

Without Tokyo

Is boston quicker or slower than the other races?

F year slope ( 0.2%) 0.1036 484 ( 0.3%) 0.0%

F Boston - BER 1.6% 0.9194 484 0.0% 3.1%

F Boston - Ber CHI 1.4% 0.7937 484 0.1% 2.8%

F Boston - BER CHI LON . . . . .

F Boston - BER CHI LON NYC 1.6% 0.7244 484 0.4% 2.8%

M year slope ( 0.2%) 0.07888 484 ( 0.3%) ( 0.0%)

M Boston - BER 1.6% 0.7000 484 0.4% 2.8%

M Boston - Ber CHI 1.4% 0.6020 484 0.4% 2.4%

M Boston - BER CHI LON . . . . .

M Boston - BER CHI LON NYC 1.3% 0.5515 484 0.4% 2.2%

**Covparms**

Obs GENDER CovParm Subject Estimate

1 F Intercept RUNNER 2.2%

2 F Raceid 1.9%

3 F Residual 1.8%

4 M Intercept RUNNER 1.5%

5 M Raceid 1.4%

6 M Residual 1.5%

Average time for the races

GENDER RACE Estimate StdErr DF Lower Upper

F BER 2:29:01 0.6605 484 2:27:25 2:30:39

F BOS 2:31:21 0.6596 484 2:29:43 2:33:00

F CHI 2:29:25 0.6612 484 2:27:48 2:31:03

F LON 2:26:47 0.6603 484 2:25:12 2:28:24

F NYC 2:30:55 0.6968 484 2:29:11 2:32:39

M BER 2:10:16 0.4981 484 2:09:12 2:11:21

M BOS 2:12:20 0.4976 484 2:11:15 2:13:25

M CHI 2:10:39 0.4975 484 2:09:35 2:11:44

M LON 2:09:20 0.5029 484 2:08:16 2:10:25

M NYC 2:12:11 0.5241 484 2:11:03 2:13:20

Lsmeans diff

Obs GENDER RACE _RACE Estimate StdErr Lower Upper

1 F BER BOS ( 1.5%) 0.9194 ( 3.0%) ( 0.0%)

2 F BER CHI ( 0.3%) 0.9223 ( 1.8%) 1.3%

3 F BER LON 1.5% 0.9196 ( 0.0%) 3.1%

4 F BER NYC ( 1.3%) 0.9480 ( 2.8%) 0.3%

5 F BOS CHI 1.3% 0.9165 ( 0.2%) 2.8%

6 F BOS LON 3.1% 0.9177 1.6% 4.7%

7 F BOS NYC 0.3% 0.9324 ( 1.2%) 1.8%

8 F CHI LON 1.8% 0.9118 0.3% 3.3%

9 F CHI NYC ( 1.0%) 0.9436 ( 2.5%) 0.6%

10 F LON NYC ( 2.7%) 0.9352 ( 4.2%) ( 1.2%)

11 M BER BOS ( 1.6%) 0.7000 ( 2.7%) ( 0.4%)

12 M BER CHI ( 0.3%) 0.7000 ( 1.4%) 0.9%

13 M BER LON 0.7% 0.6997 ( 0.4%) 1.9%

14 M BER NYC ( 1.5%) 0.7177 ( 2.6%) ( 0.3%)

15 M BOS CHI 1.3% 0.6927 0.1% 2.4%

16 M BOS LON 2.3% 0.6993 1.1% 3.5%

17 M BOS NYC 0.1% 0.7105 ( 1.1%) 1.3%

18 M CHI LON 1.0% 0.6971 ( 0.1%) 2.2%

19 M CHI NYC ( 1.2%) 0.7137 ( 2.3%) 0.0%

20 M LON NYC ( 2.2%) 0.7058 ( 3.3%) ( 1.0%)

Without Tokyo and 2011 Boston

Comparison between boston and other races

F year slope ( 0.1%) 0.1017 474 ( 0.3%) 0.0%

F Boston - BER 2.0% 0.9227 474 0.4% 3.5%

F Boston - Ber CHI 1.8% 0.8036 474 0.5% 3.2%

F Boston - BER CHI LON . . . . .

F Boston - BER CHI LON NYC 1.9% 0.7377 474 0.7% 3.2%

M year slo ( 0.2%) 0.07356 474 ( 0.3%) ( 0.0%)

M Boston - BE 2.0% 0.6673 474 0.9% 3.2%

M Boston - Ber CH 1.9% 0.5787 474 0.9% 2.9%

M Boston - BER CHI LO . . . . .

M Boston - BER CHI LON NYC 1.8% 0.5337 474 0.9% 2.7%

Covparms

Obs GENDER CovParm Subject Estimate

1 F Intercept RUNNER 2.2%

2 F Raceid 1.9%

3 F Residual 1.9%

4 M Intercept RUNNER 1.5%

5 M Raceid 1.3%

6 M Residual 1.5%

Mean times for races:

GENDER RACE Estimate StdErr DF Lower Upper

F BER 2:29:02 0.6466 474 2:27:27 2:30:38

F BOS 2:31:57 0.6785 474 2:30:15 2:33:39

F CHI 2:29:24 0.6477 474 2:27:49 2:31:00

F LON 2:26:49 0.6464 474 2:25:15 2:28:23

F NYC 2:30:58 0.6824 474 2:29:17 2:32:40

M BER 2:10:16 0.4626 474 2:09:16 2:11:16

M BOS 2:12:56 0.4867 474 2:11:52 2:14:00

M CHI 2:10:39 0.4622 474 2:09:39 2:11:39

M LON 2:09:20 0.4679 474 2:08:20 2:10:20

M NYC 2:12:11 0.4868 474 2:11:08 2:13:15

Just top 10

F year slope ( 1.1%) 0.7252 484 ( 2.3%) 0.1%

F Boston - BER 1.6% 0.9194 484 0.0% 3.1%

F Boston - Ber CHI 1.4% 0.7937 484 0.1% 2.8%

F Boston - BER CHI LON . . . . .

F Boston - BER CHI LON NYC 1.6% 0.7244 484 0.4% 2.8%

M year slope ( 1.1%) 0.5521 484 ( 2.0%) ( 0.2%)

M Boston - BER 1.6% 0.7000 484 0.4% 2.8%

M Boston - Ber CHI 1.4% 0.6020 484 0.4% 2.4%

M Boston - BER CHI LON . . . . .

M Boston - BER CHI LON NYC 1.3% 0.5515 484 0.4% 2.2%

Covparms

1 F Intercept RUNNER 2.2%

2 F Raceid 1.9%

3 F Residual 1.8%

4 M Intercept RUNNER 1.5%

5 M Raceid 1.4%

6 M Residual 1.5%

GENDER RACE Estimate StdErr DF Lower Upper

F BER 2:29:01 0.6605 484 2:27:25 2:30:39

F BOS 2:31:21 0.6596 484 2:29:43 2:33:00

F CHI 2:29:25 0.6612 484 2:27:48 2:31:03

F LON 2:26:47 0.6603 484 2:25:12 2:28:24

F NYC 2:30:55 0.6968 484 2:29:11 2:32:39

M BER 2:10:16 0.4981 484 2:09:12 2:11:21

M BOS 2:12:20 0.4976 484 2:11:15 2:13:25

M CHI 2:10:39 0.4975 484 2:09:35 2:11:44

M LON 2:09:20 0.5029 484 2:08:16 2:10:25

M NYC 2:12:11 0.5241 484 2:11:03 2:13:20

Model 7:

data clevmean predmean covmean solfmean solrmean estmean lsmeansmean diffmean;

proc mixed data=marathon covtest cl;

class Gender RaceID Race Year0 runner;

Model Log_time_s= YearX/ s outp=predmean residual cl alpha=0.1;

random int /subject=Runner s cl alpha=0.1;

random Year0/ group=Race s cl alpha=0.1;

*Output out=predmean pred=Pred stderr=StdErr resid=Resid student=StudentResid LCL=Lower UCL=Upper

Pred(noblup)=PredMean StdErr(noblup)=StdErrMean LCL(noblup)=LowerMean UCL(noblup)=UpperMean;

/*

estimate “2005” int 1 YearX -9 /cl alpha=0.1;

estimate “2006” int 1 YearX -8 /cl alpha=0.1;

estimate “2007” int 1 YearX -7 /cl alpha=0.1;

estimate “2008” int 1 YearX -6 /cl alpha=0.1;

estimate “2009” int 1 YearX -5 /cl alpha=0.1;

estimate “2010” int 1 YearX -4 /cl alpha=0.1;

estimate “2011” int 1 YearX -3 /cl alpha=0.1;

estimate “2012” int 1 YearX -2 /cl alpha=0.1;

estimate “2013” int 1 YearX -1 /cl alpha=0.1;

estimate “2014” int 1 YearX 0 /cl alpha=0.1;

Estimate “year slope” YearX 7/cl alpha=0.1;

Estimate “Boston - BER” Race -1 1/cl alpha=0.1;

Estimate “Boston – Ber CHI” Race -0.5 1 -0.5/cl alpha=0.1;

Estimate “Boston – BER CHI LON” Race -0.3 1 -0.3 -0.3/cl alpha=0.1;

Estimate “Boston – BER CHI LON NYC” Race -0.25 1 -0.25 -0.25 -0.25/cl alpha=0.1;

Estimate “Boston vs other” Race -0.2 1 -0.2 -0.2 -0.2 -0.2/cl alpha=0.1;

*/

ods output estimates=estmean;

ods output solutionr=solrmean;

ods output parameterestimates=solfmean;

ods output classlevels=clevmean;

ods output covparms=covmean;

ods output lsmeans=lsmeansmean;

ods output diffs=ldiffmean;

by gender;

run;

Obs GENDER CovParm Group Estimate StdErr ZValue Lower Upper

1 F Intercept 4.8600 0.7058 6.89 3.7267 6.6048

2 F Year0 RACE BER 1.4755 1.0062 1.47 0.5442 10.9111

3 F Year0 RACE BOS 8.5826 4.2026 2.04 3.9671 30.4089

4 F Year0 RACE CHI 5.6252 2.7306 2.06 2.6141 19.6474

5 F Year0 RACE LON 3.2140 2.2169 1.45 1.1754 24.5918

6 F Year0 RACE NYC 2.3761 1.5921 1.49 0.8878 16.7046

7 F Year0 RACE TOK 50.1928 25.8078 1.94 22.4985 193.48

8 F Residual 3.2428 0.3267 9.93 2.6869 3.9919

9 M Intercept 1.7729 0.3264 5.43 1.2738 2.6371

10 M Year0 RACE BER 1.3245 0.7509 1.76 0.5568 6.1770

11 M Year0 RACE BOS 5.5899 3.2701 1.71 2.3003 27.9056

12 M Year0 RACE CHI 3.7756 1.8873 2.00 1.7229 13.8508

13 M Year0 RACE LON 0.4685 1.1733 0.40 0.05655 1.3168E9

14 M Year0 RACE NYC 3.3019 2.4667 1.34 1.1365 32.7115

15 M Year0 RACE TOK 2.2506 1.7503 1.29 0.7507 25.8027

16 M Residual 2.0544 0.1998 10.28 1.7128 2.5100

Cov parms back transformed

Obs GENDER CovParm Group Estimate StdErr ZValue Lower Upper

1 F Intercept 2.2% 0.7058 6.89 1.9% 2.6%

2 F Year0 RACE BER 1.2% 1.0062 1.47 0.7% 3.4%

3 F Year0 RACE BOS 3.0% 4.2026 2.04 2.0% 5.7%

4 F Year0 RACE CHI 2.4% 2.7306 2.06 1.6% 4.5%

5 F Year0 RACE LON 1.8% 2.2169 1.45 1.1% 5.1%

6 F Year0 RACE NYC 1.6% 1.5921 1.49 0.9% 4.2%

7 F Year0 RACE TOK 7.3% 25.8078 1.94 4.9% 14.9%

8 F Residual 1.8% 0.3267 9.93 1.7% 2.0%

9 M Intercept 1.3% 0.3264 5.43 1.1% 1.6%

10 M Year0 RACE BER 1.2% 0.7509 1.76 0.7% 2.5%

11 M Year0 RACE BOS 2.4% 3.2701 1.71 1.5% 5.4%

12 M Year0 RACE CHI 2.0% 1.8873 2.00 1.3% 3.8%

13 M Year0 RACE LON 0.7% 1.1733 0.40 0.2% 4E159%

14 M Year0 RACE NYC 1.8% 2.4667 1.34 1.1% 5.9%

15 M Year0 RACE TOK 1.5% 1.7503 1.29 0.9% 5.2%

16 M Residual 1.4% 0.1998 10.28 1.3% 1.6%

Model 8:

proc mixed data=marathon covtest cl alpha=0.1;

class Gender RaceID Race Year0 runner;

Model Log_time_s= YearX race/ s outp=predmean residual cl alpha=0.1;

random int /subject=Runner s cl alpha=0.1;

random raceid raceid*varBos/ s cl alpha=0.1;

lsmeans race/diff alpha=0.1 cl;

*Output out=predmean pred=Pred stderr=StdErr resid=Resid student=StudentResid LCL=Lower UCL=Upper

Pred(noblup)=PredMean StdErr(noblup)=StdErrMean LCL(noblup)=LowerMean UCL(noblup)=UpperMean;

estimate "t_2005" int 1 YearX -9 /cl alpha=0.1;

estimate "t_2006" int 1 YearX -8 /cl alpha=0.1;

estimate "t_2007" int 1 YearX -7 /cl alpha=0.1;

estimate "t_2008" int 1 YearX -6 /cl alpha=0.1;

estimate "t_2009" int 1 YearX -5 /cl alpha=0.1;

estimate "t_2010" int 1 YearX -4 /cl alpha=0.1;

estimate "t_2011" int 1 YearX -3 /cl alpha=0.1;

estimate "t_2012" int 1 YearX -2 /cl alpha=0.1;

estimate "t_2013" int 1 YearX -1 /cl alpha=0.1;

estimate "t_2014" int 1 YearX 0 /cl alpha=0.1;

Estimate "year slope" YearX 7/cl alpha=0.1;

Estimate "Boston vs other" Race -0.2 1 -0.2 -0.2 -0.2 -0.2/cl alpha=0.1;

Estimate "Boston – BER CHI LON NYC" Race -0.25 1 -0.25 -0.25 -0.25/cl alpha=0.1;

Estimate "Boston - BER" Race -1 1/cl alpha=0.1;

Estimate "Boston - CHI" Race 0 1 -1/cl alpha=0.1;

Estimate "Boston - Lon" Race 0 1 0 -1/cl alpha=0.1;

Estimate "Boston - NYC" Race 0 1 0 0 -1/cl alpha=0.1;

Estimate "Boston - TKY" Race 0 1 0 0 0 -1/cl alpha=0.1;

Estimate "Boston – Ber CHI" Race -0.5 1 -0.5/cl alpha=0.1;

Estimate "Boston – BER CHI LON" Race -1 3 -1 -1/cl alpha=0.1 divisor=3;

estimate "t_Berlin" int 1 Race 1 /cl alpha=0.1;

estimate "t_Boston" Int 1 Race 0 1 /cl alpha=0.1;

estimate "t_Chicago" int 1 Race 0 0 1 /cl alpha=0.1;

estimate "t_London" int 1 Race 0 0 0 1 /cl alpha=0.1;

estimate "t_New York" int 1 Race 0 0 0 0 1 /cl alpha=0.1;

estimate "t_Tokyo" int 1 Race 0 0 0 0 0 1 /cl alpha=0.1;

ods output estimates=estmean;

ods output solutionr=solrmean;

ods output parameterestimates=solfmean;

ods output classlevels=clevmean;

ods output covparms=covmean;

ods output lsmeans=lsmeansmean;

ods output diffs=ldiffmean;

by gender;

run;

ods listing;

Cov with

| **Obs** | **GENDER** | **CovParm** | **Estimate** | **Lower** | **Upper** | **StdErr** | **ZValue** |
| --- | --- | --- | --- | --- | --- | --- | --- |
| **1** | F | Intercept | 2.2% | 2.0% | 2.5% | 0.7058 | 6.71 |
| **2** | F | Raceid | 2.7% | 2.3% | 3.4% | 1.7603 | 4.15 |
| **3** | F | varBOS*Raceid | 0.5% | . | . | 4.1896 | 0.07 |
| **4** | F | Residual | 1.8% | 1.7% | 2.0% | 0.3364 | 9.79 |
| **5** | M | Intercept | 1.3% | 1.2% | 1.5% | 0.2976 | 5.77 |
| **6** | M | Raceid | 1.3% | 1.1% | 1.7% | 0.4461 | 3.87 |
| **7** | M | varBOS*Raceid | 1.4% | 0.8% | 6.4% | 1.8897 | 0.99 |
| **8** | M | Residual | 1.5% | 1.3% | 1.6% | 0.1949 | 10.66 |

Including alt_change_google
