# Supplementary material for: The Boston Marathon versus the World Marathon Majors
Source: PLoS One. 2017 Sep 1;12(9):e0184024. doi: 10.1371/journal.pone.0184024 (PMC5581174; doi:10.1371/journal.pone.0184024)
Supplement: S2 File — (ZIP) [file pone.0184024.s002.zip › Boston Marathon/marathon times analysis for the local machine.docx]

*Read from the surver;

libname bb "\\tsclient\C\Users\RitaM\Dropbox\New Zealand\Collaborations 2\Boston Marathon”;

ods listing;

PROC IMPORT

DATAFILE="\\tsclient\C\Users\RitaM\Dropbox\New Zealand\Collaborations 2\Boston Marathon\boston_data.xls"

OUT=temp

DBMS=XLS replace;

GETNAMES=YES;

MIXED=NO;

attrib _character_ _numeric_ label="";

run;

data marathon;

set temp;

varBOS=0;

*if race=”BOS” and year=2011 then delete;

*if race=”TOK” then delete;

If race=”BOS” then VarBOS=1;

log_time_s=100*log(time_s);

log_time=100*log(time);

Year0=Year-2014;

yearX=Year0;

if place<11;

Raceid=Trim(Year||RAce);

rename Place=Rank;

drop Temp--X;

run;

proc print data=marathon;run;

proc sort data=marathon;

by gender Year year0 race Rank;

proc means data=marathon noprint;

var time_s;

by gender year year0 race;

output out=raw_race mean=mean stddev=sd;

run;

proc sort data=raw_race;

by Gender Race;

proc means data=raw_race noprint;

*class gender race;

var mean sd;

by gender race;

output out=variability mean=mean mean_sd stddev=sd;

run;

proc print data=variability;

var Gender Race mean sd;

format mean time8.1 sd time8.1;

run;

proc sort data=marathon;

by gender Year year0 race Rank;

proc means data=marathon noprint;

var time_s;

by gender year year0;

output out=raw_mean mean=mean stddev=sd;

run;

proc print data=raw_mean;run;

proc sort data=marathon;

by gender Year race Rank;

data clevmean predmean covmean solfmean solrmean estmean lsmeansmean diffmean;

proc mixed data=marathon covtest cl;

class Gender RaceID Race Year0 runner;

Model Log_time_s= YearX race/ s outp=predmean residual cl alpha=0.1;

random int /subject=Runner s cl alpha=0.1;

random raceid raceid*varBos/ s cl alpha=0.1;

*Output out=predmean pred=Pred stderr=StdErr resid=Resid student=StudentResid LCL=Lower UCL=Upper

Pred(noblup)=PredMean StdErr(noblup)=StdErrMean LCL(noblup)=LowerMean UCL(noblup)=UpperMean;

estimate “t_2005” int 1 YearX -9 /cl alpha=0.1;

estimate “t_2006” int 1 YearX -8 /cl alpha=0.1;

estimate “t_2007” int 1 YearX -7 /cl alpha=0.1;

estimate “t_2008” int 1 YearX -6 /cl alpha=0.1;

estimate “t_2009” int 1 YearX -5 /cl alpha=0.1;

estimate “t_2010” int 1 YearX -4 /cl alpha=0.1;

estimate “t_2011” int 1 YearX -3 /cl alpha=0.1;

estimate “t_2012” int 1 YearX -2 /cl alpha=0.1;

estimate “t_2013” int 1 YearX -1 /cl alpha=0.1;

estimate “t_2014” int 1 YearX 0 /cl alpha=0.1;

Estimate “year slope” YearX 7/cl alpha=0.1;

Estimate “Boston - BER” Race -1 1/cl alpha=0.1;

Estimate “Boston – Ber CHI” Race -0.5 1 -0.5/cl alpha=0.1;

Estimate “Boston – BER CHI LON” Race -0.3 1 -0.3 -0.3/cl alpha=0.1;

Estimate “Boston – BER CHI LON NYC” Race -0.25 1 -0.25 -0.25 -0.25/cl alpha=0.1;

Estimate “Boston vs other” Race -0.2 1 -0.2 -0.2 -0.2 -0.2/cl alpha=0.1;

estimate “t_Berlin” int 1 Race 1 /cl alpha=0.1;

estimate “t_Boston” Int 1 Race 0 1 /cl alpha=0.1;

estimate “t_Chicago” int 1 Race 0 0 1 /cl alpha=0.1;

estimate “t_London” int 1 Race 0 0 0 1 /cl alpha=0.1;

estimate “t_New York” int 1 Race 0 0 0 0 1 /cl alpha=0.1;

estimate “t_Tokyo” int 1 Race 0 0 0 0 0 1 /cl alpha=0.1;

ods output estimates=estmean;

ods output solutionr=solrmean;

ods output parameterestimates=solfmean;

ods output classlevels=clevmean;

ods output covparms=covmean;

ods output lsmeans=lsmeansmean;

ods output diffs=ldiffmean;

by gender;

run;

proc print data=clevmean;run;

proc print data=covmean;

*var GENDER CovParm Group Estimate StdErr ZValue Lower Upper;

run;

data covback;

set covmean;

array a estimate lower upper;

do over a;

a=exp(sqrt(a)/100)-1;

end;

run;

proc print data=covback;

*var GENDER CovParm Group Estimate StdErr ZValue Lower Upper;

format estimate percent8.1 lower percent8.1 upper percent8.1;

run;

/*

*this works but then I cannot separate the format;

data estmean1;

set estmean;

array a estimate lower upper;

if substr(label,2,2)=”t_” then do;

do over a;

a=exp(a/100);

end;

end;

else

do over a;

a=exp(a/100)-1;

end;

*format estimate percent8.1 lower percent8.1 upper percent8.1;

run;

*/

data estmeantime;

set estmean;

array a estimate lower upper;

if substr(label,1,2)=”t_”;

do over a;

a=exp(a/100);

end;

label=substr(label,3,10);

year=label+0;

format estimate lower upper time8.0;

run;

proc print data=estmeantime;run;

data estmean1;

set estmean;

array a estimate lower upper;

if substr(label,1,2)=”t_” then delete;

do over a;

a=exp(a/100)-1;

end;

format estimate percent8.1 lower percent8.1 upper percent8.1;

run;

proc print data=estmean1 noobs;

var gender label estimate Stderr DF lower upper;

run;

proc sort data=estmeantime;

by gender year;

proc sort data=raw_mean;

by gender year;

data estmean2;

merge estmeantime raw_mean (keep=Gender year Year0 mean sd);

by gender year;

run;

proc print data=estmean2;

var gender year year0 estimate Stderr DF lower upper;

run;

symbol1 c=red v=dot i=none;

symbol2 c=blue v=none i=join;

proc gplot data=estmean2;

plot (mean estimate)*Year0/overlay;

by Gender;

format mean time8.0 estimate 8.0;

run;

*/

proc print data=solfmean (nobs);

var Gender effect race estimate stderr df lower upper;

run;

/*

data solfmean2;

merge solfmean1 raw_mean (keep=Gender year Year0 mean sd);

by gender Year0;

run;

symbol1 c=red v=dot i=none;

symbol2 c=blue v=none i=join;

proc gplot data=solfmean2;

plot (mean estimate)*Year0/overlay;

by Gender;

format mean time8.0 estimate 8.0;

run;

*/

/*

proc print data=solrmean;

where effect=”RACE”;

run;

*/

data solrmean1;

set solrmean;

array a estimate lower upper;

do over a;

a=exp(a/100)-1;

end;

format estimate percent8.1 lower percent8.1 upper percent8.1;

run;

proc print data=solrmean1;

*var gender Race Estimate stdErrPred DF Lower Upper;

where effect=”Raceid”;

run;

symbol1 c=red v=dot i=join;

symbol2 c=red v=none i=join l=1;

symbol3 c=red v=none i=join l=1;

proc gplot data=solrmean1;

plot (estimate)*raceid/overlay;

by Gender;

where effect=”Raceid”;

run;

/*

proc print data=solrmean;

*var gender Race Estimate stdErrPred DF Lower Upper;

where effect=”Raceid”;

run;

proc print data=solfmean;

var Gender effect race year0 estimate stderr df ;

run;

*/

data solfmean1;

set solfmean;

array a estimate lower upper;

do over a;

a=exp(a/100)-1;

end;

format estimate percent8.1 lower percent8.1 upper percent8.1;

run;

proc print data=solfmean1;

var Gender race estimate stderr df LOWER UPPER;

run;

proc print data=covmean;

run;

data covmean1;

set covmean;

estimate=sqrt(estimate);

estimate=exp(estimate/100)-1;

format estimate percent8.1;

run;

proc print data=covmean1;run;

data lsmeansmean1;

set lsmeansmean;

array a estimate lower upper;

do over a;

a=exp(a/100);

end;

format estimate time8.0 lower time8.0 upper time8.0;

run;

proc print data=lsmeansmean1 noobs;

var gender race estimate stderr df lower upper;

run;

symbol1 c=red v=dot i=join;

symbol2 c=red v=none i=join l=1;

symbol3 c=red v=none i=join l=1;

proc gplot data=lsmeansmean1;

plot (estimate upper lower)*race/overlay;

by Gender;

where effect=”RACE”;

run;

data ldiffmean1;

set ldiffmean;

array a estimate lower upper;

do over a;

a=exp(a/100)-1;

end;

format estimate percent8.1 upper percent8.1 lower percent8.1;

run;

proc print data=ldiffmean1;

var gender race _Race estimate stderr lower upper;

run;

proc print data=solfmean;

var Gender effect race estimate stderr df lower upper;

run;

/*

data lsmeansmean1;

set lsmeansmean;

array a estimate lower upper;

do over a;

a=exp(a/100);

end;

format estimate time8.0 lower time8.0 upper time8.0;

run;

proc sort data=lsmeansmean1;

by gender;

title “Mean for each race”;

proc print data=lsmeansmean1;

var gender race estimate stderr df lower upper;

where effect=”RACE”;

run;

symbol1 c=red v=dot i=join;

symbol2 c=red v=none i=join l=1;

symbol3 c=red v=none i=join l=1;

proc gplot data=lsmeansmean1;

plot (estimate upper lower)*race/overlay;

by Gender;

where effect=”RACE”;

run;

title “Mean time for each year”;

proc print data=lsmeansmean1;

var gender year0 estimate stderr df lower upper;

where effect=”Year0”;

run;

data lsmeansmean2;

merge lsmeansmean1 raw_mean (keep=Gender year Year0 mean sd);

by gender Year0;

run;

symbol1 c=red v=dot i=none;

symbol2 c=blue v=none i=join;

proc gplot data=lsmeansmean2;

plot (mean estimate)*Year0/overlay;

by Gender;

format mean time8.0 estimate 8.0;

run;

proc sort data=raw_race;

by gender year0;

proc transpose data=raw_race out=raw_race_line;

var mean;

id race;

by gender year0;

run;

proc sort data=lsmeansmean1;

by gender year0;

data graph;

merge raw_race_line lsmeansmean1;

by gender year0;
format BER BOS CHI LON NYC TOK time8.0;

run;

proc print data=graph;

run;

symbol1 c=orange v=star i=none;

symbol2 c=red v=dot i=none;

symbol3 c=pink v=star i=none;

symbol4 c=cyan v=star i=none;

symbol5 c=purple v=star i=none;

symbol6 c=blue v=none i=join;

symbol7 c=blue v=none i=join l=2;

symbol8 c=blue v=none i=join l=2;

PROC gplot data=graph;

plot (BER BOS CHI LON NYC estimate upper lower)*year0/overlay legend;

By gender;

Run;

*/
