# Supplementary figures and images for: The Boston Marathon versus the World Marathon Majors
Source: PLoS One. 2017 Sep 1;12(9):e0184024. doi: 10.1371/journal.pone.0184024 (PMC5581174; doi:10.1371/journal.pone.0184024)

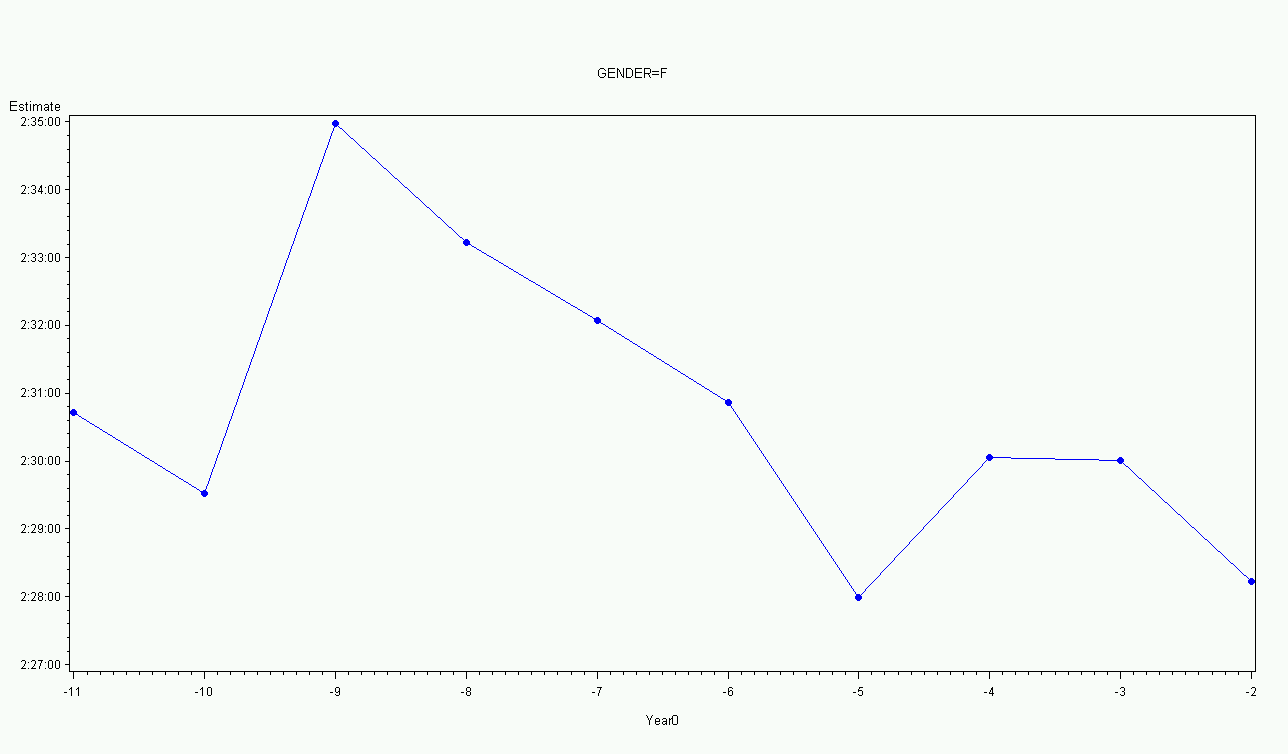

Supplement: S2 File — (ZIP) [file pone.0184024.s002.zip › Boston Marathon/Marathon W Model 1.bmp]

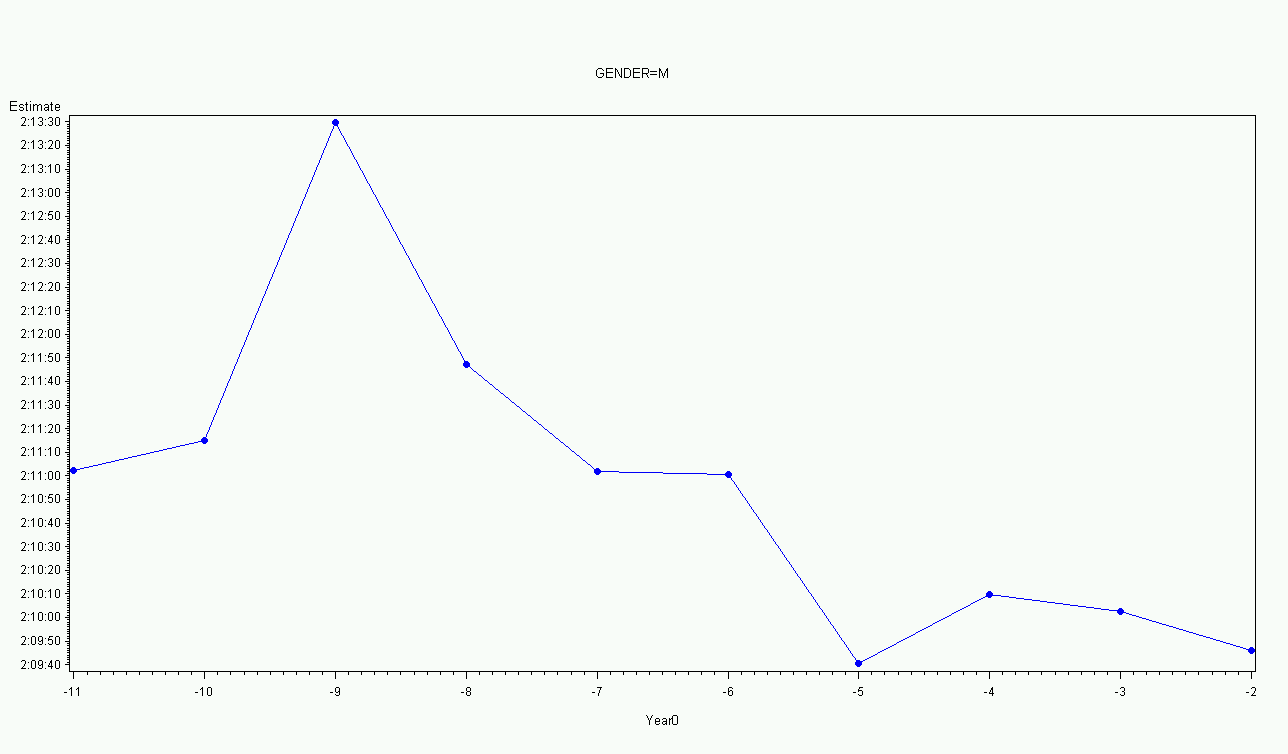

Supplement: S2 File — (ZIP) [file pone.0184024.s002.zip › Boston Marathon/Marrathon M Model 1.bmp]

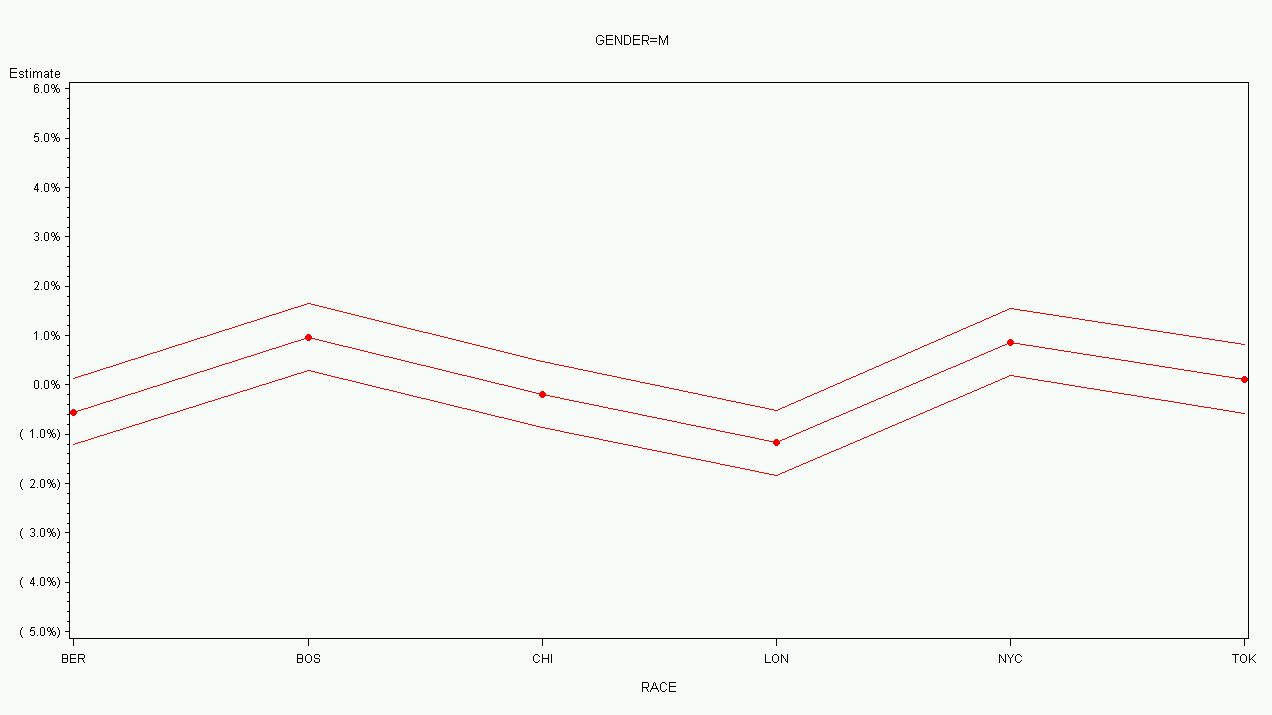

Supplement: S2 File — (ZIP) [file pone.0184024.s002.zip › Boston Marathon/Model 2 Race M.bmp]

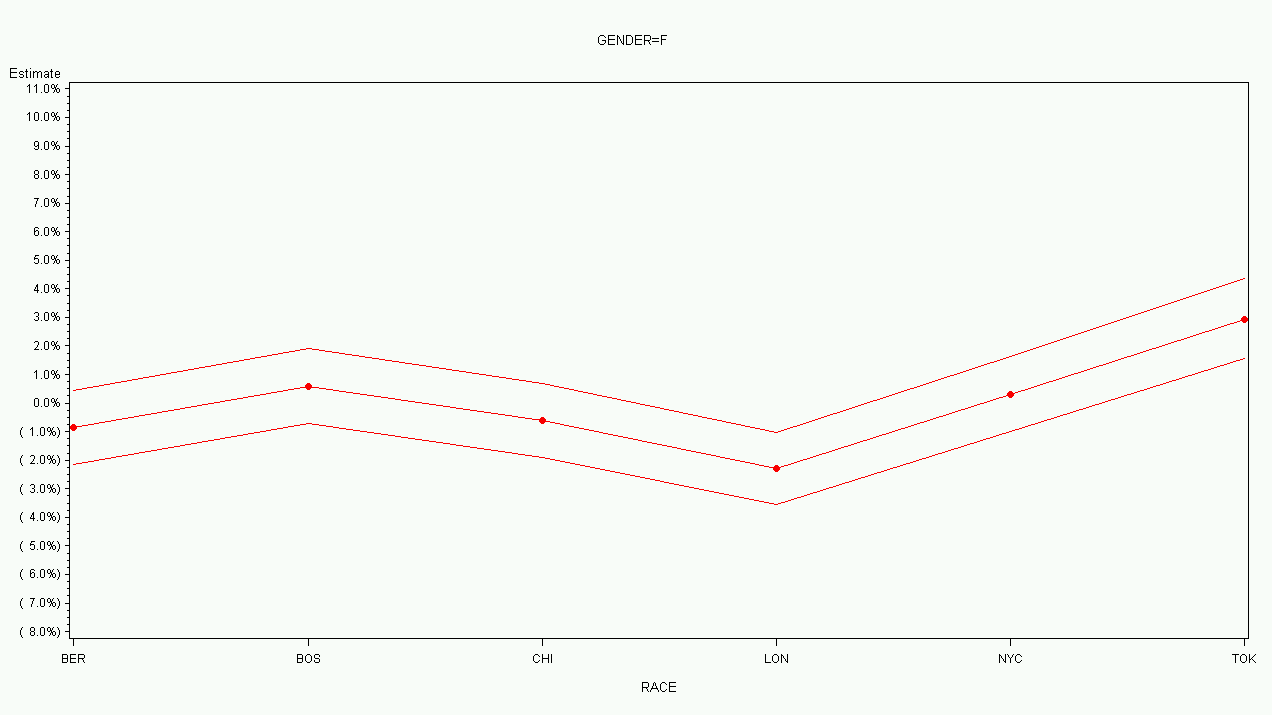

Supplement: S2 File — (ZIP) [file pone.0184024.s002.zip › Boston Marathon/Model 2 Race W.bmp]

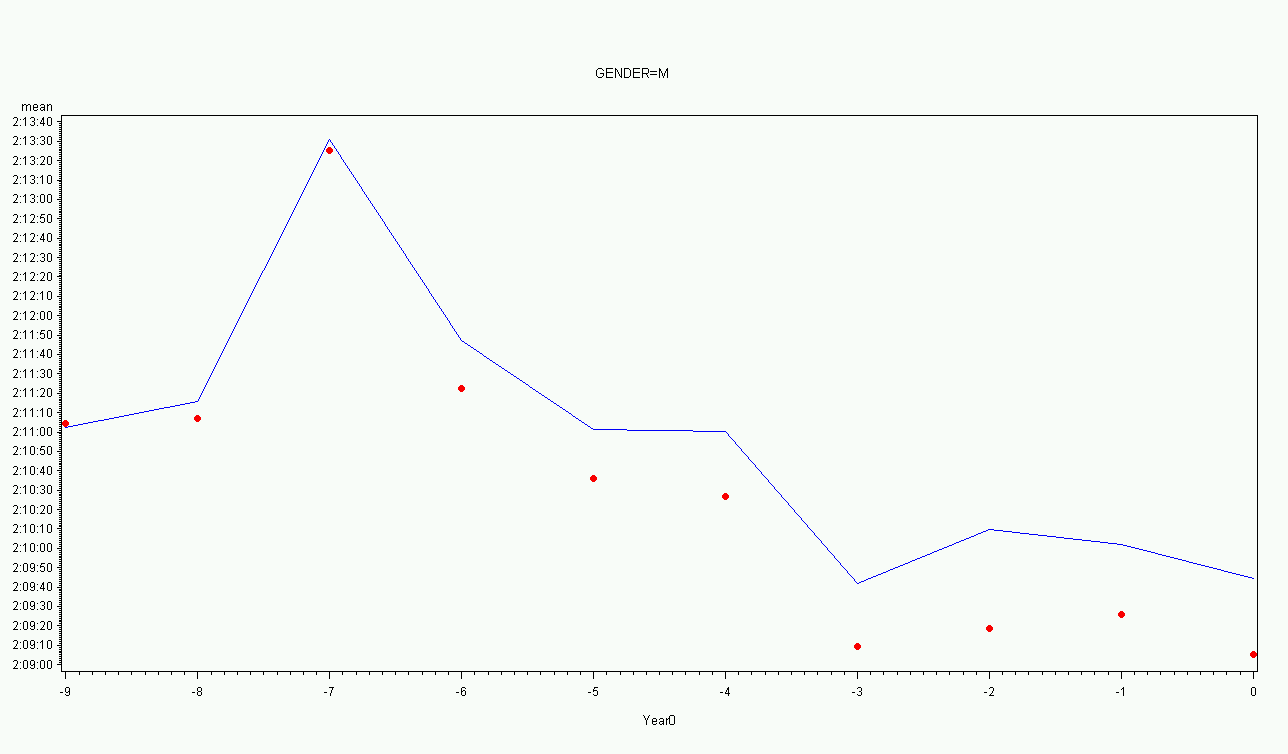

Supplement: S2 File — (ZIP) [file pone.0184024.s002.zip › Boston Marathon/Model 2 Time M with raw.bmp]

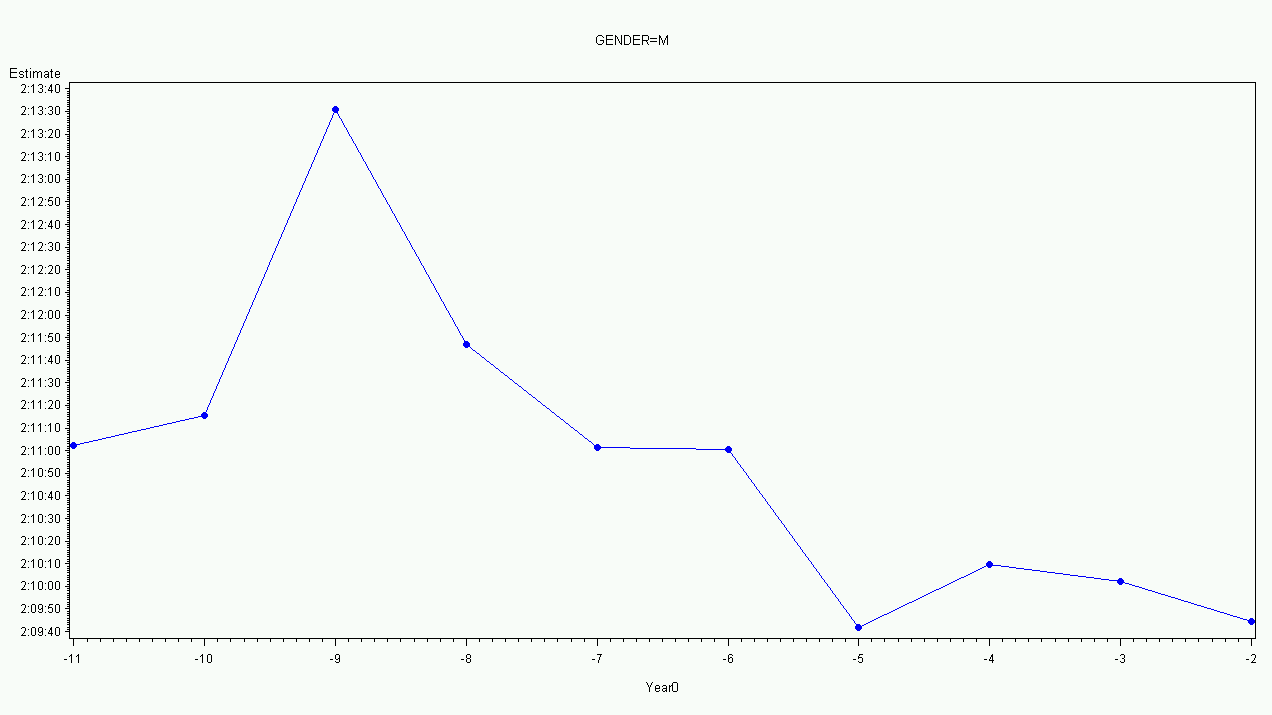

Supplement: S2 File — (ZIP) [file pone.0184024.s002.zip › Boston Marathon/Model 2 Time M.bmp]

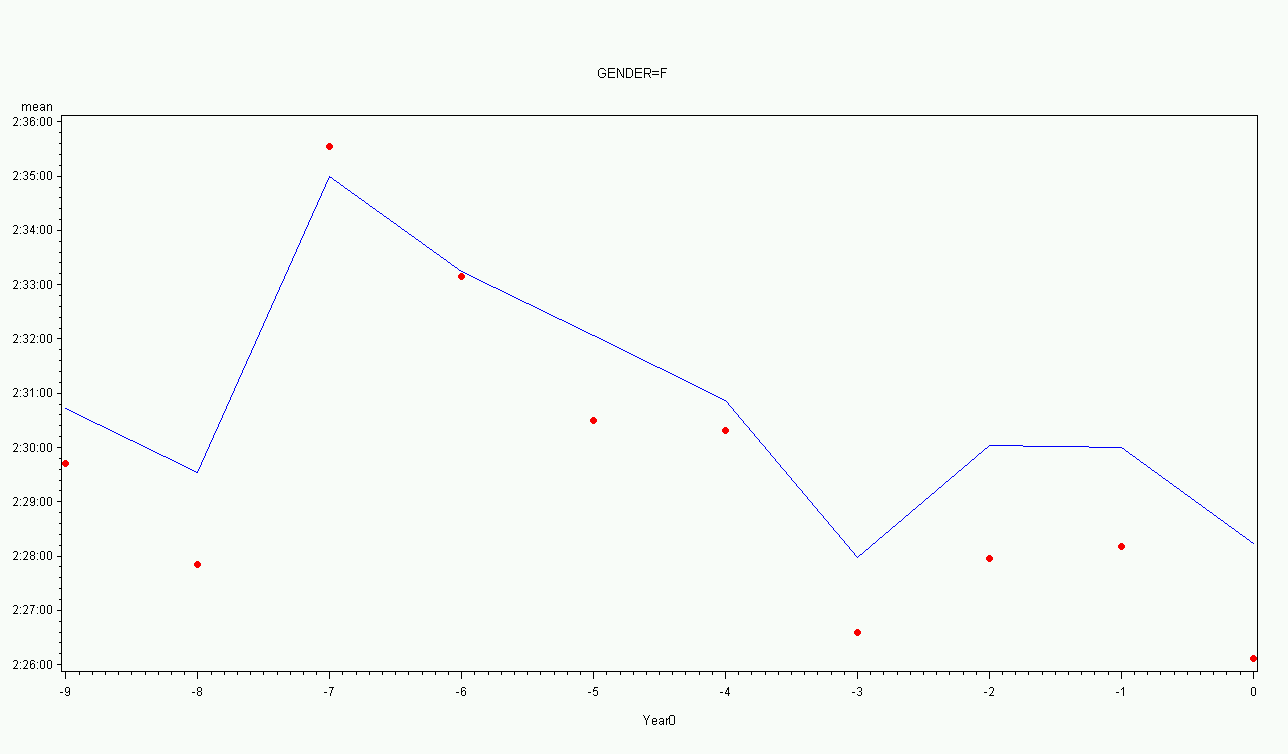

Supplement: S2 File — (ZIP) [file pone.0184024.s002.zip › Boston Marathon/Model 2 Time W with raw.bmp]

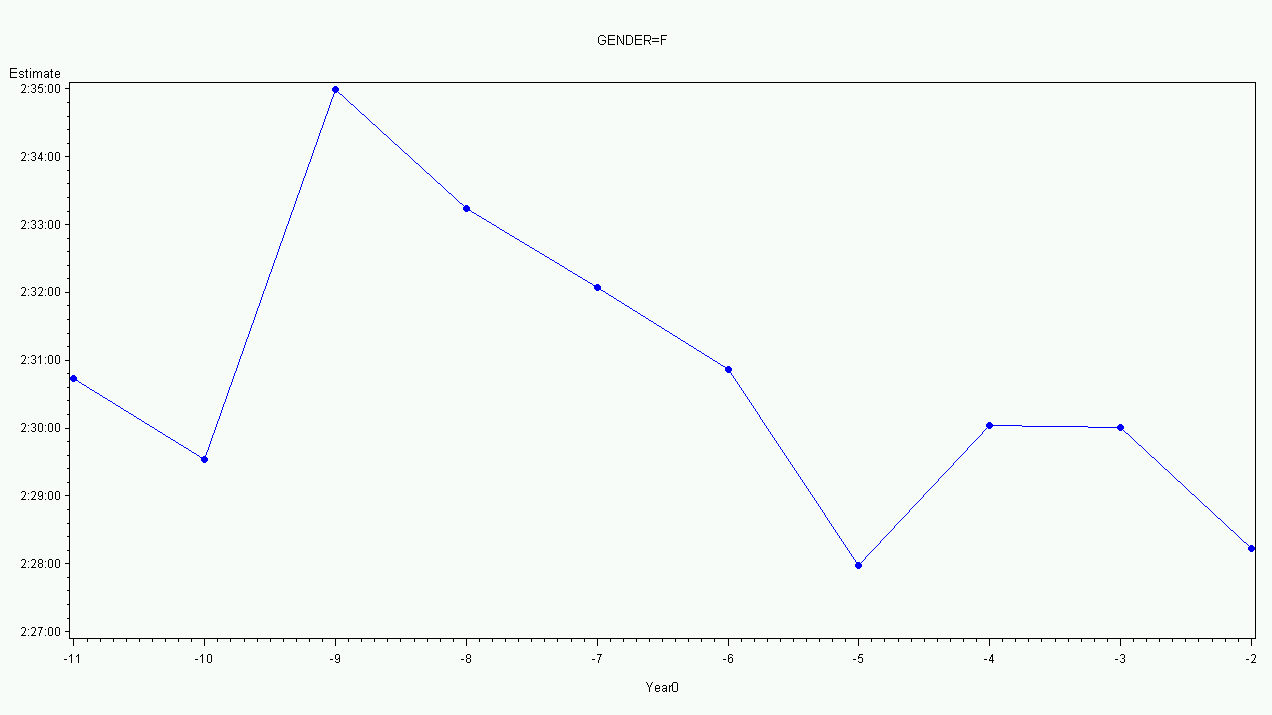

Supplement: S2 File — (ZIP) [file pone.0184024.s002.zip › Boston Marathon/Model 2 Time W.bmp]

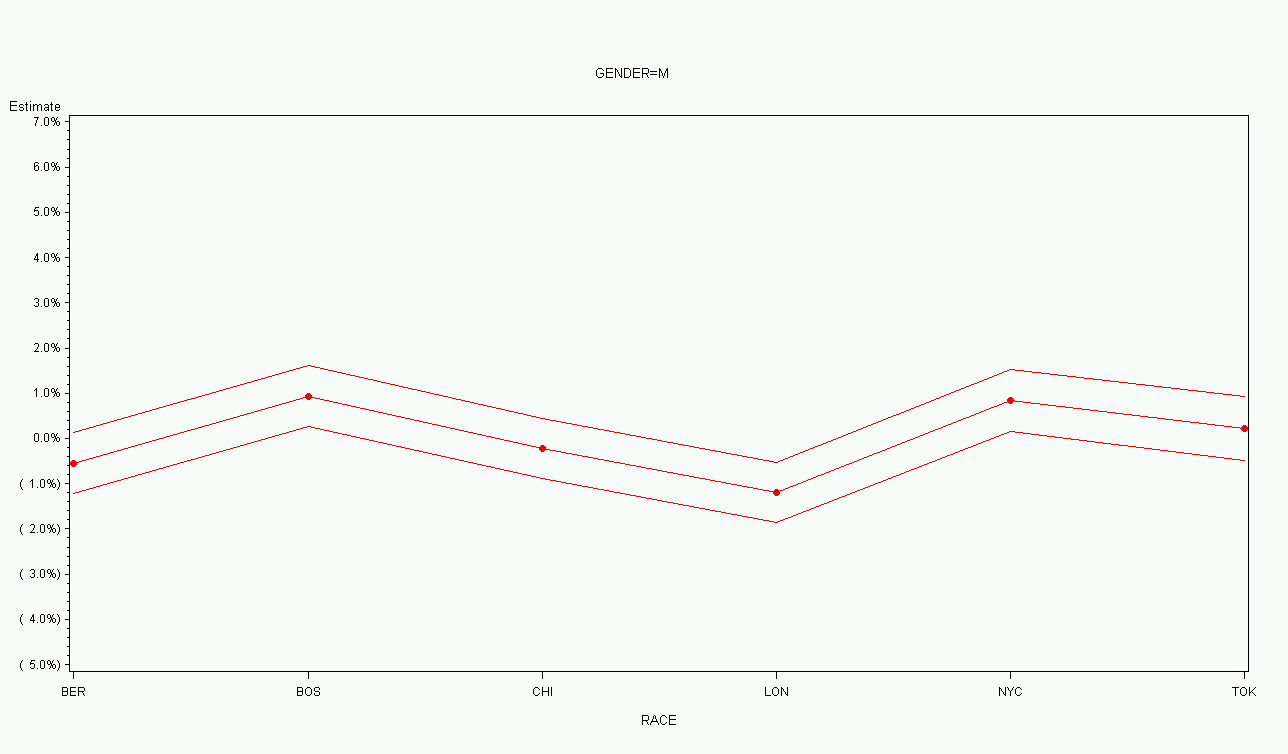

Supplement: S2 File — (ZIP) [file pone.0184024.s002.zip › Boston Marathon/Model 3 Race M.bmp]

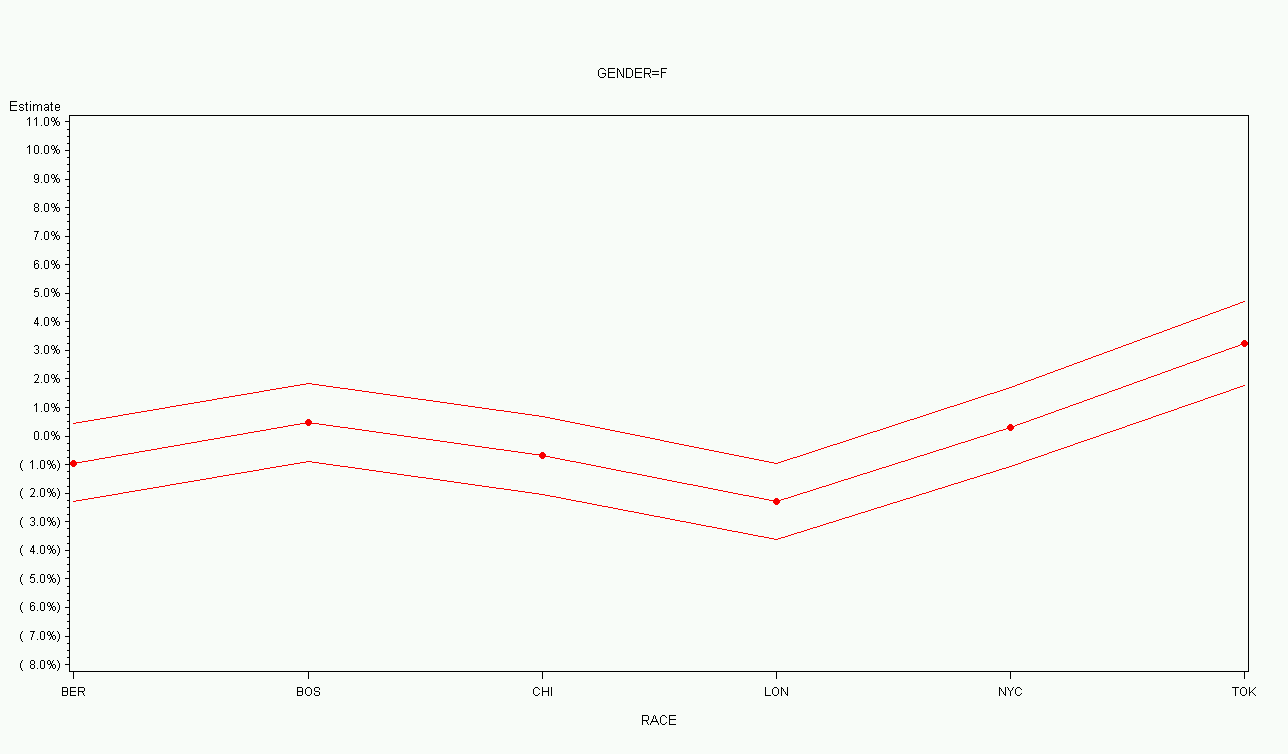

Supplement: S2 File — (ZIP) [file pone.0184024.s002.zip › Boston Marathon/Model 3 Race W.bmp]

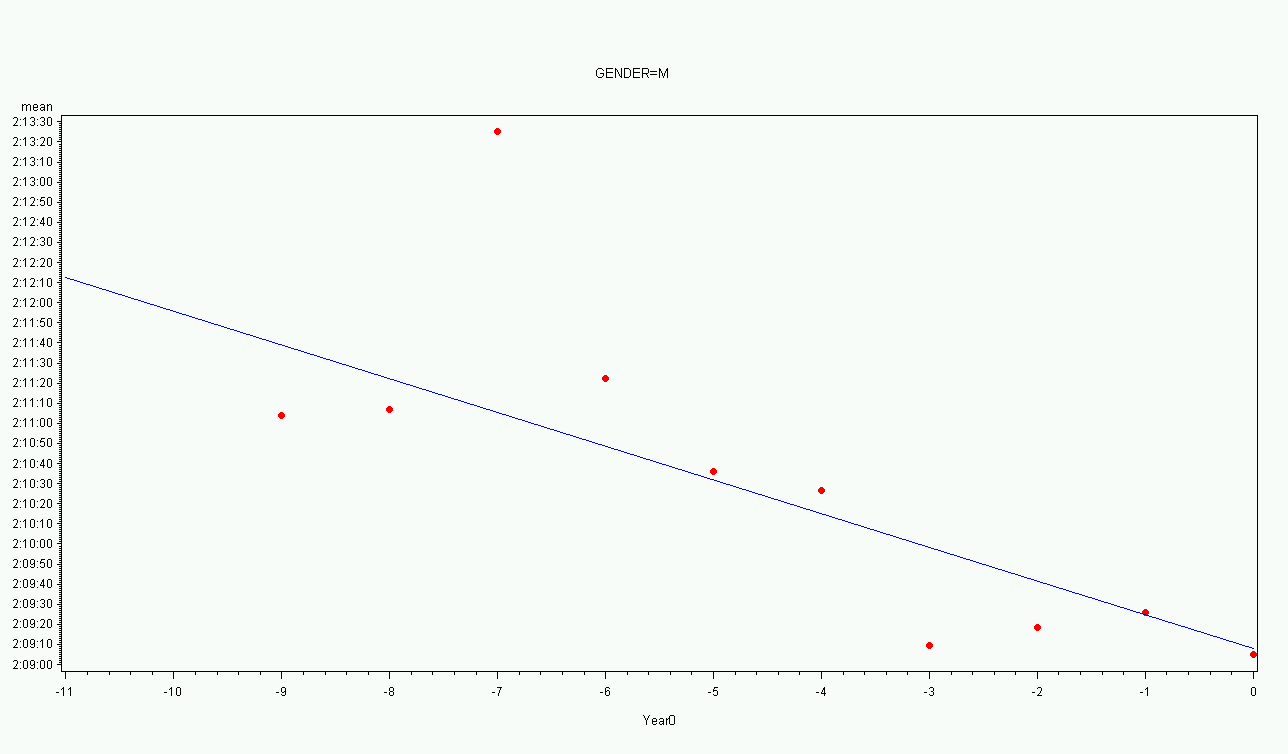

Supplement: S2 File — (ZIP) [file pone.0184024.s002.zip › Boston Marathon/Model 3 Time M.bmp]

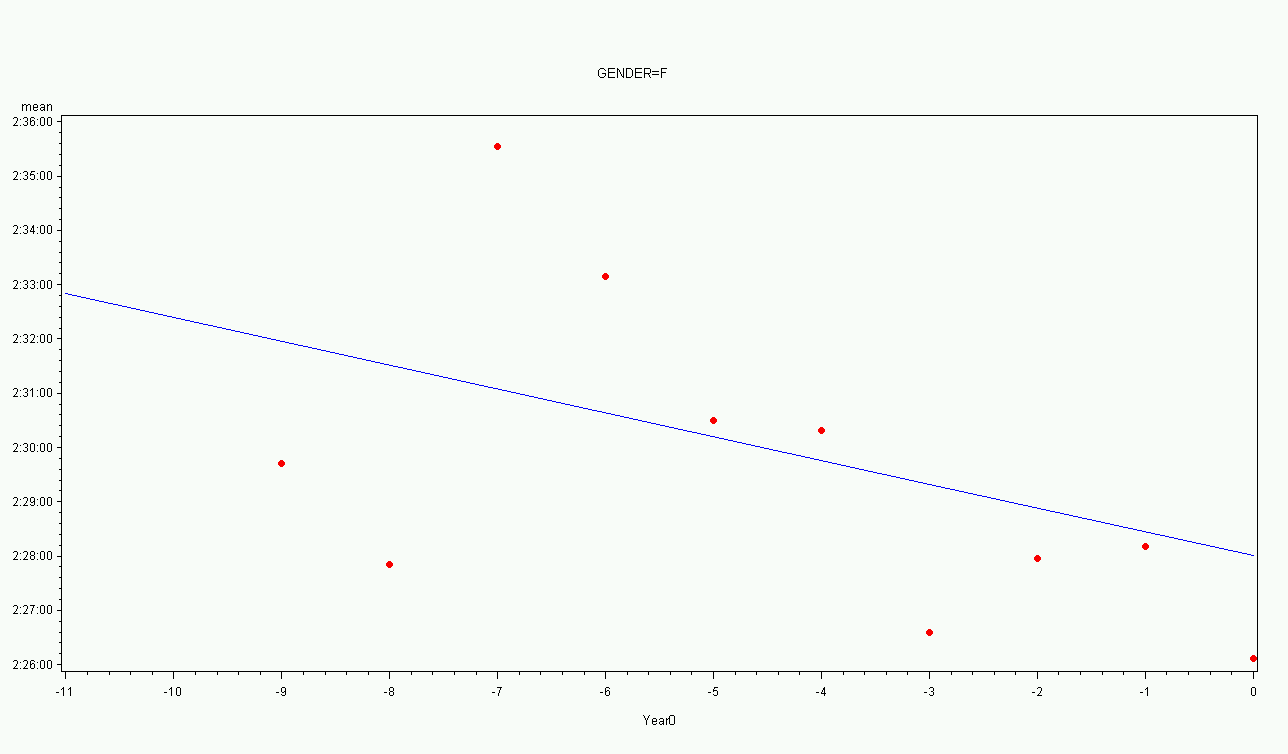

Supplement: S2 File — (ZIP) [file pone.0184024.s002.zip › Boston Marathon/Model 3 Time W.bmp]

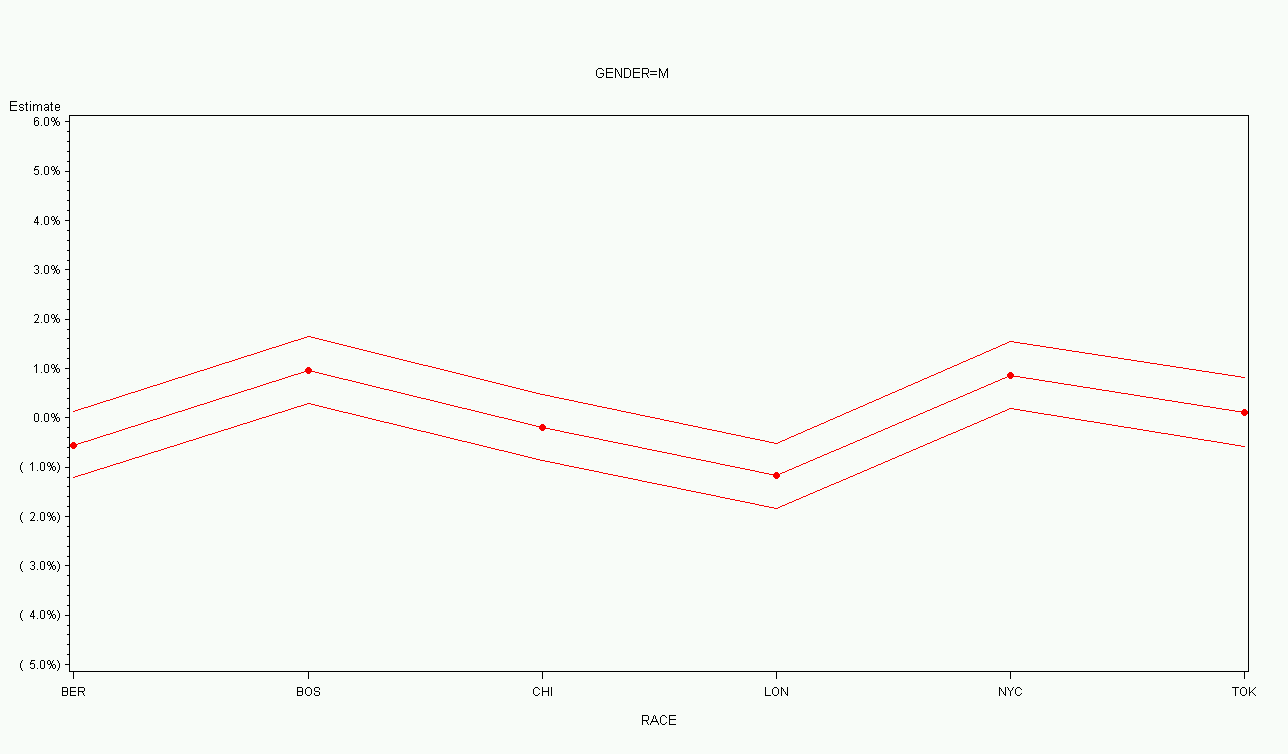

Supplement: S2 File — (ZIP) [file pone.0184024.s002.zip › Boston Marathon/Model 4 Race M.bmp]

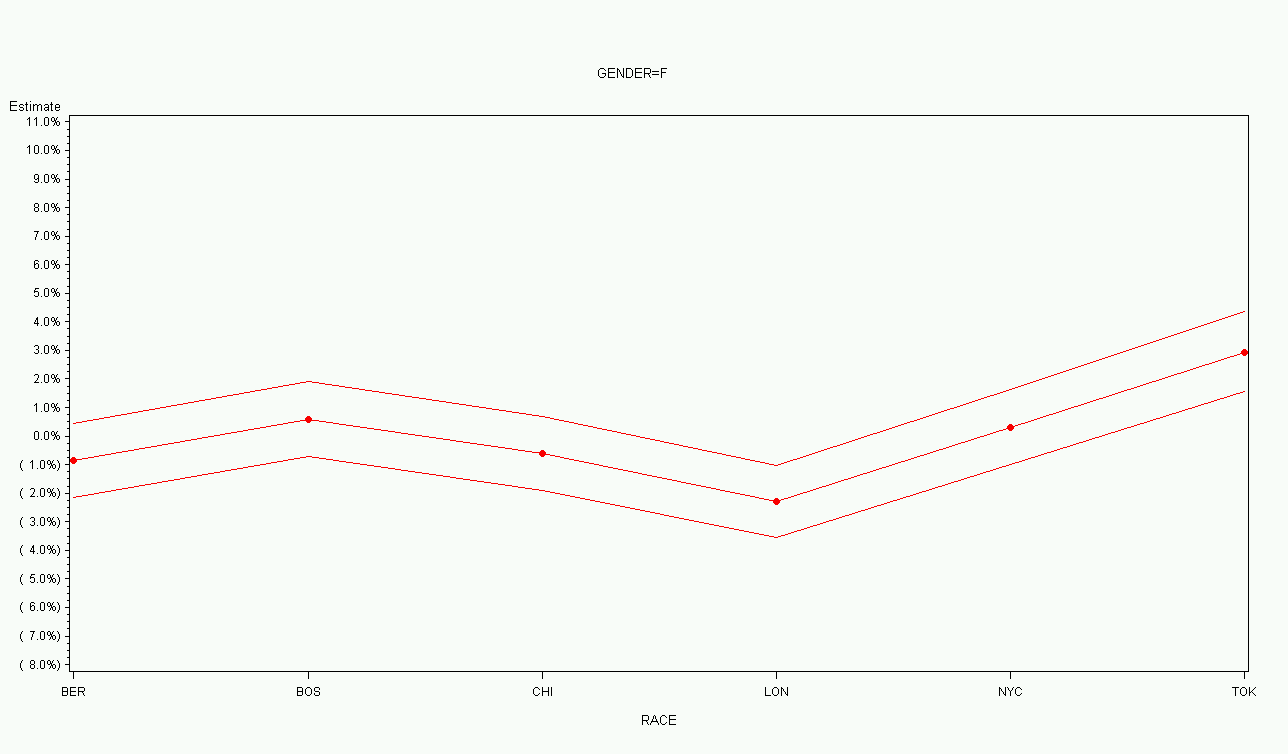

Supplement: S2 File — (ZIP) [file pone.0184024.s002.zip › Boston Marathon/Model 4 Race W.bmp]

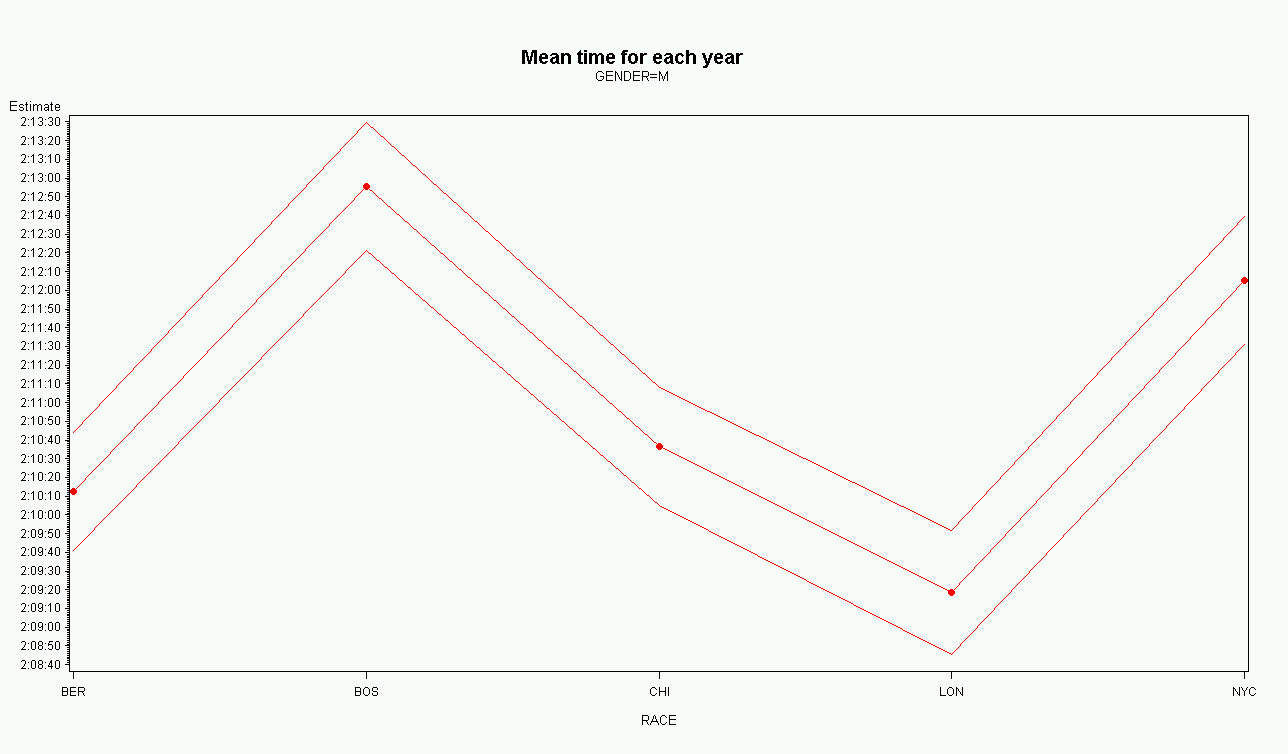

Supplement: S2 File — (ZIP) [file pone.0184024.s002.zip › Boston Marathon/model 5 Race M no 2011 no Tok.bmp]

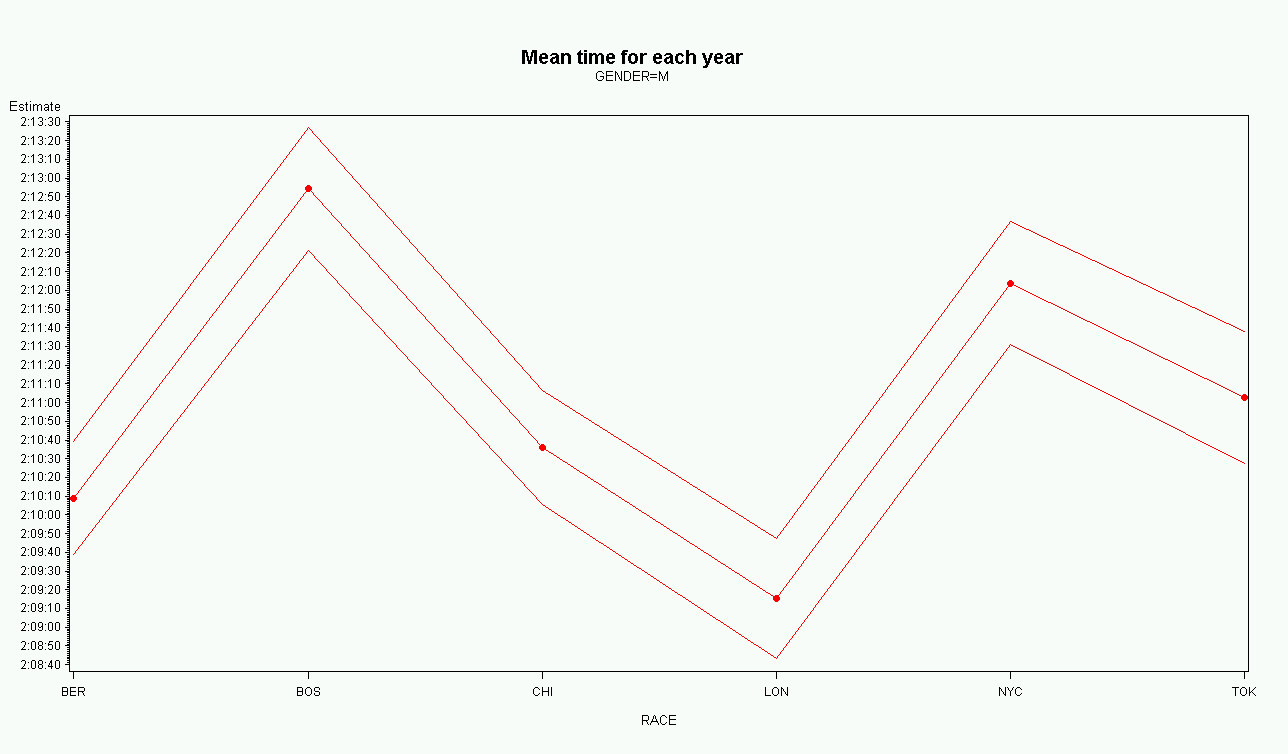

Supplement: S2 File — (ZIP) [file pone.0184024.s002.zip › Boston Marathon/model 5 Race M no 2011.bmp]

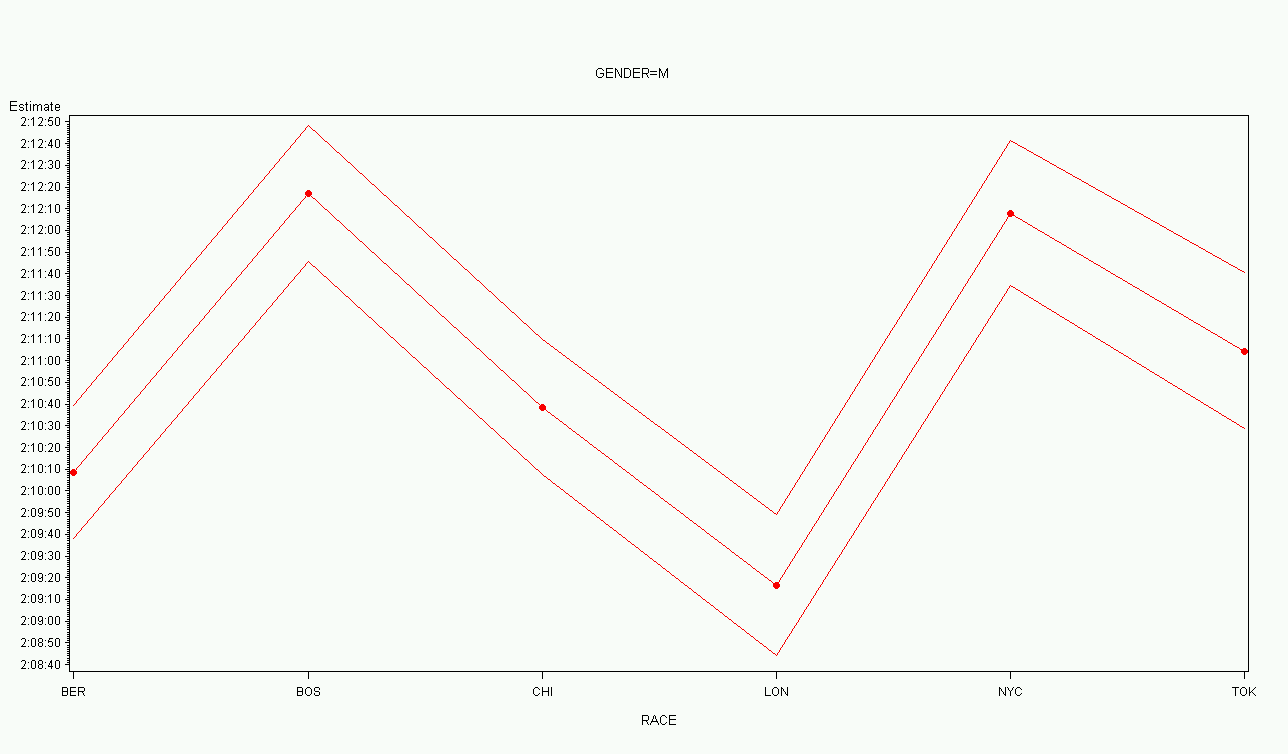

Supplement: S2 File — (ZIP) [file pone.0184024.s002.zip › Boston Marathon/model 5 Race M.bmp]

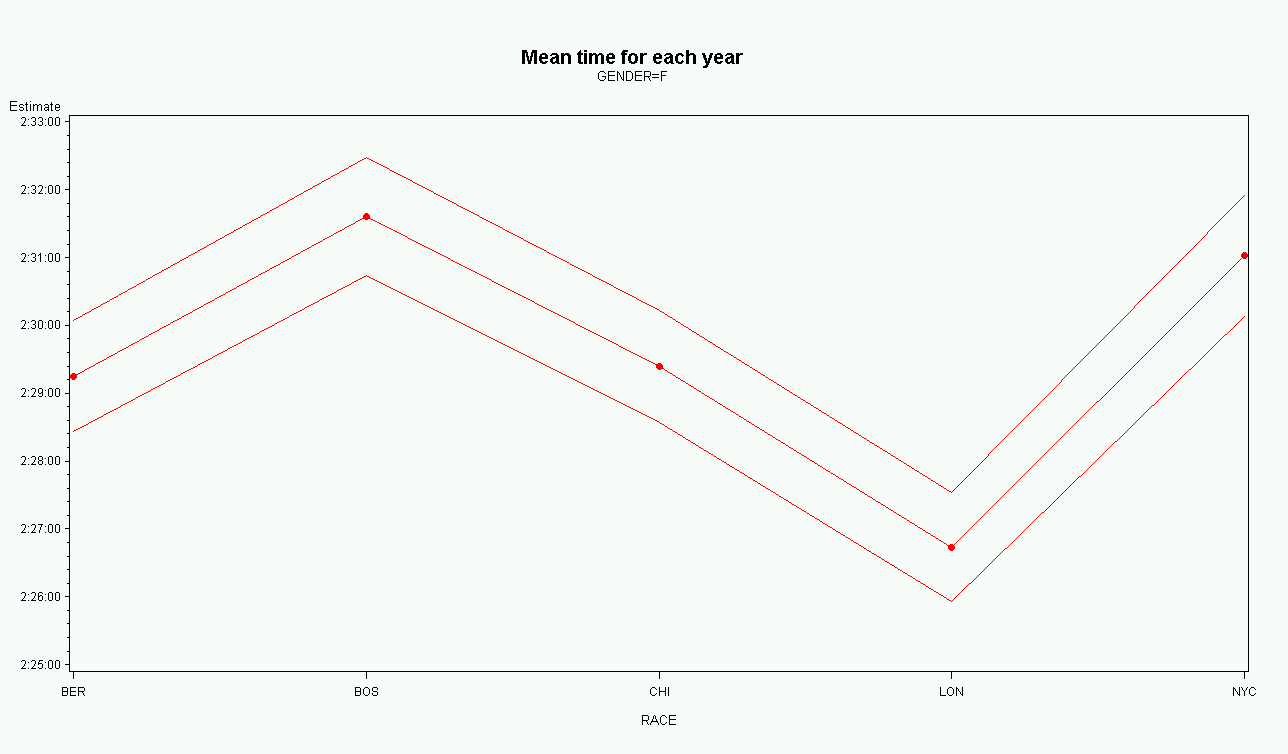

Supplement: S2 File — (ZIP) [file pone.0184024.s002.zip › Boston Marathon/model 5 Race W no 2011 no Tok.bmp]

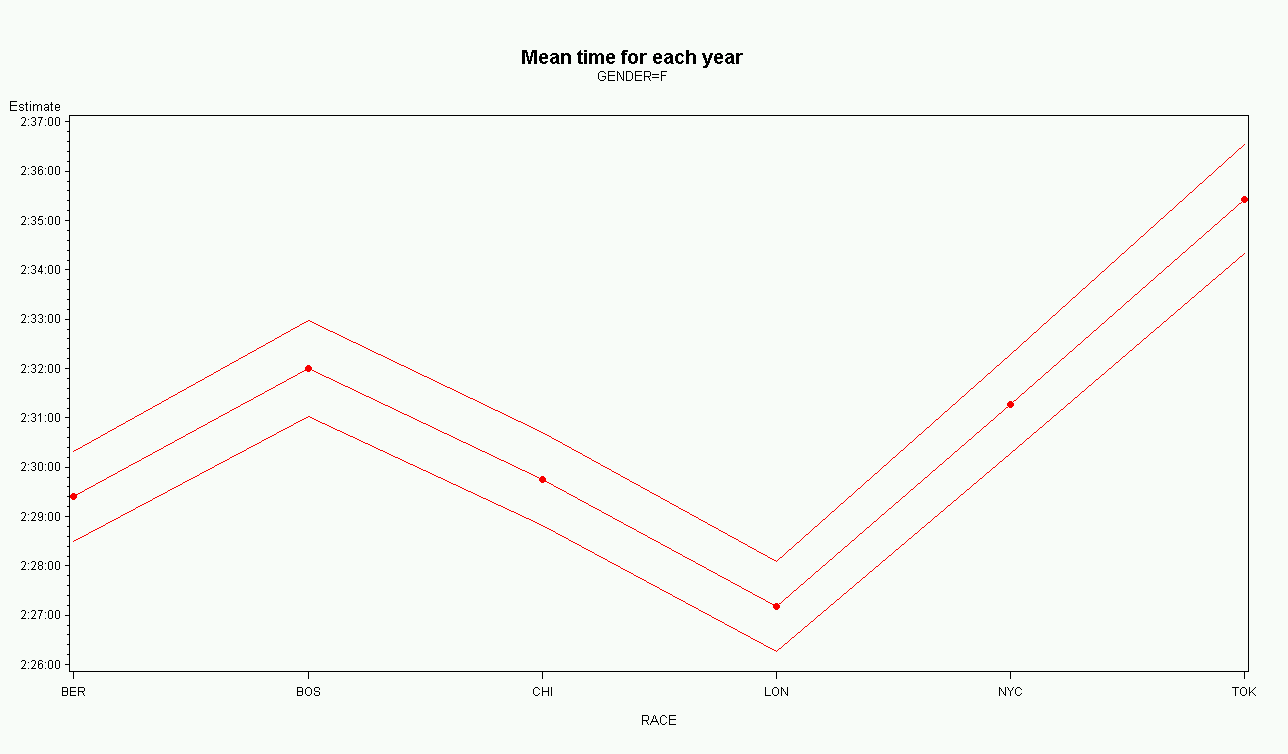

Supplement: S2 File — (ZIP) [file pone.0184024.s002.zip › Boston Marathon/model 5 Race W no 2011.bmp]

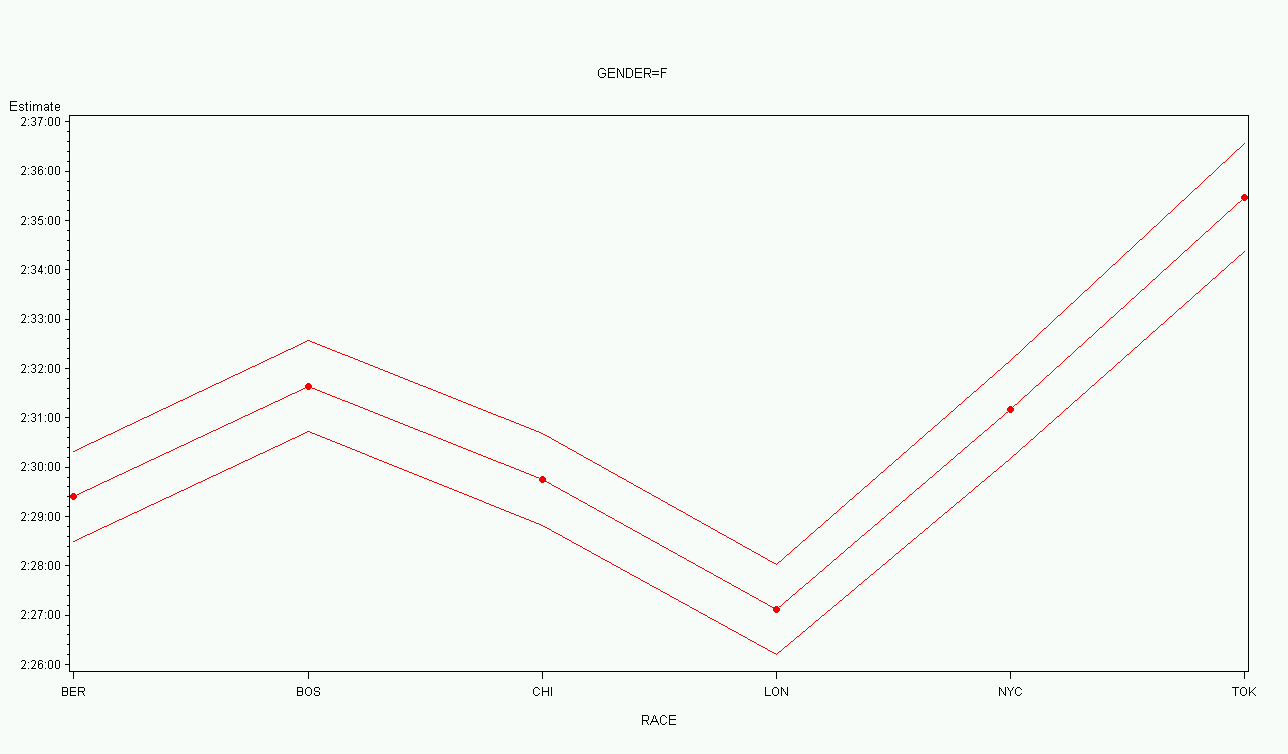

Supplement: S2 File — (ZIP) [file pone.0184024.s002.zip › Boston Marathon/model 5 Race W.bmp]

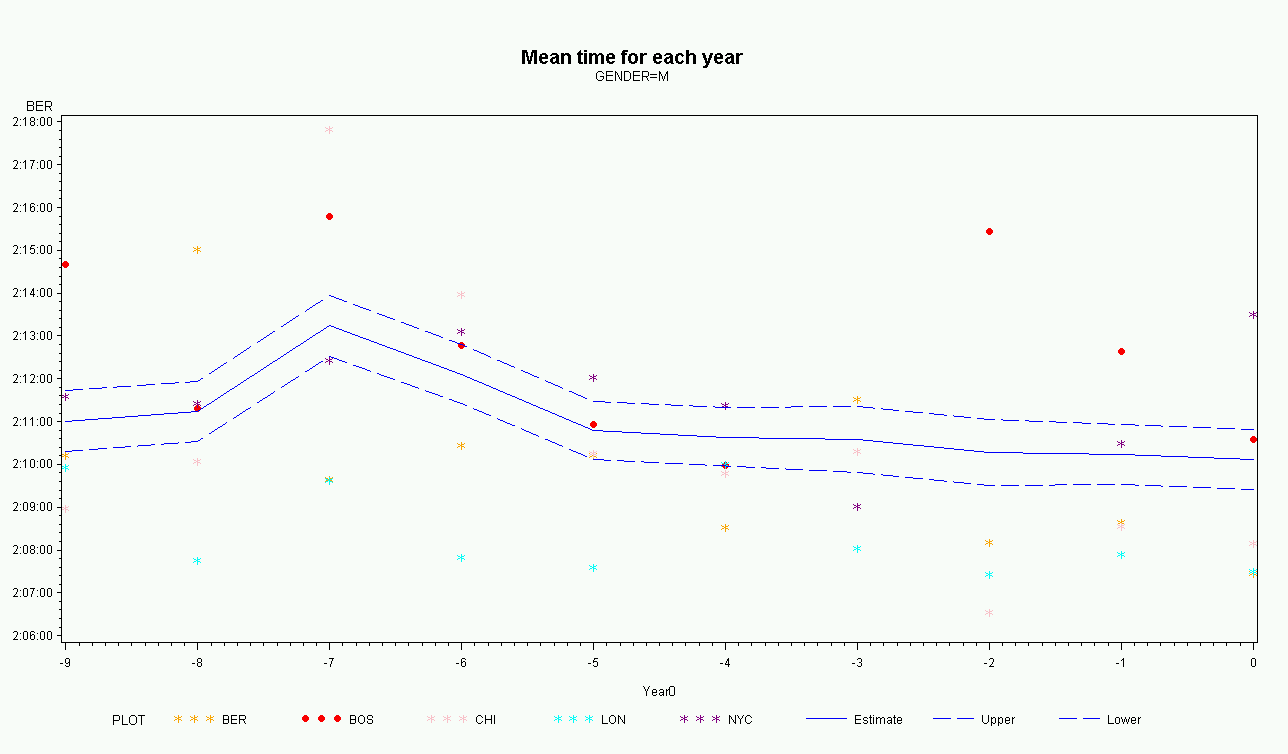

Supplement: S2 File — (ZIP) [file pone.0184024.s002.zip › Boston Marathon/model 5 Time M no 2011 no Tok.bmp]

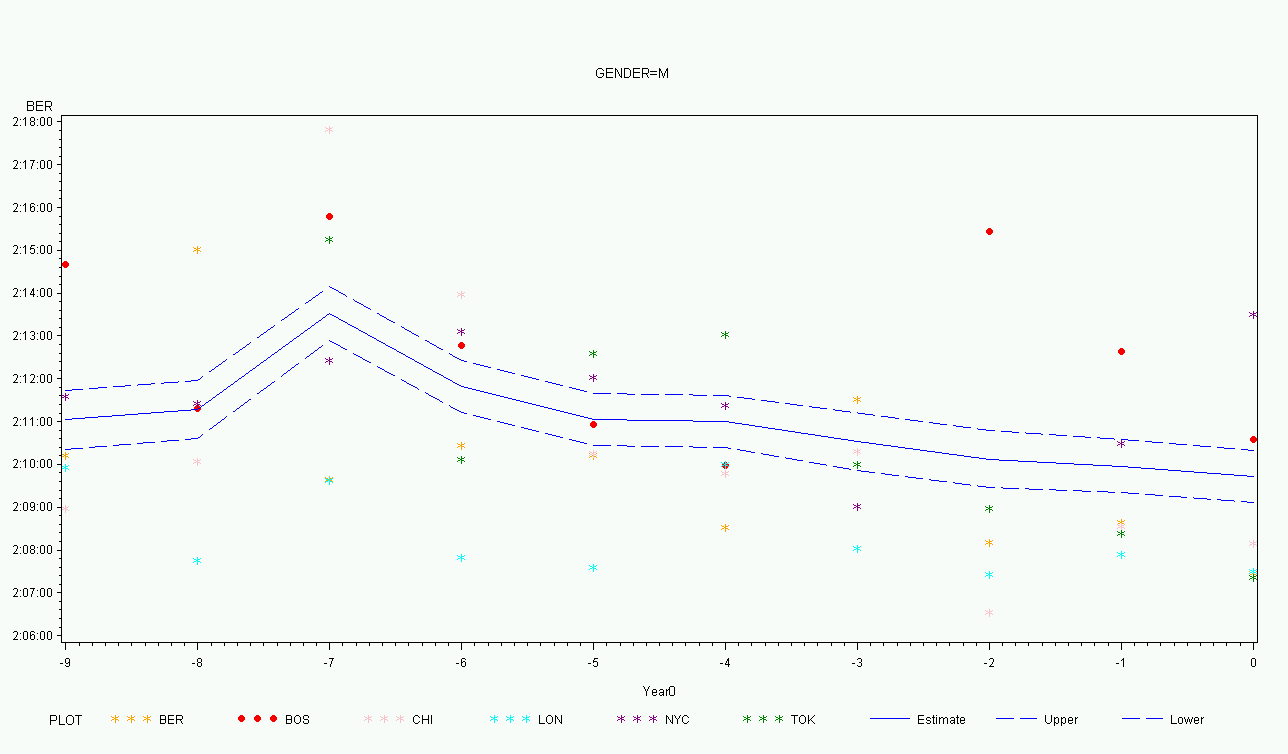

Supplement: S2 File — (ZIP) [file pone.0184024.s002.zip › Boston Marathon/model 5 Time M with races no 2011.bmp]

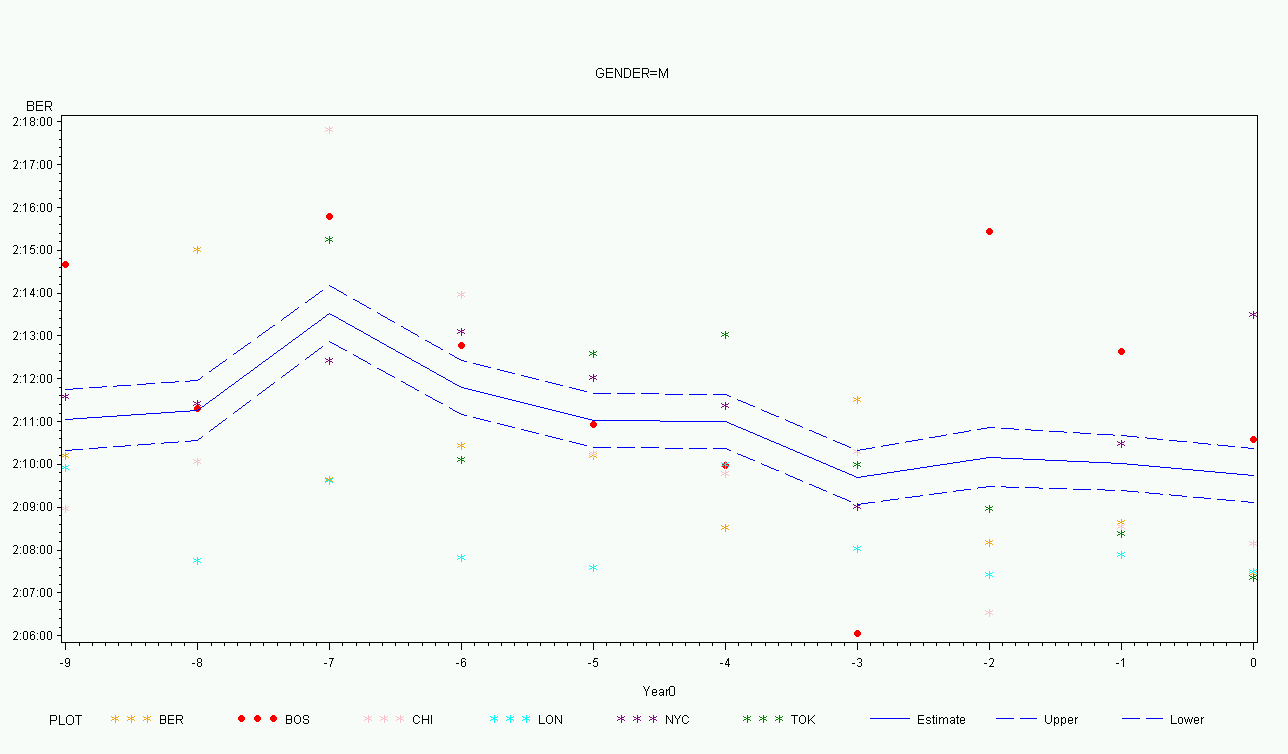

Supplement: S2 File — (ZIP) [file pone.0184024.s002.zip › Boston Marathon/model 5 Time M with races.bmp]

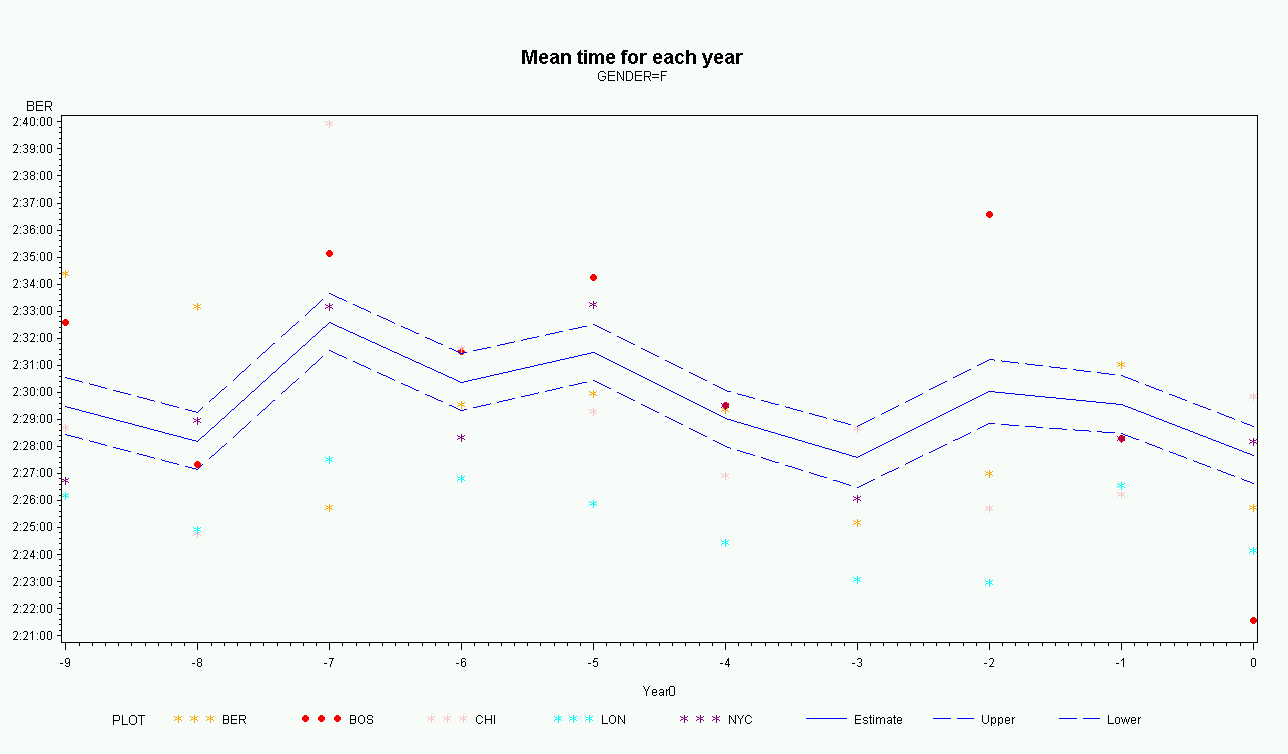

Supplement: S2 File — (ZIP) [file pone.0184024.s002.zip › Boston Marathon/model 5 Time W no 2011 no Tok.bmp]

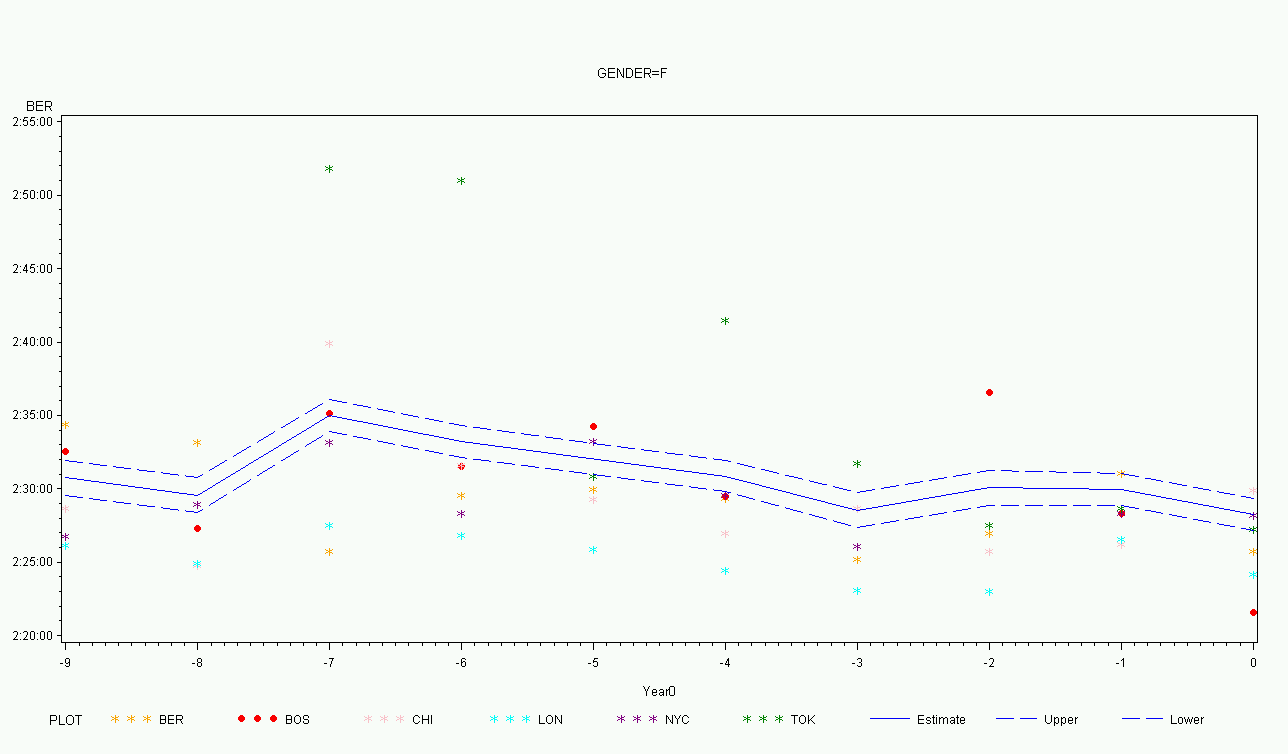

Supplement: S2 File — (ZIP) [file pone.0184024.s002.zip › Boston Marathon/model 5 Time W with races no 2011.bmp]

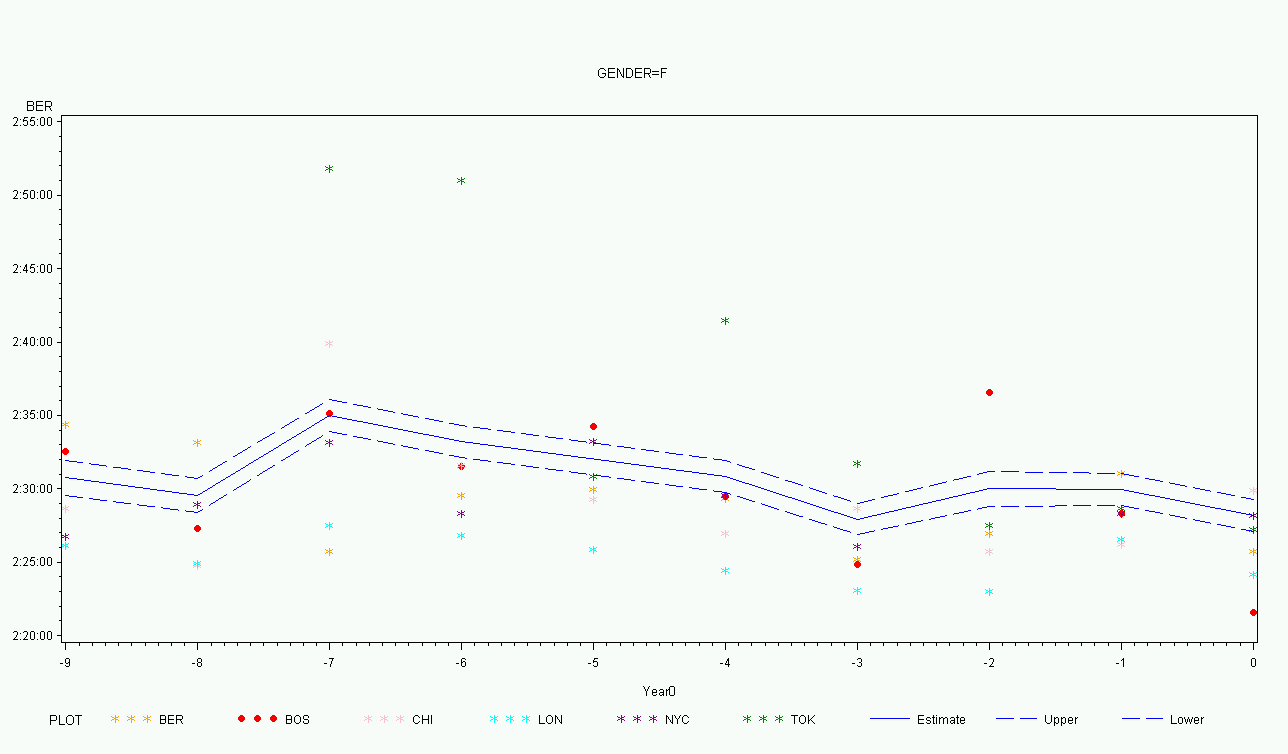

Supplement: S2 File — (ZIP) [file pone.0184024.s002.zip › Boston Marathon/model 5 Time W with races.bmp]

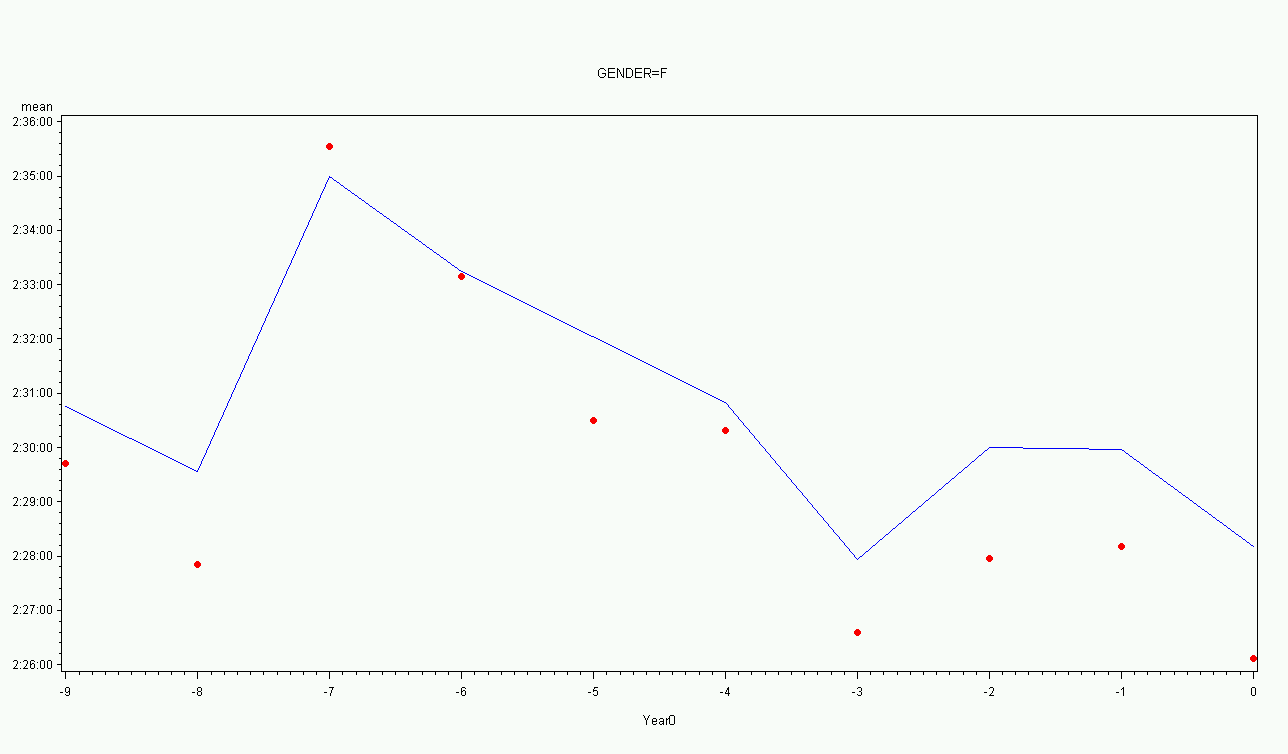

Supplement: S2 File — (ZIP) [file pone.0184024.s002.zip › Boston Marathon/model 5 Time W.bmp]

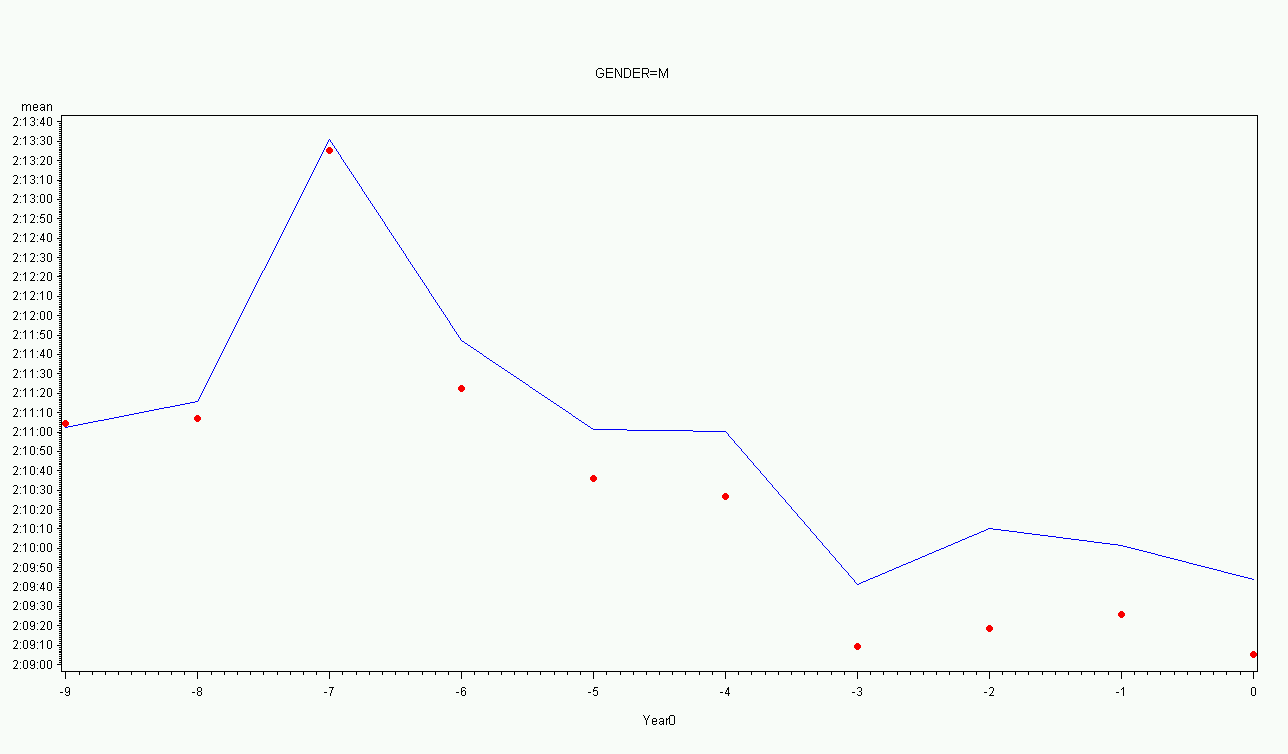

Supplement: S2 File — (ZIP) [file pone.0184024.s002.zip › Boston Marathon/model 5 TimeMW.bmp]

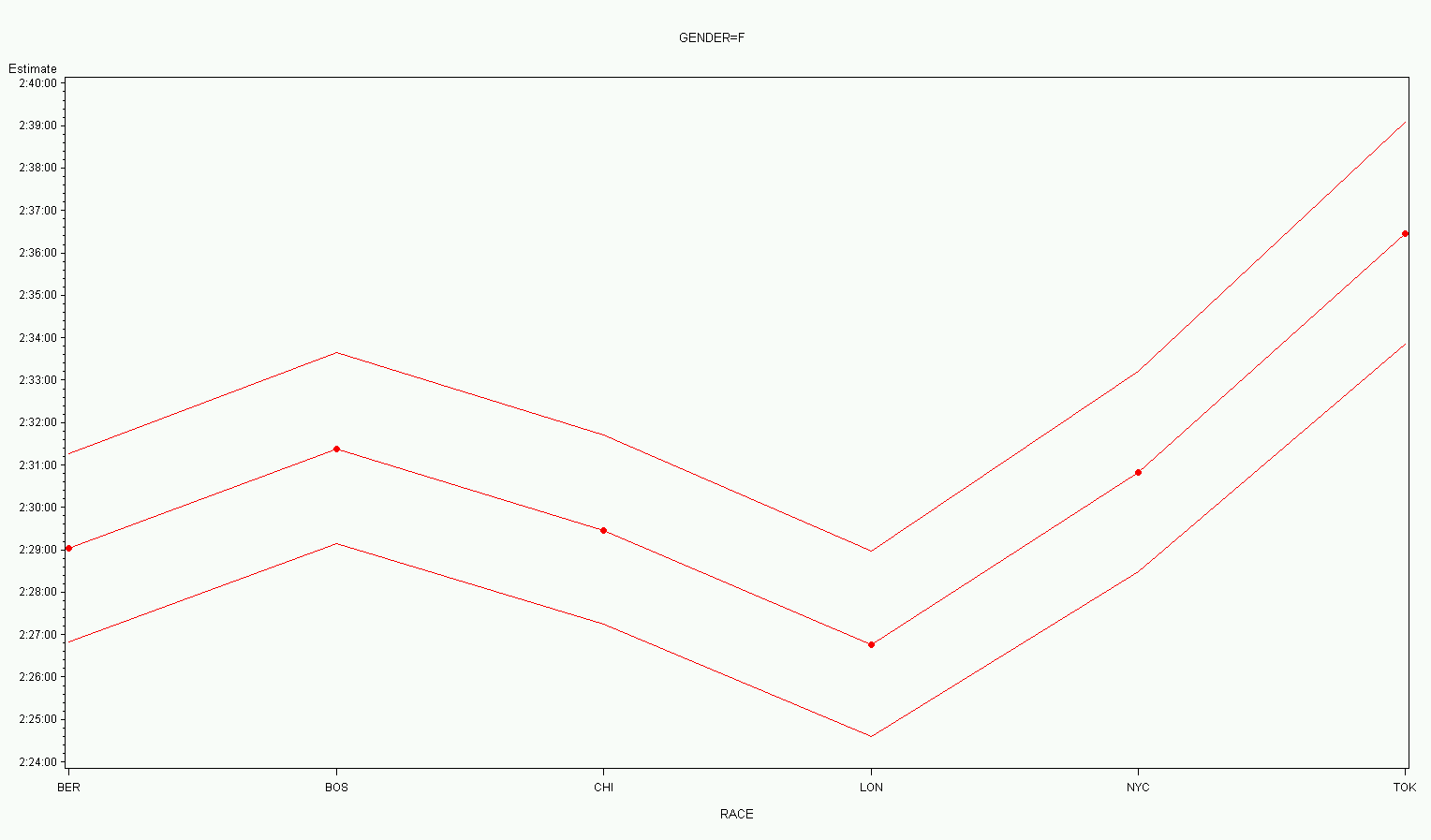

Supplement: S2 File — (ZIP) [file pone.0184024.s002.zip › Boston Marathon/model 6 Race F.bmp]

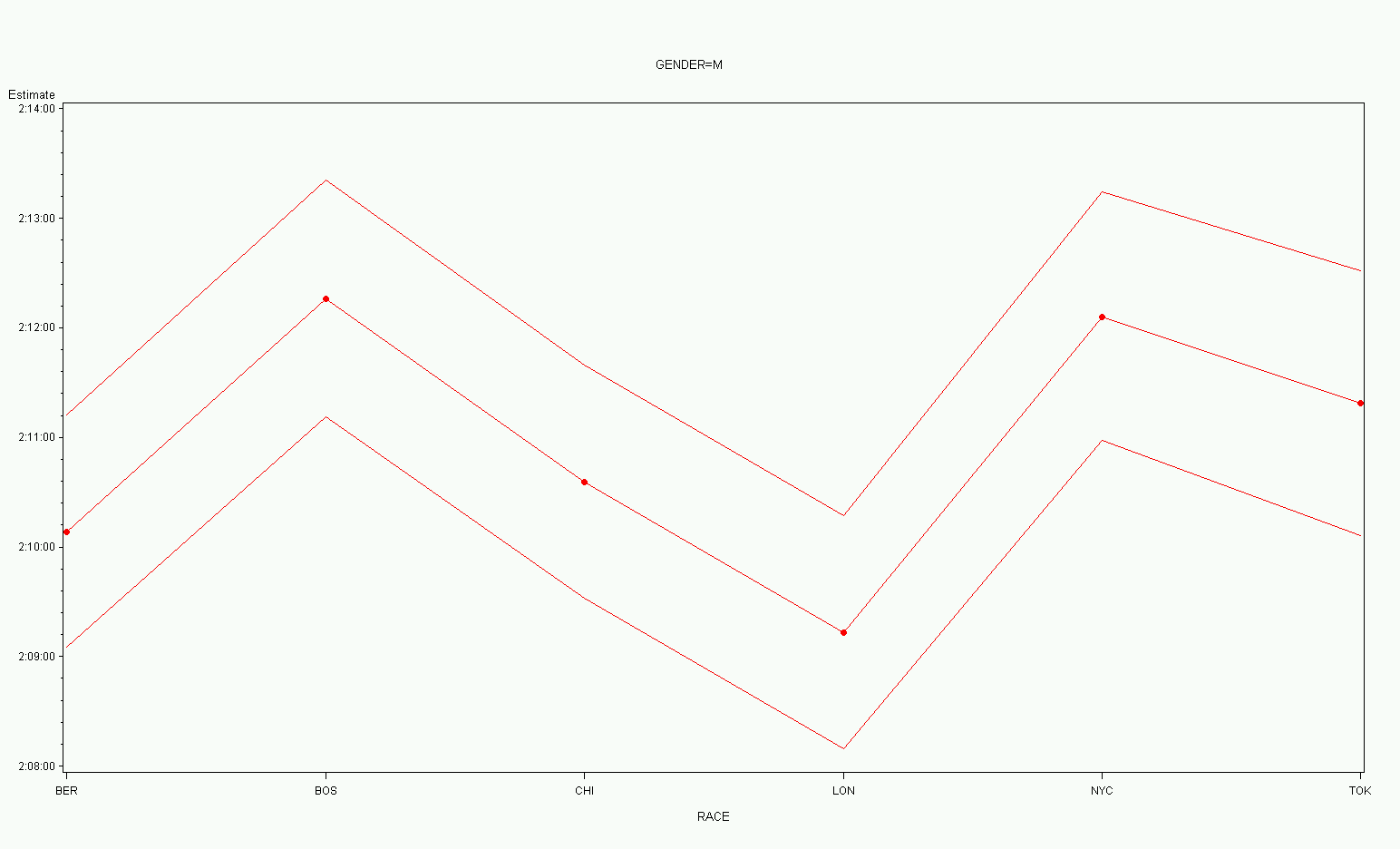

Supplement: S2 File — (ZIP) [file pone.0184024.s002.zip › Boston Marathon/model 6 Race W.bmp]

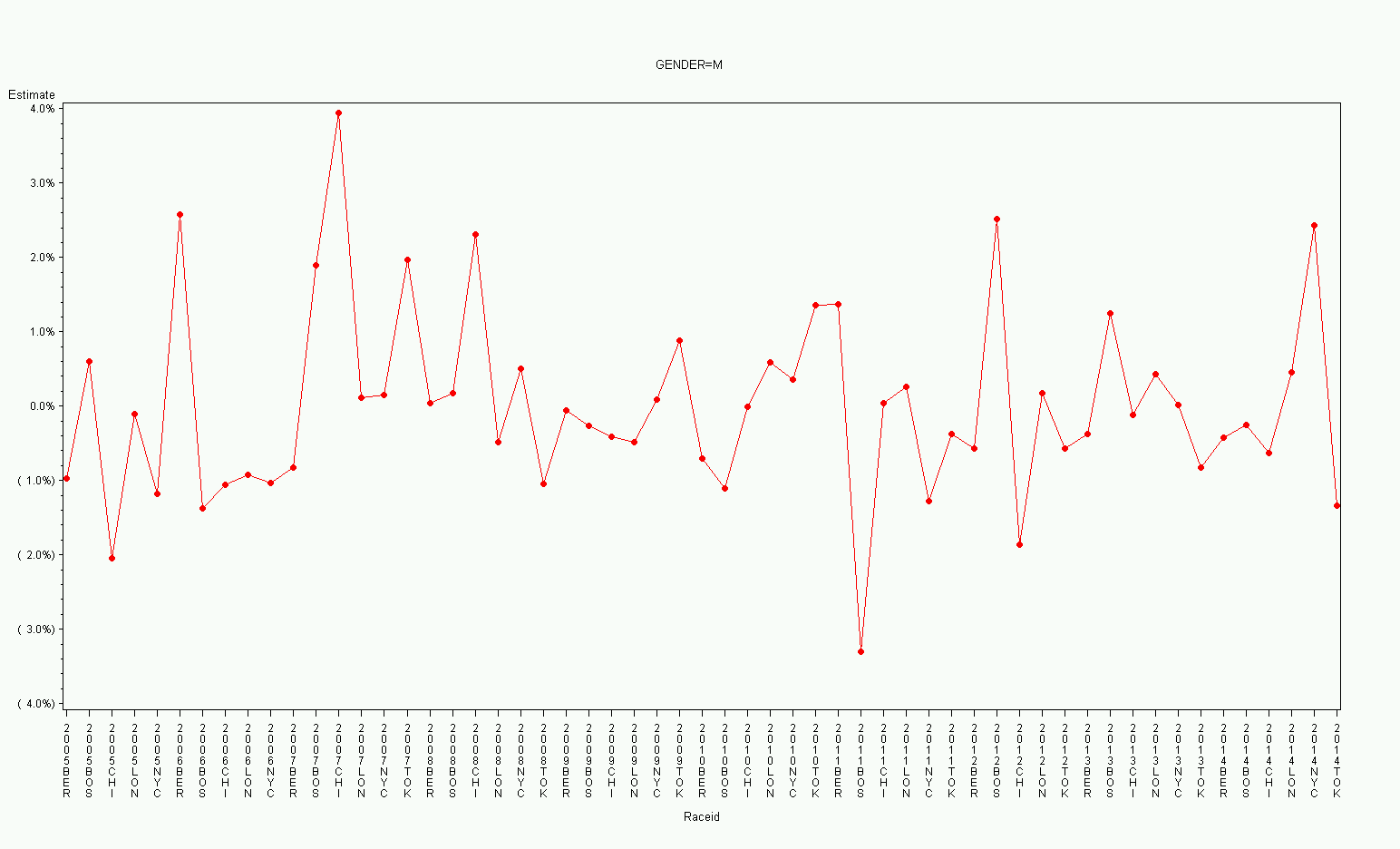

Supplement: S2 File — (ZIP) [file pone.0184024.s002.zip › Boston Marathon/model 6 RaceID M.bmp]

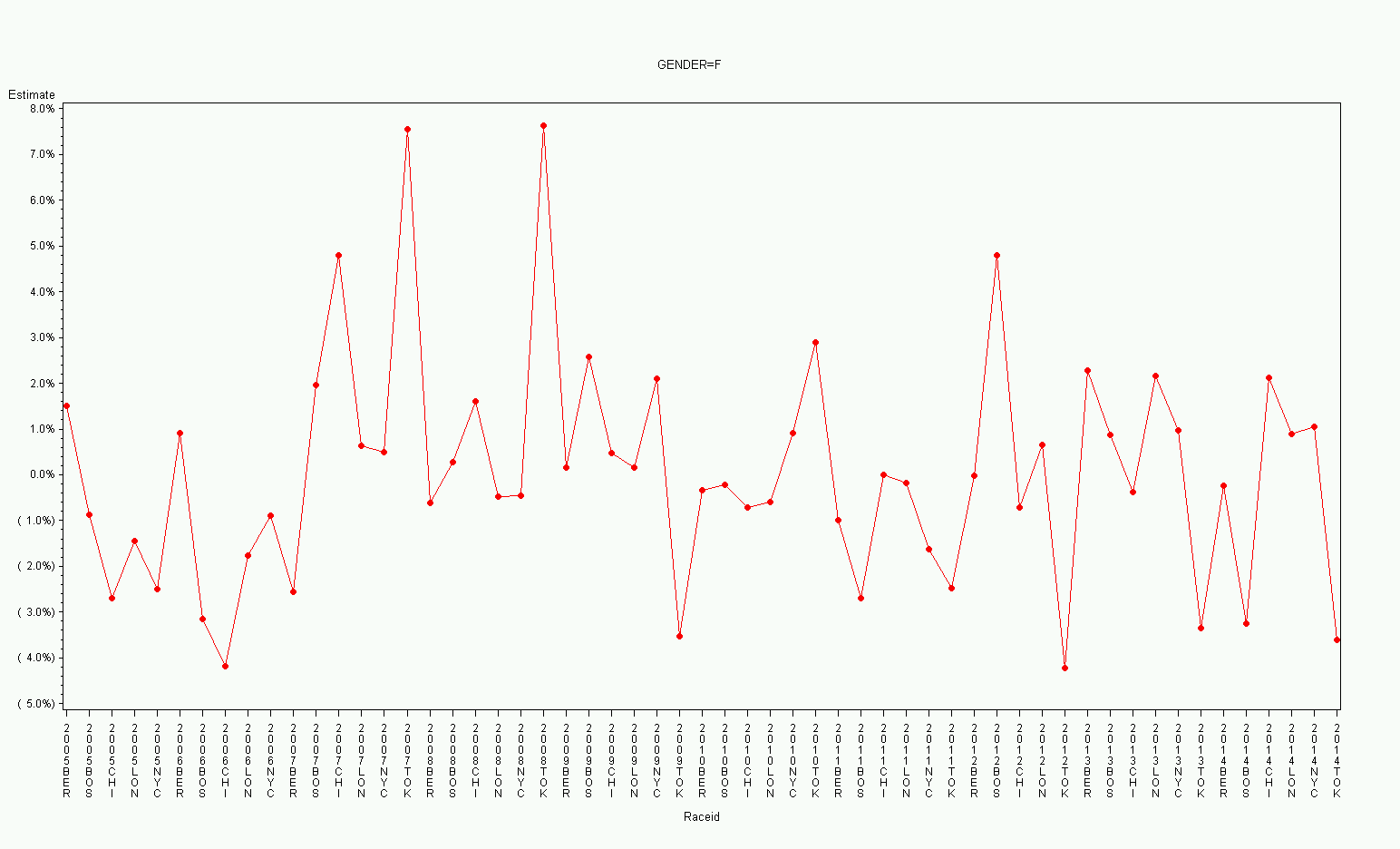

Supplement: S2 File — (ZIP) [file pone.0184024.s002.zip › Boston Marathon/model 6 RaceID W.bmp]

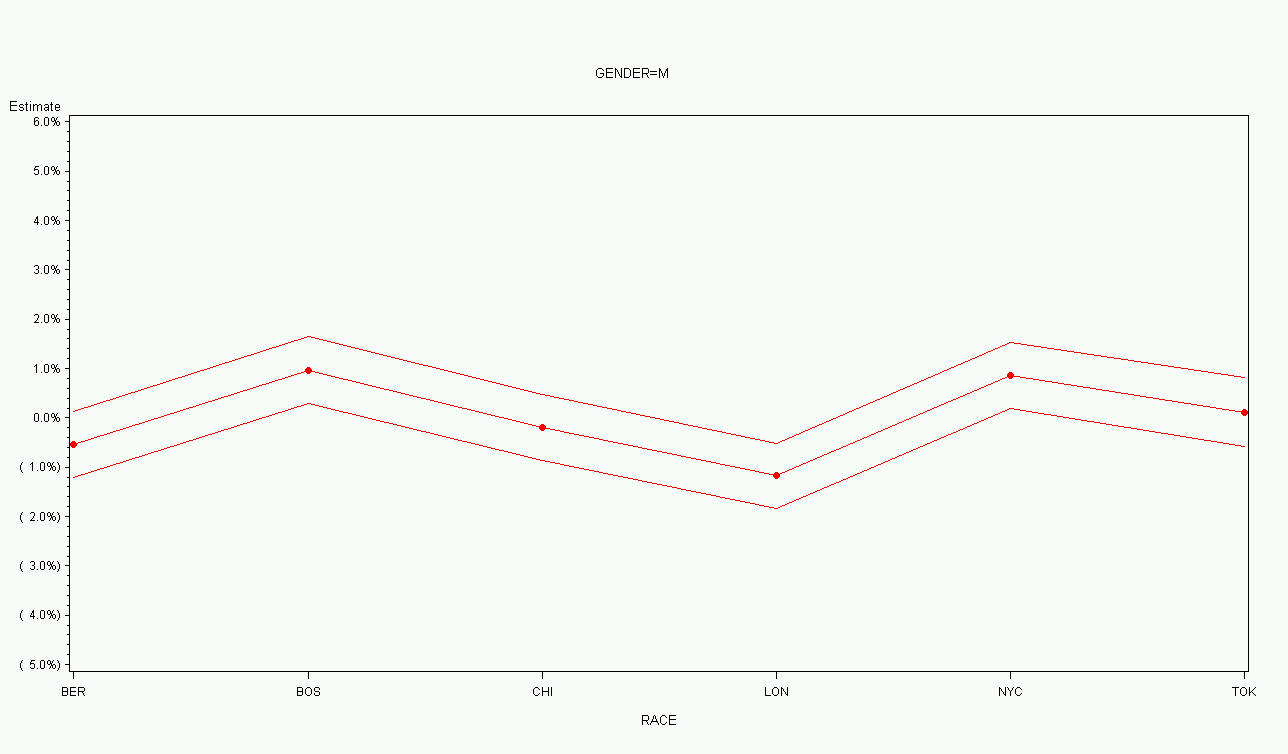

Supplement: S2 File — (ZIP) [file pone.0184024.s002.zip › Boston Marathon/Race M Model 1.bmp]

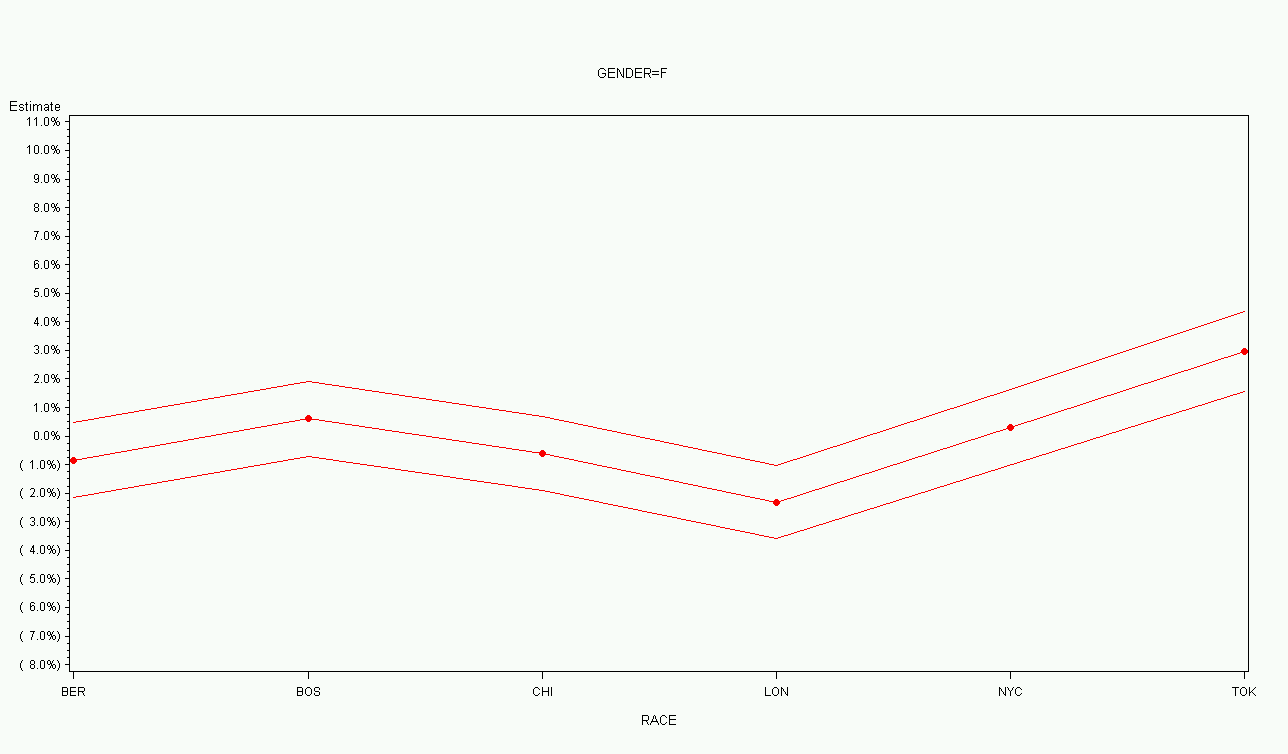

Supplement: S2 File — (ZIP) [file pone.0184024.s002.zip › Boston Marathon/Race W Model 1.bmp]
